# Genetic architecture of grain yield in bread wheat based on genome-wide association studies

**DOI:** 10.1186/s12870-019-1781-3

**Published:** 2019-04-29

**Authors:** Faji Li, Weie Wen, Jindong Liu, Yong Zhang, Shuanghe Cao, Zhonghu He, Awais Rasheed, Hui Jin, Chi Zhang, Jun Yan, Pingzhi Zhang, Yingxiu Wan, Xianchun Xia

**Affiliations:** 1 College of Agronomy, Xinjiang Agricultural University, Urumqi, 830052 Xinjiang China; 2 Institute of Crop Sciences, National Wheat Improvement Center, Chinese Academy of Agricultural Sciences (CAAS), 12 Zhongguancun South Street, Beijing, 100081 China; 3 International Maize and Wheat Improvement Center (CIMMYT) China Office, c/o CAAS, 12 Zhongguancun South Street, Beijing, 100081 China; 4 Sino-Russia Agricultural Scientific and Technological Cooperation Center, Heilongjiang Academy of Agricultural Sciences, 368 Xuefu Street, Harbin, 150086 Heilongjiang China; 5 School of Chemical Science and Engineering, Royal Institute of Technology, Teknikringen 42, SE-100 44 Stockholm, Sweden; 6 Institute of Cotton Research, Chinese Academy of Agricultural Sciences (CAAS), 38 Huanghe Street, Anyang, 455000 Henan China; 7 Crop Research Institute, Anhui Academy of Agricultural Sciences, 40 Nongke South Street, Hefei, 230001 Anhui China

**Keywords:** GWAS, Marker-assisted selection, Single nucleotide polymorphism, *Triticum aestivum*

## Abstract

**Background:**

Identification of loci for grain yield (GY) and related traits, and dissection of the genetic architecture are important for yield improvement through marker-assisted selection (MAS). Two genome-wide association study (GWAS) methods were used on a diverse panel of 166 elite wheat varieties from the Yellow and Huai River Valleys Wheat Zone (YHRVWD) of China to detect stable loci and analyze relationships among GY and related traits.

**Results:**

A total of 326,570 single nucleotide polymorphism (SNP) markers from the wheat 90 K and 660 K SNP arrays were chosen for GWAS of GY and related traits, generating a physical distance of 14,064.8 Mb. One hundred and twenty common loci were detected using SNP-GWAS and Haplotype-GWAS, among which two were potentially functional genes underpinning kernel weight and plant height (PH), eight were at similar locations to the quantitative trait loci (QTL) identified in recombinant inbred line (RIL) populations in a previous study, and 78 were potentially new. Twelve pleiotropic loci were detected on eight chromosomes; among these the interval 714.4–725.8 Mb on chromosome 3A was significantly associated with GY, kernel number per spike (KNS), kernel width (KW), spike dry weight (SDW), PH, uppermost internode length (UIL), and flag leaf length (FLL). GY shared five loci with thousand kernel weight (TKW) and PH, indicating significantly affected by two traits. Compared with the total number of loci for each trait in the diverse panel, the average number of alleles for increasing phenotypic values of GY, TKW, kernel length (KL), KW, and flag leaf width (FLW) were higher, whereas the numbers for PH, UIL and FLL were lower. There were significant additive effects for each trait when favorable alleles were combined. UIL and FLL can be directly used for selecting high-yielding varieties, whereas FLW can be used to select spike number per unit area (SN) and KNS.

**Conclusions:**

The loci and significant SNP markers identified in the present study can be used for pyramiding favorable alleles in developing high-yielding varieties. Our study proved that both GWAS methods and high-density genetic markers are reliable means of identifying loci for GY and related traits, and provided new insight to the genetic architecture of GY.

**Electronic supplementary material:**

The online version of this article (10.1186/s12870-019-1781-3) contains supplementary material, which is available to authorized users.

## Background

Bread wheat is an important crop cultivated on ~ 200 million hectares worldwide, and provides one fifth of the total needs of the global population [1–3]. Grain yield (GY) improvement is one of the most challenging objectives in wheat breeding due to the complex genetic architecture and low heritability. The Yellow and Huai River Valleys Wheat Zone (YHRVWZ) is the major wheat-producing region in China, and yield potential in this region has been improved over recent decades [4–6]. However, wheat production in the region is facing problems of decreasing groundwater and hence reduced irrigation frequency and decreasing growing area in the northern part, and frequent occurrence of Fusarium head blight in the southern part. Moreover, there is a decline in the rate of increase of yield potential in conventional breeding.

GY is a complex trait, significantly associated with spike number per unit area (SN), kernel number per spike (KNS) and thousand-kernel weight (TKW). However, grain shape, spike architecture, plant height (PH), and flag leaf related traits can also affect GY through effects on photosynthetic intensity, grain filling and dry matter translocation [5–8]. These traits have higher heritabilities (*h*^*2*^) than GY and are easier to select in small plots at the early stages of breeding programs. Previous studies showed that increased yield potential in the YHRVWZ was largely associated with increased kernels per square meter, biomass and harvest index, and reduced PH [5, 6]. Those improvements were mainly attributed to the use of dwarfing genes (*Rht1*, *Rht2*, *Rht8* and *Rht24*) and the 1BL.1RS translocation lines [8–13]. However, with the current widespread near-fixation of these genes new variation must be sought. It is now believed that further improvement in yield potential can be achieved only by a detailed understanding of its genetic architecture combined with marker-assisted selection (MAS).

MAS is considered to be a key technique to break through yield bottleneck of conventional breeding for further improvement of yield potential of wheat. The application potential of MAS depends on the number of available genes and tightly linked molecular markers. To date, about 65 genes have been cloned in wheat, among which 40 are associated with GY and related traits [14–17]. For all cloned genes, around 150 functional markers have been converted to kompetitive allele-specific PCR (KASP) formats convenient for high-throughput genotyping [15]. Although there are many reports on quantitative trait loci (QTL) mapping and genome-wide association study (GWAS) of yield and related trait loci [18–23], relatively few outcomes have been applied in selection of wheat lines in actual breeding programs. To enhance the application of MAS, more detailed studies on genetic architecture and identification of related loci for GY should be taken.

Single nucleotide polymorphism (SNP) arrays developed from the transcriptomes of plants and animals [24] providing the most advanced approach in searching for candidate genes for economic traits by QTL mapping or GWAS. The wheat 90 K and 660 K SNP arrays are gradually replacing simple sequence repeat (SSR) markers in genetic studies of yield, quality, disease resistance and stress tolerance [25–28]. In our previous study, 23 new stable QTL and 11 QTL clusters were identified for 12 yield related traits using high-density linkage maps constructed with the wheat 90 K SNP array in three RIL populations [17]. Wheat 50 K and 15 K SNP arrays now available for selecting important traits in wheat programs, include SNP markers derived from the wheat 35 K, 90 K and 660 K arrays, functional markers of cloned genes, and closely linked markers identified by QTL mapping and GWAS. SNP markers are becoming the main tool for genetic studies and breeding of crop species.

Analysis of GWAS data is based on linkage disequilibrium (LD) and provides a much higher resolution capacity to capture insights into the genetic architecture of complex traits than traditional QTL mapping [29]. Unlike QTL mapping, GWAS uses available germplasm as materials and bypasses the time of developing segregating populations. Moreover, QTL mapping by bi-parental populations focuses on specific traits, whereas a wider range of germplasm can be used in GWAS to phenotype many traits with one cycle of genotyping. Genetic variance of traits in crop species may be caused by a single SNP, but is more often attributed to several SNPs within a haplotype block [30]. Therefore, SNP-GWAS and Haplotype-GWAS can be complementary and verifiable in identifying genes controlling complex traits. SNP-GWAS is commonly applied in genetic studies of crop species, whereas Haplotype-GWAS has been mostly used in detecting heterozygous chromosome segments in cross-pollinated crops [31, 32].

The aims of the present study were to: 1) identify stable loci for GY and related traits using both SNP-GWAS and Haplotype-GWAS based on high-density SNP markers, 2) investigate genetic relationships among yield and related traits, and 3) detect available loci for MAS of traits in breeding for high yield.

## Results

### Phenotypic evaluation

There was significant and continuous variation in GY and related traits across the diverse panel (Additional file 1: Table S1; Additional file 2: Figure S1). ANOVA showed highly significant effects (*P* < 0.01) of lines, environments and line × environment interactions on all traits (Additional file 3: Table S2). GY in the panel was moderately heritable (*h*^*2*^ = 0.72), whereas the other 12 traits showed high *h*^*2*^ (> 0.89), indicating that most of the traits were stable and largely determined by genetic factors (Additional file 3: Table S2).

GY showed significant (*P* < 0.01) and positive correlations with TKW and kernel width (KW), but significant and negative correlations with PH, uppermost internode length (UIL) and flag leaf length (FLL) (Additional file 4: Table S3); SN was significantly (*P* < 0.01) and negatively correlated with KNS, TKW, KW, spike dry weight (SDW) and flag leaf width (FLW) (*r* = 0.38 to 0.70); KNS exhibited significant (*P* < 0.01) and positive correlations with spike length (SL), SDW and FLW (*r* = 0.36 to 0.64); TKW was significantly (*P* < 0.01) and positively correlated with kernel length (KL), KW and SDW (*r* = 0.50 to 0.83).

### Marker coverage and genetic diversity

After filtering, 326,570 polymorphic SNPs were employed for GWAS analysis; 10,780 were from the wheat 90 K SNP array and 315,790 came from the wheat 660 K SNP array (Additional file 5: Table S4; Additional file 6: Figure S2a). Among polymorphic SNP markers, 39.7, 49.4 and 10.9% were from the A, B and D genomes, respectively. Chromosome 3B had the most SNP markers (41,439), whereas chromosome 4D possessed the least (2061). The total markers spanned a physical distance of 14,064.8 Mb, with an average marker density of 0.043 Mb per marker. The average genetic diversity and polymorphism information content (PIC) for the whole genome were 0.34 and 0.27, respectively, and the average genetic diversities for A, B and D genomes were 0.34, 0.35 and 0.32, and average PIC were 0.28, 0.28 and 0.26, respectively.

### Haplotype composition and coverage

Among all polymorphic SNP markers, 275,000 were assigned to 31,748 haplotype blocks, and 116,555 haplotypes were generated based on 4-gamete LD analyses (Additional file 6: Figure S2b; Additional file 7: Table S5). The D genome had the least haplotype blocks and haplotypes (3384 and 10,579), followed by the A (12,574 and 46,891) and B (15,790 and 59,085) genomes. Like the SNP marker coverage, chromosomes 3B and 4D had the most and least haplotype blocks, respectively. The average number of SNP markers for one haplotype block was 7.9, and the average length was 74.7 kb. For A, B and D genomes, the average numbers of SNP markers were 8.8, 8.7 and 6.1, and the average lengths were 85.9, 93.6 and 44.7 kb, respectively. Haplotype blocks on chromosomes 3B and 5B harbored the most SNP markers (10.8 and 11.0) with maximum length of 112.7 and 97.9 kb, whereas the haplotype blocks on chromosome 4D had the least SNP markers (4.3) and the minimum length (24.8). The range of haplotype block length was 0.001–200.0 kb.

### Population structure and linkage disequilibrium

As shown in Liu et al. [26], the germplasm consisted of three subgroups; Subgroup I contained 62 varieties mainly from Shandong province and foreign countries; Subgroup II had 54 varieties mainly from Henan, Anhui and Shaanxi provinces; and Subgroup III comprised 50 varieties mainly from Henan. Average LD for the whole genome was 8 Mb, and for A, B and D genomes, 6, 4 and 11 Mb, respectively.

### Genome-wide association studies

Totals of 239 and 248 loci for GY and related traits were identified on all 21 chromosomes using Tassel v5.0 and PLINK, respectively (Additional file 8: Table S6; Additional file 9: Figure S3; Additional file 10: Figure S4). In SNP-GWAS 18, 13, 20, 22, 14, 23, 19, 10, 21, 28, 20, 16 and 15 loci were detected for GY, SN, KNS, TKW, KL, KW, SL, SDW, heading date (HD), PH, UIL, FLL and FLW, respectively; in Haplotype-GWAS the corresponding numbers were 20, 13, 9, 27, 13, 32, 11, 13, 21, 27, 27, 24 and 11. In both methods, the D genome possessed the lowest number of loci, consistent with its lowest diversity. One hundred and twenty loci were common in SNP-GWAS and Haplotype-GWAS, 49, 56 and 15 in the A, B and D genomes, respectively (Table 1).

**Table 1:** Loci for grain yield and related traits in the diverse panel identified by both SNP-GWAS and Haplotype-GWAS and comparisons with previous studies

Trait	Chr	Physical position (Mb)^a^	SNP-GWAS			Haplotype-GWAS			QTL/marker/gene^e^
			Marker^b^	Environment	*R*^*2*^ (%)^c^	Haplotype^d^	Environment	*R*^*2*^ (%)^c^	
GY	1A	402.9–407.4	*AX_110387060*	E3/E4/E5/E6/B	6.9–15.5	*H1290*	E4/E5/E6/B	10.1–19.8	
	1A	434.0–439.9	*AX_110418502*	E4/E5/E6/B	6.9–17.7	*H1313*	E4/E5/E6/B	9.3–19.2	*QYld.abrii-1A*_*1*_*.2* [23]
	1B	539.6–542.6	*AX_110508372*	E4/E5/E6/B	7.1–12.8	*H3650*	E4/E5/E6/B	9.3–21.5	
	1B	673.6–675.7	*AX_109820171*	E3/E5/E6/B	7.6–14.3	*H4268*	E4/E5/E6/B	10.7–22.6	
	2A	30.3–31.9	*AX_94546135*	E4/E6/B	7.1–11.6	*H5187*	E4/E6/B	9.3–17.6	
	2B	106.1–108.9	*AX_111210290*	E2/E4/B	7.0–12.5	*H7877*	E1/E4/E5/E6/B	9.1–20.8	
	2D	643.1–650.8	*AX_109941480*	E4/E5/E6/B	7.0–12.3	*H10738*	E4/E5/E6/B	9.3–14.6	
	3A	721.3–724.0	*AX_111492146*	E5/E6/B	7.1–11.7	*H11976*	E3/E5/E6/B	9.1–18.2	
	3B	20.5–22.0	*AX_109881378*	E4/E5/E6/B	7.4–10.7	*H12363*	E4/E5/E6/B	9.3–17.6	
	3D	570.8–572.8	*AX_95257733*	E3/E4/E6/B	7.1–9.9	*H15950*	E1/E4/E4/E6/B	9.2–16.6	
	5A	570.2–570.3	*AX_110523824*	E3/E4/B	7.8–14.8	*H19931*	E1/E3/E4/E5/E6/B	10.0–20.1	*barc151* [35]
	5B	587.1–588.2	*AX_110995303*	E3/E6/B	7.1–10.2	*H22191*	E1/E2/E3/E5/E6/B	9.4–15.7	
SN	5B	530.8–534.0	*IWB56499*	E1/E3/E4/B	7.1–11.9	*H21909*	E2/E3/E4/B	9.1–12.2	
	5B	696.3–700.9	*AX_109936345*	E2/E4/B	7.1–12.1	*H22717*	E1/E2/E3/E4/B	9.1–15.3	
	6B	605.8–607.1	*IWB51629*	E1/E3/B	7.5–8.1	*H26344*	E1/E2/E3/E4/B	9.5–16.6	
	6B	696.6–697.6	*AX_95260682*	E1/E3/B	7.4–9.3	*H26799*	E1/E2/E3/B	10.5–13.6	
	6D	468.8–471.0	*AX_110652999*	E2/E3/B	7.2–17.1	*H27181*	E1/E2/E3/E4/B	9.5–18.8	
	7D	75.9–76.9	*AX_111490489*	E2/E3/B	7.7–9.0	*H31325*	E1/E2/E3/E4/B	9.8–23.3	
KNS	1A	36.8–42.5	*AX_111579941*	E1/E2/E3/E5/E6/B	7.1–11.9	*H322*	E1/E2/E3/E4/E5/E6/B	9.2–15.8	*QGns.wa -1AS.e1* [43]
	1A	497.5–498.1	*AX_108737858*	E1/E2/E3/E4/E5/E6/B	7.1–12.7	*H1479*	E1/E5/E6/B	9.3–12.5	*QGnu.abrii-1A*_*1*_*.1* [23]
	2A	117.2–117.3	*IWB45503*	E3/E4/E5/E6	7.2–8.6	*H5556*	E3/E4/E5/B	9.5–11.5	*QGps.ccsu-2A.3* [34]; *wmc63* [41]; *gwm122* [42]
	2D	633.2–634.5	*IWB57054*	E1/E2/E3/E4/E5/E6/B	7.4–17.7	*H10675*	E1/E3/E6/B	9.3–15.4	
	3A	36.8–37.2	*AX_110657474*	E1/E2/E3/E5/E6/B	7.4–11.1	*H11017*	E1/E2/E3/E5/E6/B	9.7–14.5	
	3A	719.3–725.8	*AX_108992368*	E2/E3/E4/E5/E6/B	7.1–12.4	*H11953*	E3/E4/E5/E6/B	9.1–15.1	*QGnu.abrii-3A* [23]; *QKNS.caas-3AL* [40]
	5B	416.4–418.0	*AX_109538915*	E1/E4/E5/E6/B	7.1–10.5	*H21421*	E1/E4/E6/B	9.3–14.6	*gwm213* [41]
	5B	489.3–492.9	*AX_109537496*	E1/E2/E3/E4/E6/B	7.4–10.9	*H21713*	E1/E2/E3/E4/E5/E6/B	9.5–14.8	
	7A	49.1–50.0	*AX_89571435*	E2/E3/E4/E5/E6/B	7.2–12.6	*H27643*	E1/E3/E4/E5/E6/B	9.3–13.9	
TKW	1B	658.7–662.5	*AX_111147652*	E1/E2/E4/E6/B	7.1–10.2	*H4181*	E1/E2/E3/E4/E5/E6/B	9.2–15.4	*QTgw.cau-1B*, *QGl.cau-1B.1* [51]
	2A	760.6–760.7	*AX_111579921*	E2/E4/E5/B	7.0–8.5	*H7248*	E1/E2/E3/E4/E5/E6/B	9.3–18.1	
	2B	106.0–107.1	*IWB50438*	E1/E2/E4	6.7–9.1	*H7872*	E1/E2/E3/E4/E5/E6/B	9.3–27.0	
	4B	40.4–44.9	*AX_110713957*	E1/E2/E3/E4/E5/E6/B	7.1–12.7	*H17589*	E1/E2/E3/E4/E5/E6/B	9.4–22.0	*QTgw-4B1* [48]
	4B	159.2–163.0	*AX_109427900*	E1/E2/E6/B	7.1–9.2	*H17841*	E1/E2/E3/E4/E5/E6/B	9.4–25.1	
	4B	670.4–670.5	*IWB47765*	E1/E5/E6/B	6.9–9.2	*H18552*	E1/E2/E3/E4/E5/E6/B	10.2–17.9	
	5A	706.2–708.0	*AX_110958315*	E1/E3/E4/E6/B	8.2–13.0	*H20367*	E1/E2/E3/E4/E5/E6/B	9.2–19.8	*QTKW.caas-5AL.1* [40]
	5B	692.7–696.4	*AX_108769612*	E3/E4/E5/E6/B	7.2–9.9	*H22684*	E1/E2/E3/E4/E5/E6/B	9.3–15.8	*Excalibur_c23801_115* [27]; *wsnp_Ex_c16045_24471413* [45]; *Ku_c7546_861* [46]
	6B	450.6–454.1	*AX_110368497*	E2/E3/E5/E6/B	6.8–8.3	*H25900*	E1/E2/E3/E4/E6/B	12.2–14.1	
	6B	675.4–677.5	*AX_109917592*	E1/E3/E6/B	7.2–9.7	*H26662*	E1/E3/E4/E5/E6/B	15.8–20.6	*QTKW.caas-6BL* [17]
	6D	461.4–468.0	*AX_109481324*	E2/E3/E6	6.8–8.3	*H27181*	E1/E2/E3/E4/E5/E6/B	9.3–16.5	
	7D	63.0–69.7	*AX_109927697*	E2/E3/E4/E5/E6/B	7.1–12.3	*H31315*	E1/E2/E3/E4/E5/E6/B	10.4–17.8	*QKL.caas-7DS* [17] *QGw.ccsu-7D.1* [44]
KL	1B	26.9–30.8	*AX_110032293*	E3/E4/E5/E6/B	7.1–12.7	*H2345*	E2/E3/E4/E5/E6/B	9.2–13.5	
	1B	642.6–642.7	*AX_108849700*	E1/E2/E3/E5/E6/B	7.0–9.6	*H4081*	E2/E5/E6/B	9.2–12.0	*QKL.caas-1BL* [17]; *tplb0043a07_1411* [45]
	2A	740.4–745.0	*IWB32119*	E1/E2/E3/E4/E5/E6/B	7.2–14.2	*H7042*	E1/E2/E3/E4/E5/E6/B	9.2–13.8	*TaFlo2-A1* [49]
	3B	782.9–783.0	*AX_111116403*	E1/E2/E5/E6/B	7.2–9.9	*H15574*	E2/E5/E6/B	9.3–11.9	
	5B	52.9–55.2	*IWB50649*	E1/E2/E3/E4/E5/E6/B	7.2–11.7	*H20632*	E3/E5/E6/B	10.5–12.6	*QTgw.abrii-5B*_*1*_*.1* [23]; *QTgw.cau-5B, QGl.cau-5B.2* [47]
	5D	475.8–476.3	*AX_111122970*	E1/E2/E3/E4/E6/B	7.2–12.4	*H22993*	E1/E2/E3/E4/E5/E6/B	9.4–15.4	
KW	1A	9.6–12.0	*IWB6999*	E2/E3/E4/B	7.1–9.3	*H46*	E1/E2/E3/E4/E5/E6/B	9.1–17.5	*QGw.ccsu-1A.1* [44]
	1A	532.6–533.4	*IWB7676*	E2/E3/E4/E6/B	7.0–10.4	*H1679*	E1/E2/E3/E4/E5/E6/B	9.2–16.3	*QTgw.cau-1A* [47]
	2A	27.3–29.9	*AX_111037158*	E1/E3/E4/E5/E6	7.2–9.6	*H5176*	E1/E2/E3/E4/E5/E6/B	9.4–20.2	*wmc177* [42]
	2A	758.6–760.7	*AX_111579921*	E2/E4/E5/E6	7.1–10.9	*H7248*	E2/E3/E4/E5/E6/B	9.2–22.4	
	2B	105.8–108.7	*AX_111634754*	E1/E2/E3/E4/E5/E6/B	7.0–15.0	*H7877*	E1/E2/E3/E4/E5/E6/B	9.1–36.7	
	2B	415.0–418.9	*AX_111819405*	E2/E3/E4/E5/E6/B	7.0–10.6	*H8418*	E1/E2/E3/E4/E5/E6/B	9.4–16.3	
	3A	714.4–716.3	*AX_111047166*	E2/E3/E4/E5/B	7.0–10.9	*H11918*	E1/E2/E3/E4/E5/E6/B	9.1–27.2	*QTgw-3A1* [48]
	3D	570.2–575.5	*IWB17930*	E2/E3/E4/E5/E6/B	6.8–10.3	*H15950*	E1/E2/E3/E4/E5/E6/B	9.2–21.4	
	4A	670.0–676.0	*AX_110046841*	E2/E3/E4/E6/B	7.0–10.3	*H17106*	E1/E2/E3/E4/E6/B	12.1–19.1	*QTKW.caas-4AL* [40]; *QTgw.cau-4A.2*, *QGl.cau-4A.2* [47]
	4B	670.4–672.9	*IWB47765*	E1/E3/E6/B	7.0–12.4	*H18552*	E2/E3/E4/E5/E6/B	11.5–18.8	
	5A	569.2–573.0	*AX_110523824*	E3/E4/E6/B	7.0–11.7	*H19921*	E2/E3/E4/E6/B	9.2–20.9	*QGl.cau-5A.1* [47]
	5A	702.1–708.1	*AX_110958315*	E3/E4/E5/E6/B	7.4–13.3	*H20333*	E2/E3/E4/E5/E6/B	9.4–22.2	*QTKW.caas-5AL.1* [40]
	5B	520.8–520.9	*IWB20926*	E1/E2/E3/E6/B	7.2–10.1	*H21840*	E1/E2/E3/E4/E5/E6/B	9.2–19.4	*QTgw.abrii-5B*_*1*_*.2* [23]
	6B	706.9–709.2	*AX_109820966*	E3/E4/E5/E6/B	6.9–10.3	*H26817*	E1/E2/E3/E4/E5/E6/B	9.4–17.9	
	7D	58.5–66.5	*AX_109396082*	E1/E2/E3/E4/E5/E6/B	7.0–12.5	*H31312*	E3/E4/E6/B	9.5–20.0	*QKL.caas-7DS* [17] *QGw.ccsu-7D.1* [44]
SL	2B	44.6–47.3	*AX_109985540*	E1/E2/E3/E4/E5/B	7.0–14.3	*H7643*	E1/E3/E4/E5/B	9.2–14.0	*BS00022060_51* [27]
	5A	510.1–510.3	*AX_109367907*	E1/E3/E4/E5/E6/B	7.4–12.8	*H19589*	E4/E5/B	9.0–11.8	*QSL.caas-5AL.2* [17]
	5A	534.9–540.1	*AX_110919697*	E1/E2/E3/E4/E5/E6/B	6.7–11.8	*H19737*	E1/E2/E3/E4/B	9.3–14.9	
	5A	568.3–574.3	*AX_110523824*	E1/E2/E3/E4/E5/E6/B	7.3–13.0	*H19954*	E2/E5/E6/B	9.8–10.3	*QSl-5A1* [48]
	5B	398.2–398.3	*AX_94562344*	E3/E4/E5/E6/B	8.0–11.0	*H21291*	E2/E3/E5/E6/B	9.2–15.1	
	5B	590.5–591.1	*AX_110971192*	E1/E3/E4/E6/B	7.4–10.3	*H22208*	E1/E3/E4/E6/B	9.1–12.6	
	7B	723.9–727.6	*IWB71567*	E1/E2/E3/E4/E5/E6/B	7.1–13.8	*H30972*	E1/E2/E3/E4/E6/B	9.1–19.3	
	7D	621.9–630.4	*AX_110645784*	E1/E2/E3/E4/E5/E6/B	7.6–15.0	*H31712*	E1/E2/B	10.7–20.0	
SDW	1A	568.5–573.5	*AX_95255804*	E3/E4/E5/E6/B	7.2–9.7	*H2018*	E3/E4/E6/B	9.1–12.6	
	3A	721.3–725.8	*IWA94*	E3/E4/E5/E6/B	7.1–19.0	*H11992*	E3/E4/E5/E6/B	9.5–15.2	
	4B	89.5–95.1	*IWB35533*	E3/E4/E6/B	7.1–10.7	*H17675*	E4/E5/B	9.3–11.4	
	5B	473.9–477.8	*AX_111183518*	E3/E5/E6/B	7.0–16.3	*H21628*	E3/E4/E5/E6/B	9.7–18.5	
	5B	698.0–698.5	*AX_111051286*	E3/E4/E6/B	7.1–10.6	*H22718*	E3/E4/B	9.9–11.2	
HD	2A	27.3–27.4	*AX_111037158*	E1/E2/E3/E4/E5/B	7.5–12.2	*H5170*	E1/E2/E3/E4/E5/E6/B	11.3–15.7	
	2A	704.8–710.1	*AX_89674107*	E1/E2/E3/E4/E6/B	6.8–8.6	*H6769*	E1/E2/E6	9.3–13.9	
	2A	755.8–757.0	*AX_109964711*	E1/E2/E3/E4/E6/B	6.8–9.4	*H7248*	E1/E2/E4/E6	9.2–13.8	*wPt-1499* [51]
	2B	106.0–108.7	*AX_110624209*	E1/E2/E3/E4/E5/E6/B	6.6–10.7	*H7877*	E1/E2/E4/E6/B	9.4–22.0	
	5B	520.1–524.4	*IWB20926*	E1/E2/E4/E6/B	6.6–10.7	*H21844*	E1/E2/E4/E6	9.1–15.9	*wPt-1409* [51]
	7A	511.5–517.4	*AX_109921812*	E1/E2/E3/E4/E5/B	6.9–9.4	*H28667*	E1/E3/E5/B	9.2–14.2	
	7A	556.2–561.0	*AX_111660137*	E1/E2/E3/E4/E5/B	6.7–12.3	*H28779*	E1/E2/E4/E5/E6	9.4–12.9	*wPt-4796* [51]
	7B	701.0–703.7	*IWB75191*	E1/E2/E3/E4/E5/E6/B	6.6–13.1	*H30856*	E1/E2/E3/E4/E5/E6/B	9.3–16.3	
PH	1A	434.1–440.2	*AX_109449226*	E1/E2/E3/E4/E5/E6/B	6.9–13.1	*H1306*	E1/E2/E3/E4/E5/E6/B	9.3–16.1	
	1B	539.6–542.6	*AX_94564150*	E1/E2/E4/E5/E6/B	7.0–21.1	*H3651*	E1/E2/E3/E4/E5/E6/B	10.4–22.8	
	1B	673.9–675.7	*AX_109820171*	E1/E2/E4/E5/E6/B	6.8–30.8	*H4268*	E1/E2/E3/E4/E5/E6/B	9.5–38.0	
	2A	30.9–32.0	*AX_110988136*	E1/E2/E4/E5/B	7.0–11.3	*H5187*	E1/E2/E3/E4/E5/B	11.4–19.8	*QUIL.caas-2AS.1* [17]
	2A	715.3–721.6	*AX_94494373*	E1/E2/E4/E6/B	6.7–10.5	*H6911*	E1/E2/E3/E4/E5/E6/B	10.9–16.5	*QPH.caas-2AL*, *QUIL.caas-2AL* [17]
	3A	716.5–721.3	*AX_111577195*	E1/E2/E4/E5/E6/B	6.9–14.8	*H11943*	E1/E2/E3/E4/E5/E6/B	9.2–18.4	*barc1113* [19]
	3B	116.1–120.4	*AX_109413472*	E1/E2/E3/E4/E5/E6/B	6.8–12.8	*H12783*	E1/E2/E3/E4/E5/E6/B	9.0–17.4	*gwm566* [19]
	4D	16.6–19.7	*AX_108916749*	E1/E2/E4/E5/E6/B	6.7–14.5	*H18598*	E1/E2/E3/E4/E5/E6/B	10.2–16.1	*QPH.caas-4DS*, *QUIL.caas-4DS* [17]; *Rht-D1b* [52]
	5A	669.2–671.2	*AX_110446653*	E1/E2/E3/E4/E5/E6/B	6.9–14.7	*H20207*	E1/E2/E3/E4/E5/E6/B	10.8–17.5	
	5A	708.4–708.8	*IWA2646*	E2/E3/E5/B	6.7–8.0	*H20356*	E1/E2/E3/E4/E5/E6/B	9.4–18.0	*QPh.hwwgr5AL* [53]
	5B	576.3–580.9	*AX_108921249*	E1/E2/E4/E5/B	7.0–13.3	*H22142*	E1/E2/E3/E4/E5/E6/B	12.2–20.4	
	6B	1.4–4.9	*AX_110482029*	E1/E2/E4/E5/B	7.0–18.1	*H24673*	E1/E2/E3/E4/E5/E6/B	9.4–25.9	
	6B	42.7–42.8	*AX_110671479*	E1/E2/E4/B	7.2–10.8	*H24862*	E1/E2/E3/E4/E5/E6/B	9.4–19.0	
	7A	556.2–560.9	*AX_109384874*	E1/E2/E3/E5/E6/B	6.9–15.1	*H28784*	E1/E2/E3/E4/E5/E6/B	10.5–18.6	
UIL	1A	142.6–148.6	*AX_109901254*	E1/E5/E6/B	7.5–11.1	*H608*	E1/E2/E5/E6/B	9.2–17.4	
	1A	434.4–440.2	*AX_109449226*	E1/E2/E5/E6/B	6.9–12.9	*H1306*	E1/E2/E5/E6/B	9.1–16.3	
	1B	539.6–542.6	*AX_94564150*	E1/E2/E5/B	6.8–16.1	*H3650*	E1/E2/E5/B	9.6–17.9	
	1B	674.8–675.7	*AX_109820171*	E1/E2/E5/B	8.4–16.4	*H4239*	E1/E2/E5/E6/B	9.2–24.0	
	3A	721.3–723.7	*AX_111610555*	E1/E2/E5/B	7.3–9.7	*H11922*	E1/E2/E5/E6/B	9.1–17.3	*barc1113* [19]
	5A	665.6–671.2	*IWA5929*	E1/E2/E5/E6/B	7.1–11.3	*H20205*	E1/E2/E5/E6/B	9.6–18.5	
	6B	1.4–4.9	*AX_109526332*	E1/E2/E5/B	7.0–11.4	*H24673*	E1/E2/E5/E6/B	9.3–24.8	
	6B	42.7–46.9	*IWB12568*	E1/E2/E5/E6/B	7.1–9.5	*H24865*	E1/E2/E5/E6/B	9.8–20.4	
	6B	563.3–567.8	*AX_86165710*	E1/E2/E5/E6/B	6.7–10.5	*H26273*	E1/E2/E5/E6/B	9.3–15.6	
	6D	396.5–403.7	*AX_109331000*	E1/E2/E5/E6/B	7.0–12.6	*H27113*	E1/E2/E5/E6/B	15.3–19.0	*barc96* [19]
	6D	460.7–465.0	*IWB2743*	E1/E2/E5	7.2–10.5	*H27272*	E1/E2/E5/E6/B	9.4–14.1	
	7B	630.6–632.7	*AX_109492373*	E1/E2/E5/B	7.1–12.3	*H30631*	E1/E2/E5/E6/B	9.9–22.1	
FLL	1A	8.3–11.0	*AX_109621606*	E3/E4/E5/E6/B	7.1–16.9	*H99*	E3/E4/E5/E6/B	9.0–20.8	
	2A	2.5–7.8	*AX_109880304*	E2/E3/E5/E6/B	7.4–19.6	*H5121*	E2/E3/E4/E5/E6/B	10.9–29.1	
	2A	87.8–87.9	*AX_95085564*	E4/E5/E6/B	8.6–12.7	*H5461*	E2/E3/E5/E6/B	9.6–17.5	
	2B	46.4–50.4	*AX_111027654*	E2/E6/B	7.0–19.6	*H7632*	E2/E3/E4/E5/E6/B	9.4–25.5	*QFLL-2B* [57]
	3A	716.9–722.5	*AX_108908243*	E3/E4/E6	7.2–9.7	*H11976*	E3/E4/E5/E6/B	9.4–22.7	
	5A	556.6–559.7	*IWB4576*	E3/E4/E6/B	6.9–9.6	*H19845*	E2/E3/E4/E5/E6/B	9.6–16.2	*QFlw.cau-5A.2*, *QFlan.cau-5A.3* [56]
	6B	704.9–708.7	*AX_109459603*	E5/E6/B	8.8–9.6	*H26801*	E2/E3/E4/E5/E6/B	9.5–22.0	
	6D	455.5–462.2	*AX_110876641*	E3/E6/B	7.6–8.8	*H27138*	E2/E3/E4/E5/E6/B	9.8–18.0	*QFll.cau-6D* [56]
FLW	1A	440.4–445.5	*AX_111540798*	E2/E3/E4/E6/B	7.0–11.4	*H1318*	E2/E3/E4/B	9.1–17.3	
	3B	23.9–24.4	*AX_111655083*	E4/E6/B	7.3–9.7	*H12389*	E2/E3/E4/E5/E6/B	9.2–19.7	
	5B	531.5–533.5	*IWB41225*	E2/E6/B	6.9–7.9	*H21876*	E2/E3/E4/E5/E6/B	9.1–14.7	
	5B	557.1–559.4	*AX_109519234*	E2/E3/E4/E5/B	7.0–9.0	*H22030*	E2/E3/E4/E5/E6/B	9.3–16.7	
	6B	461.4–465.5	*AX_108771909*	E2/E3/E5/B	7.0–9.9	*H25955*	E2/E3/E4/E5/E6/B	9.6–12.2	*QFlw.cau-6B* [56]

^a^ The physical positions of SNP markers based on wheat (Chinese Spring) genome sequences from the International Wheat Genome Sequencing Consortium

^b^ Representative markers

^c^ Percentage of phenotypic variance explained

^d^ Representative haplotypes

^e^ The previously reported QTL, markers or genes near the loci identified in the present study

*GY* grain yield, *SN* spike number per square meter, *KNS* kernel number per spike, *TKW* thousand-kernel weight, *KL* kernel length, *KW* kernel width, *SL* spike length, *SDW* spike dry weight, *HD* heading date, *PH* plant height, *UIL* uppermost internode length, *FLL* flag leaf length, *FLW* flag leaf width; E1: 2012–2013 Anyang; E2: 2012–2013 Suixi; E3: 2013–2014 Anyang; E4: 2013–2014 Suixi; E5: 2014–2015 Anyang; E6: 2014–2015 Shijiazhuang; B: Best linear unbiased estimation

### GY and yield components

Twelve common loci for GY were identified on chromosomes 1A (2), 1B (2), 2A, 2B, 2D, 3A, 3B, 3D, 5A and 5B, with single loci explaining 6.9–17.7% and 9.1–22.6% of the phenotypic variances in SNP-GWAS and Haplotype-GWAS, respectively. Seven loci, on 1A (*AX_110387060* and *AX_110418502*), 1B (*AX_110508372* and *AX_109820171*), 2D (*AX_109941480*), 3B (*AX_109881378*), and 3D (*AX_95257733*) were detected in four environments and best linear unbiased estimation (BLUE) values by both methods. The 1A (*AX_110418502*) and 1B (*H4268*) loci explained the largest of phenotypic variances in SNP-GWAS and Haplotype-GWAS, respectively.

Six common loci for SN were detected on chromosomes 5B (2), 6B (2), 6D and 7D, explaining 7.1–17.1% and 9.1–23.3% of the phenotypic variances in SNP-GWAS and Haplotype-GWAS, respectively. The 5B locus (*IWB56499*) was significant in three environments and BLUE value, whereas the 6D locus (*AX_110652999*) explained the largest phenotypic variance (7.2–17.1%) in SNP-GWAS. Loci on chromosomes 5B (*H22717*), 6B (*H26344*), 6D (*H27181*) and 7D (*H31325*) were identified in four environments and BLUE values, among which the 7D locus (*H31325*) accounted for the largest of phenotypic variance (9.8–23.3%) in Haplotype-GWAS.

Nine common loci for KNS were found on chromosomes 1A (2), 2A, 2D, 3A (2), 5B (2) and 7A, accounting for 7.1–17.1% and 9.1–15.8% of the phenotypic variances in SNP-GWAS and Haplotype-GWAS, respectively. In SNP-GWAS, the loci on chromosomes 1A (*AX_108737858*) and 2D (*IWB57054*) were identified in all six environments and BLUE values; the 2D locus (*IWB57054*) accounted for the largest phenotypic variance (7.4–17.7%). In Haplotype-GWAS, the loci on chromosomes 1A (*H322*) and 5B (*H21713*) were significant in all six environments and BLUE values; the 1A locus (*H322*) explained the largest phenotypic variance (9.2–15.8%). Four loci, including 1A (*AX_111579941*), 3A (*AX_110657474*), 5B (*AX_109537496*) and 7A (*AX_89571435*), were identified in five or more environments and BLUE values in both methods and were therefore stable.

Twelve common loci for TKW were identified on chromosomes 1B, 2A, 2B, 4B (3), 5A, 5B, 6B (2), 6D and 7D, explaining 6.7–13.0% and 9.2–27.0% of the phenotypic variances in SNP-GWAS and Haplotype-GWAS, respectively. In SNP-GWAS, the 4B locus (*AX_110713957*) and 7D locus (*AX_109927697*) were significant in at least five environments and BLUE values; in Haplotype-GWAS, all the loci were significant in at least five environments and BLUE values. The 5A (*AX_110958315*) and 2B (*H7872*) loci explained the largest phenotypic variances in SNP-GWAS and Haplotype-GWAS, respectively.

### Kernel shape related traits

Six common loci for KL on chromosomes 1B (2), 2A, 3B, 5B and 5D explained 7.0–14.2% and 9.2–15.4% of the phenotypic variances in SNP-GWAS and Haplotype-GWAS, respectively. The 2A locus (*IWB32119*) was significant in all six environments and BLUE value, whereas the 5D locus (*AX_111122970*) was identified in five or six environments and BLUE value by both methods.

Fifteen common loci for KW on chromosomes 1A (2), 2A (2), 2B (2), 3A, 3D, 4A, 4B, 5A (2), 5B, 6B and 7D accounted for 6.8–15.0% and 9.1–36.7% of the phenotypic variances in SNP-GWAS and Haplotype-GWAS, respectively. The 2B locus (*AX_111634754*) was significant in all six environments and BLUE value with the largest contribution to phenotypic variance in both methods. The 2B (*AX_111819405*) and 3D (*IWB17930*) loci were significant in five environments and BLUE values in SNP-GWAS, and significant in all six environments and BLUE values in Haplotype-GWAS.

### Spike related traits

Eight common loci for SL were identified on chromosomes 2B, 5A (3), 5B (2), 7B and 7D, explaining 6.7–15.0% and 9.0–20.0% of the phenotypic variances in SNP-GWAS and Haplotype-GWAS, respectively. Locus (*IWB71567*) on chromosome 7B was significant in five or more environments and BLUE value in both methods; the 7D locus (*AX_110645784*) explained 7.6–15.0% and 10.7–20.0% of the phenotypic variances in SNP-GWAS and Haplotype-GWAS, respectively.

Five common loci for SDW detected on chromosomes 1A, 3A, 4B, and 5B (2) explained 7.0–19.0% and 9.1–18.5% of the phenotypic variances in SNP-GWAS and Haplotype-GWAS, respectively. The 3A locus (*IWA94*) was significant in all four environments and BLUE value in both methods; the 5B locus (*AX_111183518*) was stable across three or four environments and BLUE value in both methods.

### Heading date

Eight common loci for HD on chromosomes 2A (3), 2B, 5B, 7A (2) and 7B accounted for 6.6–13.1% and 9.1–22.0% of the phenotypic variances in SNP-GWAS and Haplotype-GWAS, respectively. The locus (*IWB75191*) on chromosome 7B was significant in all six environments and BLUE value in both methods, whereas locus (*AX_111037158*) on chromosome 2A was stably detected in five and six environments and BLUE value in SNP-GWAS and Haplotype-GWAS, respectively.

### Plant height related traits

Fourteen common loci for PH were identified on chromosomes 1A, 1B (2), 2A (2), 3A, 3B, 4D, 5A (2), 5B, 6B (2) and 7A, explaining 6.7–30.8% and 9.0–38.0% of the phenotypic variances in SNP-GWAS and Haplotype-GWAS, respectively. The loci on chromosomes 1A (*AX_109449226*), 3B (*AX_109413472*) and 5A (*AX_110446653*) were significant in all six environments and BLUE values, whereas the other five loci on chromosomes 1B (*AX_94564150* and *AX_109820171*), 3A (*AX_111577195*), 4D (*AX_108916749*) and 7A (*AX_109384874*) were stably identified in five or six environments and BLUE values in both methods; the 1B (*AX_109820171*) locus was the most significant, explaining 6.8–30.8% and 9.5–38.0% of the phenotypic variances in SNP-GWAS and Haplotype-GWAS, respectively.

Twelve common loci for UIL were detected on chromosomes 1A (2), 1B (2), 3A, 5A, 6B (3), 6D (2) and 7B, with single loci explaining 6.7–16.4% and 9.1–24.8% of the phenotypic variances in SNP-GWAS and Haplotype-GWAS, respectively. Five loci on chromosomes 1A (*AX_109449226*), 5A (*IWA5929*), 6B (*IWB12568* and *AX_86165710*) and 6D (*AX_109331000*) were identified in all four investigated environments and BLUE values by the two methods. Locus (*AX_109820171*) on chromosome 1B had a large effect on phenotypic variance in both methods.

### Flag leaf related traits

Eight common loci for FLL on chromosomes 1A, 2A (2), 2B, 3A, 5A, 6B and 6D explained 6.9–19.6% and 9.0–29.1% of the phenotypic variances in SNP-GWAS and Haplotype-GWAS, respectively. The 2A locus (*AX_109880304*) was significant in four or five environments and BLUE value and presented the largest effect on phenotypic variance in both methods. The 1A locus (*AX_109621606*) was also detected in four environments and BLUE value in both methods.

Five common loci for FLW were identified on chromosomes 1A, 3B, 5B (2) and 6B, accounting for 6.9–11.4% and 9.1–19.7% of the phenotypic variances in SNP-GWAS and Haplotype-GWAS, respectively. The locus on chromosome 5B (*AX_109519234*) was significant in four or five environments and BLUE value in both methods, whereas 1A (*AX_111540798*) and 3B (*AX_111655083*) loci explained the highest phenotypic variances in SNP-GWAS and Haplotype-GWAS, respectively.

### Pleiotropic loci

Twelve pleiotropic loci were associated with three or more traits on chromosomes 1A, 1B (2), 2A (2), 2B, 3A, 5A (2), 5B (2) and 6D based on the common loci detected by both methods (Table 2). The interval 714.4–725.8 Mb on chromosome 3A was associated with GY, KNS, KW, SDW, PH, UIL and FLL, showing a significant effect on GY. Seven pleiotropic loci were associated with GY, among which four were related to KW and five to PH or UIL. Three SN loci on chromosomes 5B (*IWB56499* and *AX_109936345*) and 6D (*AX_110652999*) were located in pleiotropic loci; four HD loci on chromosomes 2A (*AX_111037158* and *AX_111579921*), 2B (*AX_111634754*) and 5B (*IWB56499*) were also located in pleiotropic loci; these loci were both accompanied with TKW or KW loci. Finally, nine pleiotropic loci for TKW or KW and seven loci for PH or UIL should be crucial in determining GY. Of all common loci identified by both methods, more than half were co-localized.

**Table 2:** Distribution of pleiotropic loci associated with three or more grain yield related traits on wheat chromosomes

Chr	Trait	Marker^a^	Interval (Mb)^b^
1A	GY/PH/UIL/FLW	*AX_110418502*	434.0–445.5
1B	GY/PH/UIL	*AX_94564150*	539.6–542.6
1B	GY/PH/UIL	*AX_109820171*	673.6–675.7
2A	GY/KW/HD/PH	*AX_111037158*	27.3–32.0
2A	TKW/KW/HD	*AX_111579921*	755.8–760.7
2B	GY/TKW/KW/HD	*AX_111634754*	105.8–108.9
3A	GY/KNS/KW/SDW/PH/UIL/FLL	*IWA94*	714.4–725.8
5A	GY/KW/SL	*AX_110523824*	568.3–574.8
5A	TKW/KW/PH	*AX_110958315*	702.1–708.8
5B	SN/KW/HD/FLW	*IWB56499*	520.1–534.0
5B	SN/TKW/SDW	*AX_109936345*	692.7–700.9
6D	SN/TKW/UIL/FLL	*AX_110652999*	455.5–471.0

^a^ Representative markers

^b^ The physical positions of SNP markers based on wheat (Chinese Spring) genome sequences from the International Wheat Genome Sequencing Consortium

*GY* grain yield, *SN* spike number per square meter, *KNS* kernel number per spike, *TKW* thousand-kernel weight, *KL* kernel length, *KW* kernel width, *SL* spike length, *SDW* spike dry weight, *HD* heading date, *PH* plant height, *UIL* uppermost internode length, *FLL* flag leaf length, *FLW* flag leaf width

### Relationships between trait performances and number of alleles for increasing phenotypic values

For most traits, ranges in the number of alleles for increasing phenotypic values across the panel were large (Table 3). The average number of alleles for increasing GY was 10.0. Compared with the higher numbers of alleles for increasing TKW, KL, KW and FLW, those for SN, KNS, SL, SDW, HD, PH, UIL and FLL were lower.

**Table 3:** Number of alleles for increasing phenotypic values of grain yield and related traits in the diverse panel

Trait	Total number of favorable alleles	Average number of favorable alleles	Range
GY	12	10.0	3–12
SN	6	1.7	0–5
KNS	9	3.7	0–7
TKW	12	8.5	2–12
KL	6	3.6	1–6
KW	15	11.6	7–14
SL	8	1.3	0–7
SDW	5	2.0	0–5
HD	8	3.5	0–8
PH	14	1.3	2–7
UIL	12	3.4	1–8
FLL	8	2.3	0–6
FLW	5	3.9	0–5

*GY* grain yield, *SN* spike number per square meter, *KNS* kernel number per spike, *TKW* thousand-kernel weight, *KL* kernel length, *KW* kernel width, *SL* spike length, *SDW* spike dry weight, *HD* heading date, *PH* plant height, *UIL* uppermost internode length, *FLL* flag leaf length, *FLW* flag leaf width

**Fig. 1:**
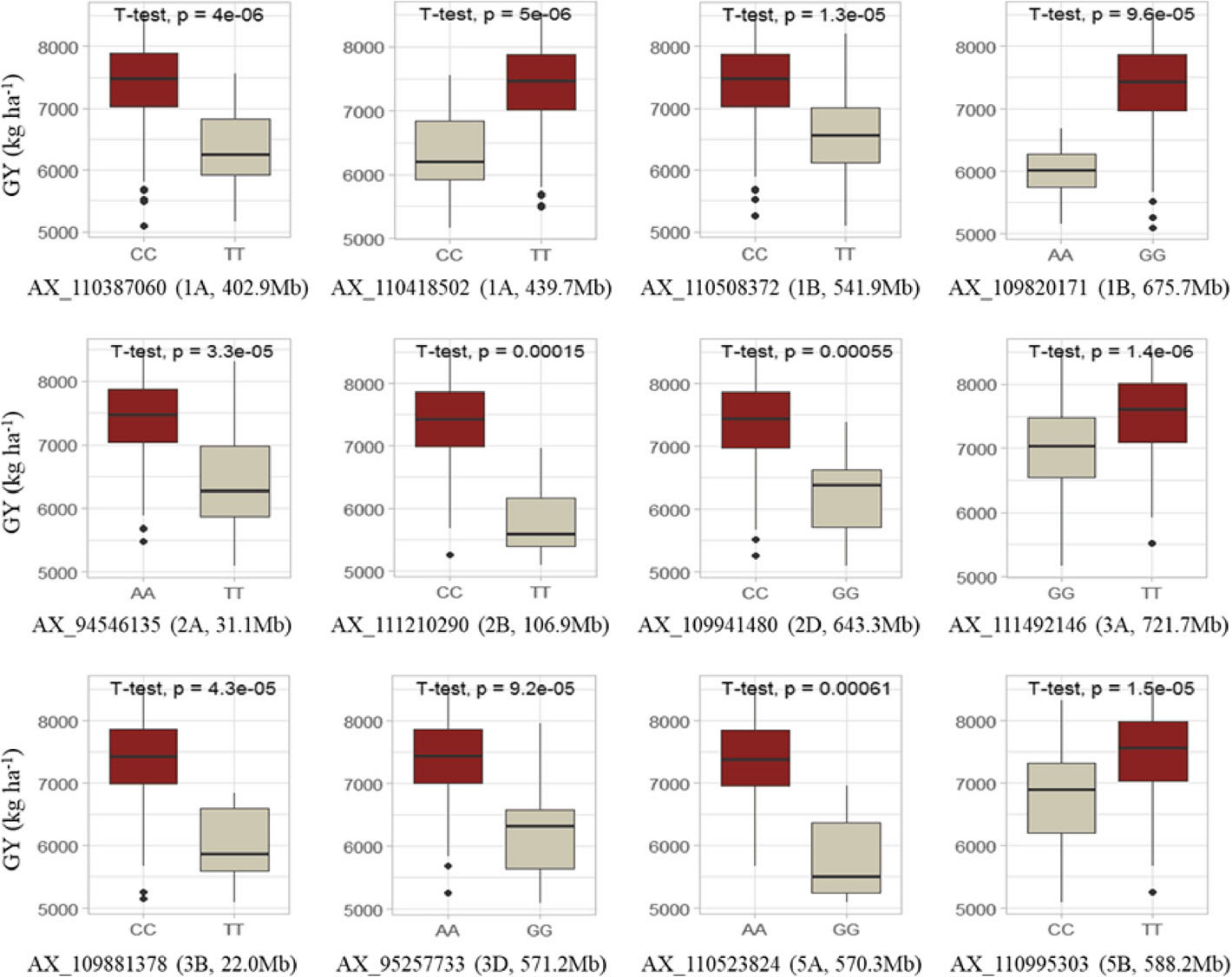
Effects of each locus on the phenotypic values for grain yield

**Fig. 2:**
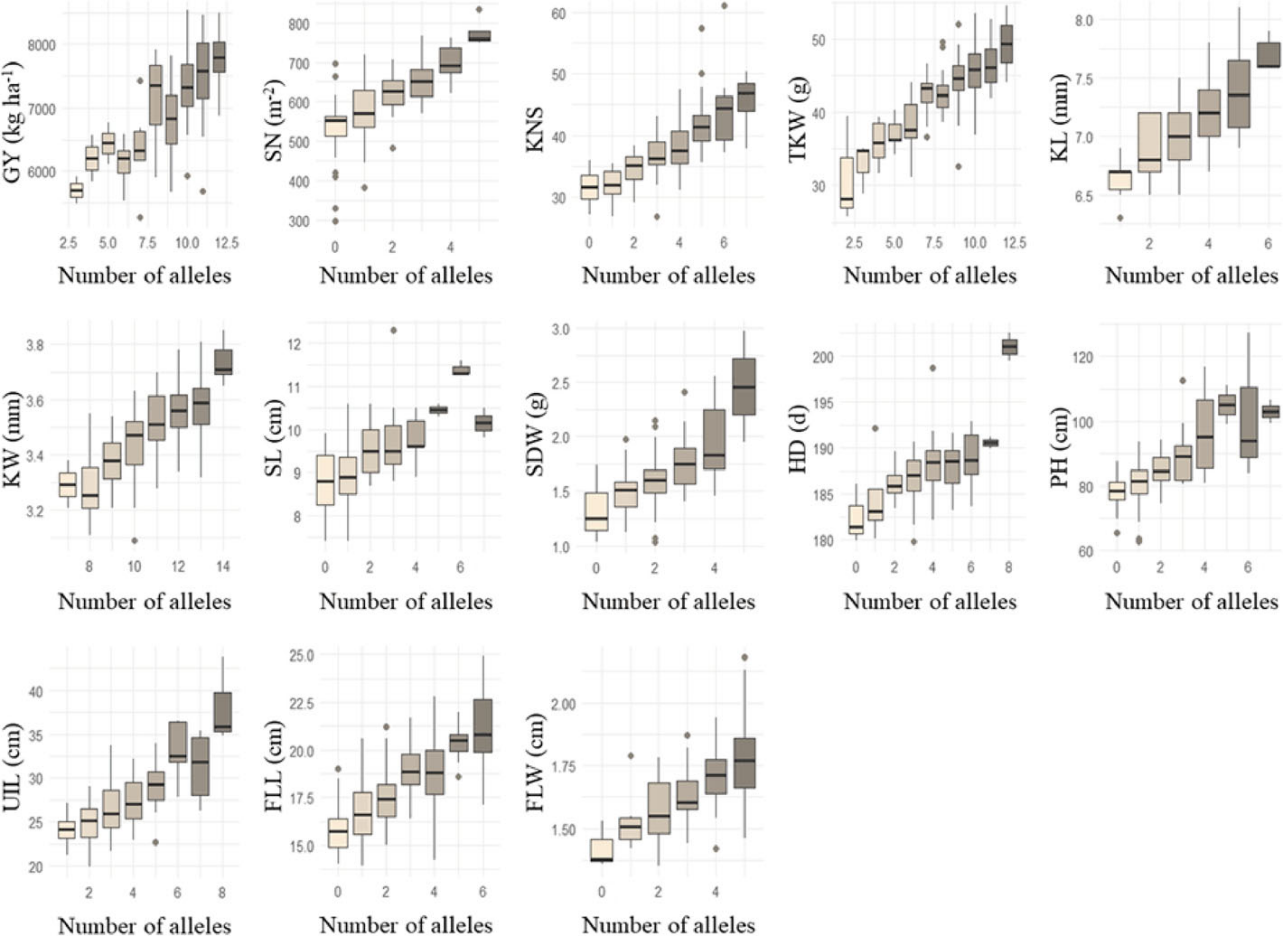
Effects of the number of alleles for increasing phenotypic values of grain yield and related traits. GY, grain yield; SN, spike number per square meter; KNS, kernel number per spike; TKW, thousand-kernel weight; KL, kernel length; KW, kernel width; SL, spike length; SDW, spike dry weight; HD, heading date; PH, plant height; UIL, uppermost internode length; FLL, flag leaf length; FLW, flag leaf width

Favorable alleles at each locus for GY exhibited significant and positive effects on phenotypic values (Fig. 1). Effects of number of alleles for increasing phenotypic values for each trait were also estimated (Fig. 2), and the results showed that the phenotypic traits were dependent on the number of alleles for increasing phenotypic value.

## Discussion

### Advantages of two methods of GWAS using high-density SNP markers

SNP arrays based on Next Generation Sequencing Technology permit identification of many SNP markers, and represent very high throughput and multiple genotyping compared with traditional molecular markers [24]. In differing from QTL mapping, GWAS is performed by significance testing between phenotypic values and single markers or haplotype blocks comprised of contiguous SNP markers with similar genotype. The accuracy of GWAS results thus depends on the coverage of markers used for analysis. In the present study, 326,570 SNP markers from the wheat 90 K and 660 K SNP arrays were used for GWAS of GY and related traits, with a physical distance of 0.043 Mb per marker. The average LD for the whole genome was 8 Mb, and the high-density of SNP markers ensured multiple markers in each haplotype block and high efficiency in identifying significant loci.

SNP are very common in the genomes of most crop species and result in a variety of genetic variances. However, genetic variance in crops can sometimes be caused by single SNP, but mostly there are numerous closely linked SNPs [30]. In order to avoid the disadvantage of SNP-GWAS in detecting genetic multiple variances caused by numerous SNP and false positives identified by Haplotype-GWAS, both methods were used in the present study to identify loci with significant effects. As already mentioned above, 275,000 of a total 326,570 SNP markers were sorted into 31,748 haplotype blocks, remaining 51,570 single SNP markers. A total of 239 and 248 significant loci were detected and about half the loci were common in both methods. This indicated that the detection intensity of SNP-GWAS and Haplotype-GWAS differs between chromosome positions. Loci identified in three or more environments in both methods were regarded as the main loci affecting GY and related traits.

### Comparison with the QTL identified in previous studies

GY and related traits are basic observable and measurable agronomic traits extensively reported in the literature. Being limited to low-density molecular markers, significant influence by environments, and likely presence of linkage drag, marker loci for GY and related traits identified by QTL mapping or GWAS are seldom used in wheat breeding programs. In the present study, associations of GY and related traits with single SNPs and haplotype blocks were conducted separately. Loci identified by both methods were compared with QTL previously reported on physical or linkage maps.

### GY and its components

GY related QTL have been reported on all 21 wheat chromosomes [18, 23, 33–38]. Azadi et al. [23] reported a GY QTL on chromosome 1A tightly linked with SSR marker *gwm357*, which was also located between the two GY QTL by Cuthbert et al. [18] and Huang et al. [33]. The 1A locus (*AX_110418502*) for GY is about 0.21 cM from *gwm357* on the consensus linkage map [39], indicating that these two loci are likely to be the same. Reif et al. [35] identified a GY QTL on chromosome 5A linked with SSR marker *barc151*, at a similar position to the present GY locus (*AX_110523824*). The remaining loci are likely to be new.

Numerous reports indicate that SN is controlled by polygenes and significantly influenced by environment. Nine SN QTL were recently mapped using the wheat 90 K SNP array on three RIL populations [17]. *QSN.caas-3AL.1* and *QSN.caas-6AL* were at similar positions to the QTL reported in Lee et al. [36] and Gao et al. [40], whereas the effect of *QSN.caas-4BS* was contributed by *Rht-B1b*. However, the six SN loci detected in this study are likely to be at different positions to the QTL reported previously.

Azadi et al. [23] detected KNS QTL on chromosomes 1A, 3A and 5B, linked with DArT markers *wPt-665,590*, *wPt-5133* and *wPt-3661*, respectively; the 1A QTL is about 1.5 cM from the KNS locus (*AX_108737858*) identified in this study and they are likely to be the same; the 3A QTL is about 2 cM from the KNS locus *AX_108992368* and close to a QTL mapped by Gao et al. [40]; the 5B QTL is about 6.5 cM from the KNS locus *AX_109537496* and therefore might be different. Zhang et al. [41] identified SSR markers *wmc63* and *gwm213* significantly associated with KNS on chromosomes 2A and 5B, respectively; *wmc63* is about 2 cM from the present 2A locus *IWB45503* and close to a QTL reported by Kumar et al. [34] and Yao et al. [42]; *gwm213* is at the same position as the present KNS locus *AX_109538915*. In addition, the locus *AX_111579941* on chromosome 1A is about one LD from a QTL reported in Wang et al. [43]. The stable loci on chromosomes 3A (*AX_110657474*), 5B (*AX_109537496*) and 7A (*AX_89571435*) identified in five or more environments and BLUE values by both methods are likely to be new.

TKW locus *AX_109917592* on chromosome 6B is within the confidence interval of *QTKW.caas-6BL* detected in the D × S (Doumai × Shi 4185) population in Li et al. [17]. The 7D locus (*AX_109927697*) is at the similar position to *QKL.caas-7DS* located in the G × Z (Gaocheng 8901 × Zhoumai 16) population [17] and *QGw.ccsu-7D.1* reported by Mir et al. [44]. TKW locus *AX_108769612* on chromosome 5B is at the same position as loci for KL, KW and TKW detected by Chen et al. [45], Mohler et al. [46] and Sun et al. [27], respectively, indicating that this should be an important locus in determining kernel weight. Wu et al. [47] reported a locus affecting both TKW and KL on chromosome 1B, located about one LD from the present TKW locus *AX_111147652*. Chromosomes 5A locus *AX_110958315* is about one LD from a TKW QTL reported by Gao et al. [40], whereas 4B locus *AX_110713957* is at a similar position to a QTL reported in Liu et al. [48]. The other six loci are likely to be new.

### Kernel shape related traits

Sajjad et al. [49] cloned *TaFlo2-A1* for TKW on chromosome 2A, at the same position as the present stable KL locus *IWB32119*. 1B locus *AX_108849700* is within the confidence interval of *QKL.caas-1BL* mapped in the L × Z (Linmai 2 × Zhong 892) population in Li et al. [17] and within one LD of the significantly associated SNP marker *tplb0043a07_1411* for TKW [48]. KL locus *IWB50649* on chromosome 5B is very close to QTL reported by Azadi et al. [23] and Wu et al. [47], and within the interval of a TKW QTL mapped by Zhai et al. [50]. Locus *AX_111122970* on chromosome 5D stably detected in five or six environments and BLUE value by both methods is probably new.

The co-localized KW and TKW locus *AX_110958315* is at the same position as a TKW QTL on chromosome 5A reported by Gao et al. [40]. Another KW locus *AX_109396082* co-localized with TKW locus *AX_109927697* on chromosome 7D is at a similar position to QTL reported in Mir et al. [44] and Li et al. [17]. Wu et al. [47] reported three TKW or KL QTL on chromosomes 1A, 4A and 5A, which are within one LD from the KW loci *IWB7676*, *AX_110046841* and *AX_110523824*, respectively; 4A locus *AX_110046841* is also close to a TKW QTL mapped by Gao et al. [40]. The KW loci on chromosomes 1A (*IWB6999*), 2A (*AX_111037158*) and 3A (*AX_111047166*) are about one LD from TKW QTL reported by Mir et al. [44], Yao et al. [42] and Liu et al. [48], respectively. Locus *IWB20926* on chromosome 5B is about 2 cM from DArT marker *wPt-5851* linked to a TKW QTL in Azadi et al. [23]. The stable loci on chromosomes 2B (*AX_111634754* and *AX_111819405*) and 3D (*IWB17930*) identified in five or six environments and BLUE values by both methods are likely to be new.

### Spike related traits

*QSL.caas-5AL.2* identified in the G × Z population [17] is at a similar position to the present SL locus *AX_109367907*. Sun et al. [27] reported SNP marker *BS00022060_51* associated with SL on chromosome 2B. This gene is about one LD from the SL locus *AX_109985540*, indicating they are likely to be the same. Liu et al. [48] mapped a SL QTL on chromosome 5A about one LD from SL locus *AX_110523824* in this study. The stable locus *IWB71567* on chromosome 7B detected in five or six environments and BLUE value by both methods is likely to be new.

Compared with other traits there are few reports on QTL mapping of SDW. Li et al. [17] mapped 10 SDW QTL; among them *QSDW.caas-6BL* and *QSDW.caas-7BL* are at similar positions to SNPs *RAC875_c31299_1302* and *BS00055584_51* identified by Valluru et al. [28]. All five SDW loci identified in this study appear to be new.

### Heading date

Le Gouis et al. [51] reported DArT markers *wPt-1499*, *wPt-1409* and *wPt-4796* associated with HD on chromosomes 2A, 5A and 7A, respectively; these three markers are close to the HD loci *AX_109964711*, *IWB20926* and *AX_111660137*, respectively, on the consensus linkage map [39]. As the majority of varieties in the present study were from the YHRVWZ with similar vernalization and photoperiod characteristics, and no variation associated with known *Vrn* and *Ppd* genes was detected. Stable loci on chromosomes 2A (*AX_111037158*) and 7B (*IWB75191*) detected in most environments and BLUE values by both methods are likely to be new.

### Plant height related traits

*Rht-D1b* is widely present in wheat varieties in YHRVWZ [6]. The PH locus *AX_108916749* on chromosome 4D is at the same position as *Rht-D1* [52], indicating that the effect on PH is from *Rht-D1b*, and is the same as QTL or loci reported by Li et al. [17], Sun et al. [27] and Gao et al. [40]. Loci *AX_110988136* and *AX_94494373* on chromosome 2A are at similar positions to *QUIL.caas-2AS.1* and *QPH.caas-2AL* (co-localized with *QUIL.caas-2AL*), respectively [17]. Cui et al. [19] identified QTL for PH or UIL on chromosomes 3A and 3B; these QTL are close to the present PH loci *AX_111577195* and *AX_109413472*, respectively. 3B locus *AX_109413472* is about 14 cM from *Rht5* [7] and therefore should be different. 5A locus *IWA2646* is about one LD from a QTL in Li et al. [53], and about 25 Mb and 8.9 cM from *Rht12* [54], respectively, on the physical and consensus linkage maps [39]. 5B locus *AX_108921249* is about 2 Mb from *Vrn-B1* [55], but there is no reported relationship between vernalization response and PH. The five loci identified in 1A (*AX_109449226*), 1B (*AX_94564150* and *AX_109820171*), 5A (*AX_110446653*) and 7A (*AX_109384874*) identified in five or more environments and BLUE values by both methods are likely to be new.

The UIL locus *AX_111610555*, co-localized with PH locus *AX_111577195*, is likely to be the same as a QTL on chromosome 3A for both PH and UIL reported by Cui et al. [19]. Another UIL locus (*AX_109331000*) on chromosome 6D is about one LD from a QTL associated with PH and third internode length reported in Cui et al. [19]; they are likely to be the same. Apart from 3A locus, the remaining six loci co-localized with PH loci are likely to be new.

### Flag leaf related traits

Wu et al. [56] mapped a FLL QTL on chromosome 6D that overlapped with FLL locus *AX_110876641*. They also reported a pleiotropic locus for FLW and flag leaf angle at about one LD from the present 5A FLL locus *IWB4576*. Another FLL QTL linked with the SSR marker *barc318* identified on chromosome 2B [57] is about 1.2 cM from the present FLL locus *AX_111027654* based on the consensus linkage map [39]. Loci on chromosomes 1A (*AX_109621606*) and 2A (*AX_109880304*) that were stable in four or more environments and BLUE values by both methods are probably new.

A FLW QTL mapped on chromosome 6B by Wu et al. [56] is at the same position as *AX_108771909*, and are probably the same gene. Two stable loci on chromosomes 1A (*AX_111540798*) and 5B (*AX_109519234*) identified in four or five environments and BLUE values in both methods are likely to be new.

Among the 120 loci for GY and related traits, 42 could be the same as QTL reported in previous studies, whereas the remaining are likely to be new. Stable loci identified in both GWAS and QTL mapping showed that they are widespread in varieties. Our results indicated that the methods of GWAS used in the present study were reliable and efficient in detecting loci for GY and related traits.

### Genetic relationships among grain yield and related traits

High-yielding varieties should have good adaptability to prevailing environments, strong resistance to abiotic and biotic stresses, and highly coordinated agronomic traits. Previous studies have showed that improvements in agronomic traits made significant contributions to increased yield potential [4–6]. Many studies have reported interaction effects or genetic linkages among yield related traits, especially in regard to the reduced height loci *Rht-B1* and *Rht-D1* [17, 18, 40, 41, 58]. In the present study, 12 pleiotropic loci involving three or more traits were identified, and more than half of the common loci were co-localized. Previously, three QTL clusters associated with yield related traits were detected at different positions on chromosome 3A [40, 41, 58]; among these the QTL cluster detected by Xu et al. [58] overlapped with the pleiotropic locus *IWA94* in the present study. Many studies have reported that chromosome 5A carries productivity and adaptability related genes [18, 33, 59, 60]. Li et al. [17], Cuthbert et al. [18], Zhang et al. [41] and Liu et al. [48] all reported QTL clusters for yield related traits at different positions on chromosome 5A; however, they are likely to be different from two pleiotropic loci detected in this study. Another locus on chromosome 1B related to GY, PH and UIL is about 15 Mb from the QTL cluster for KNS, KL, PH and FLW identified in Li et al. [17].

Relationships between GY and yield components are discussed in several publications [18, 58, 61–63]. Many studies demonstrated that GY is significantly correlated with SN and KNS. For example, by unconditional and conditional QTL analysis, Xu et al. [58] found that spike number per plant and KNS have larger effects on GY than TKW. Miralles and Slafer [63] reviewed reports on factors influencing GY and concluded that increased GY was associated with increased grain number, but associated with a negative relationship between grain number and grain weight. Huang et al. [61] and Li et al. [62] reported that GY was significantly correlated with kernel size. However, in the present study, co-localization of related loci and phenotypic correlations showed that TKW and KW were more highly correlated with GY than were SN and KNS. Recently, McIntyre et al. [64] detected six putative QTL that increased grain weight and co-located with QTL for SN, KNS and harvest index, and three putative QTL for increased KNS co-located with QTL for increased grain weight, fewer spikes and earlier flowering. In this study, three loci associated with SN and TKW showed opposite effects on these traits due to negative correlation.

Keyes et al. [65] reported that plants with the *Rht-B1b*, *Rht-B1e* and *Rht-D1b* alleles are GA-insensitive, and the reduced PH was induced by decreased sensitivity of their vegetative tissues to endogenous gibberellin (GA). Chebotar et al. [66] pointed out that both GA-sensitive (*Rht8*) and GA-insensitive (*Rht-B1* and *Rht-D1*) dwarfing alleles had effects on almost all investigated traits. Our earlier study on QTL mapping of yield related traits showed that the *Rht-B1* and *Rht-D1* loci, as well as other PH QTL, had significant influences on other traits [17]. In the present study, more than half of the PH and UIL loci were co-localized with other traits, indicating that genes underlying have multiple effects on other traits, including GY.

The growth of wheat is controlled by many genes expressed at different growth stages. Heading and flowering represent a node of spike development and grain-filling, and are affected by environmental conditions as well as the many genes associated with plant development [67]. As a result, HD is crucial in optimising agronomic traits like kernel and spike related phenotypes. However, in the present study, HD exhibited no significant correlations with traits other than FLW. Through co-localization, early heading is likely to benefit kernel development at lower temperatures.

Flag leaves account for 45–58% of the total photosynthetic activity of the plant and contributed 41–43% of the carbohydrates required for grain-filling [68, 69]. Previously, Li et al. [17] found that FLW was important in determining KNS. In the present study, FLL was negatively correlated with GY, whereas it was positively correlated with PH and UIL. However, only few FLL loci were co-localized with loci for GY, PH or UIL. FLW was negatively correlated with SN, but positively correlated with KNS and SDW.

### Potential implications in wheat breeding

The YHRVWZ is the major wheat growing area in China, producing ~ 65% of national production [4]. Comparison of the 20 highest-yielding and other varieties in the germplasm panel showed that KNS, TKW, KW, SDW and FLW in the high-yield group were 2.0, 5.6, 2.6, 7.0 and 4.1%, respectively, higher than the other group, whereas PH, UIL and FLL were 3.5, 10.1 and 5.6% lower. The numbers of alleles for increasing phenotypic values for each trait assessed in the panel were in agreement with the results mentioned above and in favor of Xiao et al. [5] and Gao et al. [6]. However, with the anomaly change of climate and decreased use value of germplasm, yield potential of new varieties is increasing slowly in this area. As a result, new methods and technologies that assisted in selection are essential for further improvement of GY.

High-yielding lines are difficult to select in the early stages of breeding programs as significantly influenced by other traits and environments. Li et al. [17] showed that FLW can be used to select lines with large KNS. In the present study, UIL showed a significant, negative correlation with GY, indicating that larger UIL was associated with decreased carbohydrate transportation to grain. FLL, significantly and positively correlated with UIL, also showed a significant, negative association with GY. SN and KNS were significantly and negatively correlated with each other, as reflected by FLW. Larger FLW was significantly associated with larger KNS and smaller SN in the same variety. Therefore, selection for shorter UIL and FLL would be helpful in selection for higher GY of wheat lines, whereas FLW is convenient to reflect SN and KNS.

Favorable alleles at each locus affecting GY exhibited positive effects on phenotypic values. As a result, the GY loci are valuable for selecting high-yielding varieties in breeding programs. The alleles for increasing phenotypic values presented significant additive effects on each trait, indicating that pyramiding favorable alleles is feasible to improve trait performances using the loci listed in Table 1. Besides, the 12 pleiotropic loci are important in determining GY and related traits, especially the loci that related to GY; the eight loci for TKW (2), KL, KW, SL and PH (3) that at similar positions with the QTL identified in our previous study are also credible. As GY related traits are mostly controlled by polygenes with small effect each, a genome-wide selection would be more powerful in gene discovery and pyramiding breeding with high-density genetic markers or genotyping by sequencing in future. However, MAS may be more feasible as long as only a few QTL need to be tracked in wheat breeding.

Among the 11 varieties with GY potential higher than 8200 kg ha^− 1^, Luyuan 502, Luomai 21, Yannong 18, Shannong 20, Zhongmai 875 and Wanmai 52 possess all 12 favorable alleles for GY. They are good parents to develop new high-yielding varieties. Four varieties, Lumai 8, Zhou 8425B, Zhongmai 875 and 85 Zhong 33 have large TKW, with more than 10 favorable alleles for that trait. These varieties should be valuable germplasms to develop large kernel varieties and for cloning genes related to TKW. Lankao 906 has large spikes with an average KNS of 60.2 and possesses all the favorable alleles identified in the present study for KNS. As KNS in the YHRVWZ is currently not large, this variety can be used to improve KNS. The superior germplasm and favorable alleles of markers identified or confirmed in this study can be used in breeding new high-yielding varieties.

## Conclusions

In the present study, SNP-GWAS and Haplotype-GWAS for GY and related traits, were performed in a diverse panel of 166 varieties with the wheat 90 K and 660 K SNP arrays. One hundred and twenty loci were identified by two methods, and 78 of these are likely to be new. Varieties with higher yield potential identified in the study can be used as parents in breeding programs aimed to accumulate further favorable alleles by marker-assisted selection. Our study proved that two GWAS methods with high-density SNP markers were reliable in identifying genes for GY and related traits, and provided new insight into the genetic architecture of GY.

## Materials and methods

### Plant materials and field trials

The diverse panel used in the present study contained 166 varieties, comprising 144 accessions from the YHRVWZ of China, and 22 accessions from other countries [26].

The diverse panel was grown at Anyang in Henan province and Suixi in Anhui province during the 2012–2013 and 2013–2014 cropping seasons, and at Shijiazhuang in Hebei province and Suixi in Anhui province during the 2014–2015. A randomized complete block design with three replicates was employed in field trials. Each plot comprised three 1.5 m rows spaced 20 cm apart, with 50 plants in each row. Agronomic management was performed according to local practices at each location. All wheat accessions are deposited in the National Genebank of China, Chinese Academy of Agricultural Sciences, and available after approval. All wheat varieties were collected in accordance with national regulations, and the experiments comply with the ethical standards and legislations in China.

### Phenotyping and statistical analysis

Thirteen phenotypic traits, GY, SN, KNS, TKW, KL, KW, SL, SDW, HD, PH, UIL, FLL, and FLW were assessed in the diverse panel (Additional file 1: Table S1).

All plants were harvested in each plot at physiological maturity and GY as kg ha^− 1^ were measured when the moisture declined to 14%. Investigation of the other 12 traits and statistical analyses followed Li et al. [17]. The phenotypic traits GY, KNS, TKW, KL, KW, SL, HD, and PH were assessed in all six environments, whereas data for FLL and FLW and those for SN, SDW and UIL were obtained in five and four environments, respectively. The phenotypic values in each environment and BLUE values were used for GWAS.

### Genotyping, quality control and construction of the physical map

The diverse panel was genotyped using both the wheat 90 K SNP and 660 K SNP arrays [26]. Minor allele frequency (MAF), genetic diversity and PIC were calculated using PowerMarker v3.25 (http://statgen.ncsu.edu/powermarker/). To avoid spurious alleles, SNP with missing data > 20% and MAF < 0.05 were removed. Flanking sequences of SNPs were used to blast against the CSS database (IWGSC RefSeq v1.0, https://urgi.versailles.inra.fr/blast_iwgsc/blast.php) to identify their positions on the physical map. Markers from the two SNP arrays were ordered based on their positions on chromosomes and integrated into a common physical map for GWAS.

### Haplotype analysis

Based on 4 gametes and default parameters as used by the Haploview 4.2 software package (http://www.broadinstitute.org/haploview/haploview), genome-wide haplotype blocks were constructed with PLINK. The number of haplotypes, genetic length (bp) for each block, and the number of tag SNPs based on the ‘solid spine’ of LD were also provided (Extend spine if D′ > 0.8). Haplotype frequency was calculated using a custom Perl script and haplotypes with low frequency (F <0.05) were removed.

### Population structure and linkage disequilibrium

The SNP markers and estimated methods for population structure and LD were the same as in Liu et al. [26]. For population structure, 2000 polymorphic SNP markers evenly distributed on all 21 chromosomes were analyzed in Structure v2.3.4 [70] (http://pritchardlab.stanford.edu/structure.html). PCA and NJ trees were estimated using the software Tassel v5.0 [71] and PowerMarker v3.25 [72] (http://www.maizegenetics.net), respectively, to verify the results.

A total of 12,324 evenly distributed SNP markers were chosen to calculate LD for the A, B and D and entire genomes using the full matrix and sliding window options in Tassel v5.0 [73].

### Genome-wide association studies

SNP-GWAS and Haplotype-GWAS were used to identify the associations between phenotypic and genotypic data. For SNP-GWAS, the mixed linear model (MLM) in Tassel v5.0 was used including kinship matrix and population structure. The kinship matrix was treated as a random effect and calculated by the Tassel v5.0 software, whereas the subpopulation data was considered a fixed effect and estimated by Structure v2.3.4 in MLM analysis. The *P* value indicated the degree of association between a SNP marker and a trait, and the *R*^*2*^ was the variation explained by the significantly associated markers. As the Bonferroni-Holm correction for multiple testing (α = 0.05) was too conserved for the traits in the present study, markers with an adjusted -log10 (*P*-value) ≥ 3.0 were regarded as significant for all traits. For Haplotype-GWAS, PLINK was used in consideration of population structure. According to the results, markers with -log10 (*P*-value) ≥ 4.0 were considered to be significant. Manhattan plots for both methods were drawn using the ggplot2 code in R Language with the *P* value estimated between the marker and trait in Tassel v5.0 and PLINK. In both cases loci identified in one-half or more environments were taken as stable.

### Loci position comparison

For each trait, significant SNP markers within one LD on the same chromosome and identified by the same method were considered to represent one locus. Overlapping loci identified by the two methods for same trait were regarded as common loci. For loci or QTL reported in previous studies, two steps were followed to decide whether currently identified loci were the same as previously found. Firstly, the sequences of the tightly linked or significant markers of the QTL or loci were used to blast against the CSS database (IWGSC RefSeq v1.0, https://urgi.versailles.inra.fr/blast_iwgsc/blast.php). If the marker was less than one LD from the locus for the same trait detected in the present study, they were considered to be the same. Secondly, the consensus linkage map constructed by Maccaferri et al. [39] was used to compare different types of markers. Therefore, loci or QTL were considered to be the same if the tightly linked or significantly associated markers were less than 2.1, 1.2 and 3.9 cM from each other on the A, B and D genomes, respectively.

### Effects of alleles on grain yield and related traits

For each common locus, the most significant SNP markers and haplotypes were chosen as representative markers and haplotypes. The effects of each locus on phenotypic values for GY and the effects of the number of alleles for increasing phenotypic values for each trait were estimated based on the representative markers using R Language.

## Additional files

Additional file 1: **Table S1.** Analysis of phenotypic data for grain yield and related traits in the diverse panel. (DOCX 16 kb)

Additional file 2: **Figure S1.** Distribution of phenotypic values for grain yield and related traits in the diverse panel. GY, grain yield; SN, spike number per square meter; KNS, kernel number per spike; TKW, thousand-kernel weight; KL, kernel length; KW, kernel width; SL, spike length; SDW, spike dry weight; HD, heading date; PH, plant height; UIL, uppermost internode length; FLL, flag leaf length; FLW, flag leaf width. (DOCX 54 kb)

Additional file 3: **Table S2.** Analysis of variance and broad-sense heritabilities (*h*^*2*^) for grain yield and related traits. (DOCX 13 kb)

Additional file 4: **Table S3.** Correlation coefficients among grain yield and related traits in the diverse panel. (DOCX 13 kb)

Additional file 5: **Table S4.** Genome coverage, physical distance and marker polymorphism. (DOCX 16 kb)

Additional file 6: **Figure S2.** Coverage of SNPs (a) and haplotypes (b) on all 21 bread wheat chromosomes. (DOCX 1094 kb)

Additional file 7: **Table S5.** Composition and lengths of blocks and haplotypes. (DOCX 14 kb)

Additional file 8: **Table S6.** Loci for grain yield and related traits identified by SNP-GWAS and Haplotype-GWAS. (XLSX 66 kb)

Additional file 9: **Figure S3.** Manhattan plots for grain yield and related traits in each environment and BLUE value in the diverse panel based on SNP-GWAS. a, grain yield; b, spike number per square meter; c, kernel number per spike; d, thousand-kernel weight; e, kernel length; f, kernel width; g, spike length; h, spike dry weight; i, heading date; j, plant height; k, uppermost internode length; l, flag leaf length; m, flag leaf width; 1, 2012–2013 Anyang; 2, 2012–2013 Suixi; 3, 2013–2014 Anyang; 4, 2013–2014 Suixi; 5, 2014–2015 Anyang; 6, 2014–2015 Shijiazhuang. (DOCX 11525 kb)

Additional file 10: **Figure S4.** Manhattan plots for grain yield and related traits in each environment and BLUE value in the diverse panel based on Haplotype-GWAS. See footnote to Fig. S3 for traits and experimental sites. (DOCX 13093 kb)

Additional file 11: **Table S7.** Phenotypic data in each environment and BLUE value for grain yield and related traits in the diverse panel. GY, grain yield; SN, spike number per square meter; KNS, kernel number per spike; TKW, thousand-kernel weight; KL, kernel length; KW, kernel width; SL, spike length; SDW, spike dry weight; HD, heading date; PH, plant height; UIL, uppermost internode length; FLL, flag leaf length; FLW, flag leaf width. (XLSX 151 kb)

Additional file 12: **Table S8.** Genotypic data for the diverse panel of 166 elite wheat varieties. (XLSX 218034 kb)

## Abbreviations

BLUE: Best linear unbiased estimation
FLL: Flag leaf length
FLW: Flag leaf width
GWAS: Genome-wide association study
GY: Grain yield
*h*^2^: Broad-sense heritability
HD: Heading date
KASP: Kompetitive allele-specific PCR
KL: Kernel length
KNS: Kernel number per spike
KW: Kernel width
LD: Linkage disequilibrium
MAF: Minor allele frequency
MAS: Marker-assisted selection
MLM: Mixed linear model
PH: Plant height
PIC: Polymorphism information content
QTL: Quantitative trait loci
*R*^*2*^: Phenotypic variance explained
RIL: Recombinant inbred line
SDW: Spike dry weight
SL: Spike length
SN: Spike number per unit area
SNP: Single nucleotide polymorphism
SSR: Simple sequence repeat
TKW: Thousand-kernel weight
UIL: Uppermost internode length
YHRVWD: Yellow and Huai River Valleys Wheat Zone
