# Supplementary material for: Genetic architecture of grain yield in bread wheat based on genome-wide association studies
Source: BMC Plant Biol. 2019 Apr 29;19:168. doi: 10.1186/s12870-019-1781-3 (PMC6489268; doi:10.1186/s12870-019-1781-3)
Supplement: Supplementary file 1 — Table S1. Analysis of phenotypic data for grain yield and related traits in the diverse panel. (DOCX 16 kb) [file 12870_2019_1781_MOESM1_ESM.docx]

Table S1 Analysis of phenotypic data for grain yield and related traits in the diverse panel

| Trait | Environment | Mean | SD | Range | CV |
| --- | --- | --- | --- | --- | --- |
| GY (kg ha^-1^) | 2012AY | 9318.2 | 1144.7 | 6267.9–12,488.9 | 12.3 |
|  | 2012SX | 6320.3 | 989.4 | 3068.4–9074.1 | 15.7 |
|  | 2013AY | 9178.8 | 1126.3 | 5859.8–12,547.1 | 12.3 |
|  | 2013SX | 8562.4 | 1067.8 | 5065.3–11,373.8 | 12.5 |
|  | 2014AY | 7328.3 | 1046.2 | 4677.8–9355.6 | 14.3 |
|  | 2014SJZ | 6156.0 | 813.9 | 3638.9–7683.3 | 13.2 |
|  | BLUE | 7268.8 | 740.3 | 5090.5–8535.8 | 10.2 |
| SN (m^-2^) | 2012AY | 693.3 | 105.8 | 356.4–943.1 | 15.3 |
|  | 2012SX | 533.5 | 92.6 | 276.4–804.4 | 17.4 |
|  | 2013AY | 577.8 | 83.8 | 290.7–846.2 | 14.5 |
|  | 2013SX | 632.3 | 88.8 | 220.4–891.6 | 14.0 |
|  | BLUE | 604.9 | 81.7 | 296.5–833.7 | 13.5 |
| KNS | 2012AY | 38.7 | 6.6 | 23.6–64.6 | 17.0 |
|  | 2012SX | 38.6 | 6.7 | 24.1–38.1 | 17.5 |
|  | 2013AY | 39.8 | 6.8 | 26.1–70.3 | 17.1 |
|  | 2013SX | 39.6 | 6.1 | 24.0–62.1 | 15.4 |
|  | 2014AY | 39.6 | 6.0 | 25.3–59.8 | 15.2 |
|  | 2014SJZ | 36.4 | 5.6 | 25.6–57.6 | 15.3 |
|  | BLUE | 38.4 | 5.6 | 26.8–61.0 | 14.7 |
| TKW (g) | 2012AY | 41.1 | 5.2 | 25.3–56.1 | 12.7 |
|  | 2012SX | 42.2 | 5.7 | 25.7–56.3 | 13.4 |
|  | 2013AY | 49.8 | 5.5 | 28.3–63.4 | 11.1 |
|  | 2013SX | 45.8 | 6.1 | 22.5–62.2 | 13.2 |
|  | 2014AY | 39.4 | 4.7 | 24.8–50.7 | 11.8 |
|  | 2014SJZ | 39.6 | 5.1 | 23.0–50.0 | 12.8 |
|  | BLUE | 43.2 | 4.9 | 25.7–54.5 | 11.5 |
| KL (mm) | 2012AY | 6.8 | 0.4 | 5.9–7.7 | 5.3 |
|  | 2012SX | 6.6 | 0.3 | 5.8–7.6 | 5.2 |
|  | 2013AY | 7.4 | 0.4 | 6.6–8.3 | 4.8 |
|  | 2013SX | 7.4 | 0.4 | 6.6–8.5 | 4.8 |
|  | 2014AY | 7.1 | 0.3 | 6.4–8.1 | 4.9 |
|  | 2014SJZ | 7.0 | 0.4 | 6.1–8.2 | 5.2 |
|  | BLUE | 7.1 | 0.3 | 6.3–8.1 | 4.7 |
| KW (mm) | 2012AY | 3.3 | 0.2 | 2.7–3.7 | 5.5 |
|  | 2012SX | 3.3 | 0.2 | 2.5–3.7 | 6.3 |
|  | 2013AY | 3.7 | 0.2 | 2.9–4.0 | 4.8 |
|  | 2013SX | 3.6 | 0.2 | 2.7–4.0 | 5.5 |
|  | 2014AY | 3.4 | 0.2 | 2.8–3.8 | 5.2 |
|  | 2014SJZ | 3.3 | 0.2 | 2.5–3.8 | 5.9 |
|  | BLUE | 3.5 | 0.2 | 2.7–3.9 | 4.8 |
| SL (cm) | 2012AY | 8.9 | 0.9 | 6.8–12.7 | 10.5 |
|  | 2012SX | 9.6 | 0.9 | 7.9–13.7 | 9.5 |
|  | 2013AY | 8.8 | 0.9 | 6.7–11.4 | 10.5 |
|  | 2013SX | 9.5 | 1.1 | 7.2–12.4 | 11.1 |
|  | 2014AY | 9.6 | 0.9 | 7.6–12.8 | 9.7 |
|  | 2014SJZ | 8.9 | 0.9 | 7.2–12.8 | 10.4 |
|  | BLUE | 9.2 | 0.9 | 7.4–12.3 | 9.4 |
| SDW (g) | 2013AY | 1.99 | 0.37 | 1.14–3.83 | 18.4 |
|  | 2013SX | 1.79 | 0.31 | 1.12–2.79 | 17.1 |
|  | 2014AY | 1.67 | 0.32 | 0.97–2.91 | 19.4 |
|  | 2014SJZ | 1.42 | 0.31 | 0.68–2.78 | 22.0 |
|  | BLUE | 1.63 | 0.29 | 1.03–2.97 | 17.8 |
| HD (d) | 2012AY | 198.2 | 2.2 | 190.0–208.7 | 1.1 |
|  | 2012SX | 189.9 | 3.3 | 184.0–203.7 | 1.7 |
|  | 2013AY | 185.7 | 3.3 | 177.3–201.3 | 1.8 |
|  | 2013SX | 184.6 | 3.1 | 178.0–201.0 | 1.7 |
|  | 2014AY | 195.9 | 2.3 | 188.3–206.3 | 1.2 |
|  | 2014SJZ | 199.6 | 2.2 | 195.3–215.0 | 1.1 |
|  | BLUE | 194.2 | 1.6 | 190.3–200.6 | 0.8 |
| PH (cm) | 2012AY | 84.1 | 12.7 | 63.3–145.0 | 15.1 |
|  | 2012SX | 80.1 | 11.3 | 55.0–133.3 | 14.2 |
|  | 2013AY | 77.6 | 12.6 | 58.2–135.7 | 16.2 |
|  | 2013SX | 82.9 | 10.8 | 59.8–130.1 | 13.0 |
|  | 2014AY | 91.5 | 10.1 | 69.9–136.3 | 11.0 |
|  | 2014SJZ | 81.8 | 9.4 | 63.4–120.1 | 11.5 |
|  | BLUE | 83.4 | 10.6 | 62.6–132.4 | 12.7 |
| UIL (cm) | 2012AY | 26.3 | 4.7 | 19.2–49.4 | 17.9 |
|  | 2012SX | 27.1 | 4.5 | 20.1–44.4 | 16.8 |
|  | 2014AY | 27.8 | 4.4 | 19.8–47.1 | 15.7 |
|  | 2014SJZ | 27.6 | 4.0 | 19.6–40.8 | 14.4 |
|  | BLUE | 27.4 | 4.1 | 19.9–43.7 | 14.8 |
| FLL (cm) | 2012SX | 18.0 | 2.1 | 12.6–24.4 | 11.5 |
|  | 2013AY | 16.7 | 2.3 | 12.4–24.5 | 13.8 |
|  | 2013SX | 19.5 | 2.4 | 13.3–27.3 | 12.2 |
|  | 2014AY | 18.8 | 2.6 | 14.0–28.1 | 13.7 |
|  | 2014SJZ | 17.3 | 2.3 | 13.1–24.1 | 13.4 |
|  | BLUE | 17.9 | 2.1 | 13.9–24.9 | 11.7 |
| FLW (cm) | 2012SX | 1.69 | 0.18 | 1.20–2.17 | 10.7 |
|  | 2013AY | 1.58 | 0.16 | 1.17–2.11 | 9.9 |
|  | 2013SX | 1.76 | 0.18 | 1.30–2.25 | 10.0 |
|  | 2014AY | 1.87 | 0.17 | 1.43–2.37 | 9.3 |
|  | 2014SJZ | 1.65 | 0.16 | 1.29–2.19 | 9.9 |
|  | BLUE | 1.70 | 0.15 | 1.35–2.18 | 9.1 |

GY, grain yield; SN, spike number per square meter; KNS, kernel number per spike; TKW, thousand-kernel weight; KL, kernel length; KW, kernel width; SL, spike length; SDW, spike dry weight; HD, heading date; PH, plant height; UIL, uppermost internode length; FLL, flag leaf length; FLW, flag leaf width; AY: Anyang; SX: Suixi; SJZ: Shijiazhuang; BLUE: Best linear unbiased estimation
