# Supplementary material for: Genetic architecture of grain yield in bread wheat based on genome-wide association studies
Source: BMC Plant Biol. 2019 Apr 29;19:168. doi: 10.1186/s12870-019-1781-3 (PMC6489268; doi:10.1186/s12870-019-1781-3)
Supplement: Supplementary file 2 — Figure S1. Distribution of phenotypic values for grain yield and related traits in the diverse panel. GY, grain yield; SN, spike number per square meter; KNS, kernel number per spike; TKW, thousand-kernel weight; KL, kernel length; KW, kernel width; SL, spike length; SDW, spike dry weight; HD, heading date; PH, plant height; UIL, uppermost internode length; FLL, flag leaf length; FLW, flag leaf width. (DOCX 54 kb) [file 12870_2019_1781_MOESM2_ESM.docx]

Fig. S1 Distribution of phenotypic values for grain yield and related traits in the diverse panel. GY, grain yield; SN, spike number per square meter; KNS, kernel number per spike; TKW, thousand-kernel weight; KL, kernel length; KW, kernel width; SL, spike length; SDW, spike dry weight; HD, heading date; PH, plant height; UIL, uppermost internode length; FLL, flag leaf length; FLW, flag leaf width
