# Supplementary material for: Genetic architecture of grain yield in bread wheat based on genome-wide association studies
Source: BMC Plant Biol. 2019 Apr 29;19:168. doi: 10.1186/s12870-019-1781-3 (PMC6489268; doi:10.1186/s12870-019-1781-3)
Supplement: Supplementary file 3 — Table S2. Analysis of variance and broad-sense heritabilities (h2) for grain yield and related traits. (DOCX 13 kb) [file 12870_2019_1781_MOESM3_ESM.docx]

Table S2 Analysis of variance and broad-sense heritabilities (*h^2^*) for grain yield and related traits

| Trait | Mean square | | | | | *h^2^* |
| --- | --- | --- | --- | --- | --- | --- |
|  | Line (L) | Environment (E) | Replicate | L×E | Error |  |
| GY | 10,296,769^**^ | 986,178,880^**^ | 5,298,114^**^ | 1,813,629^**^ | 264,245 | 0.72 |
| SN | 82,536.2^**^ | 2,376,391.0^**^ | 45,276.1^**^ | 7159.2^**^ | 1106.1 | 0.89 |
| KNS | 593.5^**^ | 792.7^**^ | 290.7^**^ | 25.2^**^ | 8.9 | 0.93 |
| TKW | 451.3^**^ | 8282.6^**^ | 97.3^**^ | 14.2^**^ | 4.7 | 0.95 |
| KL | 2.03^**^ | 51.98^**^ | 0.43^**^ | 0.05^**^ | 0.03 | 0.97 |
| KW | 0.51^**^ | 13.41^**^ | 0.21^**^ | 0.03^**^ | 0.02 | 0.93 |
| SL | 13.4^**^ | 72.4^**^ | 5.7^**^ | 0.6^**^ | 0.3 | 0.93 |
| SDW | 1.01^**^ | 28.37^**^ | 0.04 | 0.10^**^ | 0.05 | 0.89 |
| HD | 121.1^**^ | 20935.5^**^ | 16.6^**^ | 3.9^**^ | 0.5 | 0.94 |
| PH | 2048.8^**^ | 11235.2^**^ | 46.7^*^ | 43.5^**^ | 10.7 | 0.96 |
| UIL | 209.8^**^ | 225.9^**^ | 63.4^**^ | 7.7^**^ | 4.1 | 0.96 |
| FLL | 61.1^**^ | 617.4^**^ | 14.9^**^ | 5.2^**^ | 3.0 | 0.89 |
| FLW | 0.36^**^ | 6.20^**^ | 0.02 | 0.02^**^ | 0.01 | 0.94 |

GY, grain yield; SN, spike number per square meter; KNS, kernel number per spike; TKW, thousand-kernel weight; KL, kernel length; KW, kernel width; SL, spike length; SDW, spike dry weight; HD, heading date; PH, plant height; UIL, uppermost internode length; FLL, flag leaf length; FLW, flag leaf width
