# Supplementary material for: Genetic architecture of grain yield in bread wheat based on genome-wide association studies
Source: BMC Plant Biol. 2019 Apr 29;19:168. doi: 10.1186/s12870-019-1781-3 (PMC6489268; doi:10.1186/s12870-019-1781-3)
Supplement: Supplementary file 4 — Table S3. Correlation coefficients among grain yield and related traits in the diverse panel. (DOCX 13 kb) [file 12870_2019_1781_MOESM4_ESM.docx]

Table S3 Correlation coefficients among grain yield and related traits in the diverse panel

|  | GY | SN | KNS | TKW | KL | KW | SL | SDW | HD | PH | UIL | FLL |
| --- | --- | --- | --- | --- | --- | --- | --- | --- | --- | --- | --- | --- |
| SN | -0.14 |  |  |  |  |  |  |  |  |  |  |  |
| KNS | 0.01 | -0.58^**^ |  |  |  |  |  |  |  |  |  |  |
| TKW | 0.39^**^ | -0.38^**^ | -0.26^**^ |  |  |  |  |  |  |  |  |  |
| KL | 0.10 | -0.08 | -0.24^**^ | 0.53^**^ |  |  |  |  |  |  |  |  |
| KW | 0.45^**^ | -0.43^**^ | -0.06 | 0.83^**^ | 0.20 |  |  |  |  |  |  |  |
| SL | -0.10 | -0.30^**^ | 0.36^**^ | 0.01 | 0.13 | -0.04 |  |  |  |  |  |  |
| SDW | 0.25^**^ | -0.70^**^ | 0.64^**^ | 0.50^**^ | 0.19 | 0.57^**^ | 0.31^**^ |  |  |  |  |  |
| HD | -0.16 | 0.17 | -0.10 | -0.14 | -0.04 | -0.14 | -0.07 | -0.19 |  |  |  |  |
| PH | -0.45^**^ | 0.27^**^ | 0.06 | -0.16 | 0.02 | -0.20 | 0.17 | 0.08 | 0.12 |  |  |  |
| UIL | -0.53^**^ | 0.29^**^ | -0.07 | -0.18 | 0.05 | -0.27^**^ | 0.12 | -0.06 | 0.08 | 0.78^**^ |  |  |
| FLL | -0.52^**^ | -0.03 | 0.23^**^ | -0.22^**^ | 0.06 | -0.28^**^ | 0.29^**^ | 0.07 | 0.10 | 0.45^**^ | 0.57^**^ |  |
| FLW | 0.28^**^ | -0.58^**^ | 0.43^**^ | 0.28^**^ | -0.05 | 0.31^**^ | 0.11 | 0.51^**^ | -0.35^**^ | -0.38^**^ | -0.38^**^ | -0.16 |

GY, grain yield; SN, spike number per square meter; KNS, kernel number per spike; TKW, thousand-kernel weight; KL, kernel length; KW, kernel width; SL, spike length; SDW, spike dry weight; HD, heading date; PH, plant height; UIL, uppermost internode length; FLL, flag leaf length; FLW, flag leaf width
