# Supplementary material for: Genetic architecture of grain yield in bread wheat based on genome-wide association studies
Source: BMC Plant Biol. 2019 Apr 29;19:168. doi: 10.1186/s12870-019-1781-3 (PMC6489268; doi:10.1186/s12870-019-1781-3)
Supplement: Supplementary file 5 — Table S4. Genome coverage, physical distance and marker polymorphism. (DOCX 16 kb) [file 12870_2019_1781_MOESM5_ESM.docx]

Table S4 Genome coverage, physical distance and marker polymorphism

| Chr | Number of markers | Length (Mb) | Mb/marker | Genetic diversity | PIC |
| --- | --- | --- | --- | --- | --- |
| 1A | 22,097 | 594.0 | 0.027 | 0.34 | 0.28 |
| 1B | 19,906 | 689.4 | 0.035 | 0.39 | 0.31 |
| 1D | 7053 | 495.4 | 0.070 | 0.32 | 0.26 |
| 2A | 22,210 | 780.8 | 0.035 | 0.32 | 0.26 |
| 2B | 25,492 | 801.3 | 0.031 | 0.33 | 0.26 |
| 2D | 6874 | 651.8 | 0.095 | 0.36 | 0.29 |
| 3A | 15,126 | 750.8 | 0.050 | 0.34 | 0.27 |
| 3B | 41,439 | 830.7 | 0.020 | 0.34 | 0.28 |
| 3D | 4154 | 615.5 | 0.148 | 0.32 | 0.26 |
| 4A | 13,946 | 744.5 | 0.053 | 0.32 | 0.26 |
| 4B | 10,273 | 673.5 | 0.066 | 0.34 | 0.28 |
| 4D | 2061 | 509.8 | 0.247 | 0.31 | 0.25 |
| 5A | 17,097 | 709.8 | 0.042 | 0.40 | 0.31 |
| 5B | 29,146 | 713.0 | 0.024 | 0.38 | 0.30 |
| 5D | 4659 | 566.0 | 0.121 | 0.33 | 0.27 |
| 6A | 15,121 | 618.0 | 0.041 | 0.35 | 0.28 |
| 6B | 21,355 | 721.0 | 0.034 | 0.35 | 0.28 |
| 6D | 4255 | 473.5 | 0.111 | 0.31 | 0.25 |
| 7A | 24,111 | 736.7 | 0.031 | 0.33 | 0.27 |
| 7B | 13,571 | 750.6 | 0.055 | 0.36 | 0.29 |
| 7D | 6624 | 638.7 | 0.096 | 0.30 | 0.25 |
| Total genome A | 129,708 | 4934.6 | 0.038 | 0.34 | 0.28 |
| Total genome B | 161,182 | 5179.4 | 0.032 | 0.35 | 0.28 |
| Total genome D | 35,680 | 3950.8 | 0.111 | 0.32 | 0.26 |
| Total | 326,570 | 14064.8 | 0.043 | 0.34 | 0.27 |
