# Supplementary figures and images for: Genetic architecture of grain yield in bread wheat based on genome-wide association studies
Source: BMC Plant Biol. 2019 Apr 29;19:168. doi: 10.1186/s12870-019-1781-3 (PMC6489268; doi:10.1186/s12870-019-1781-3)

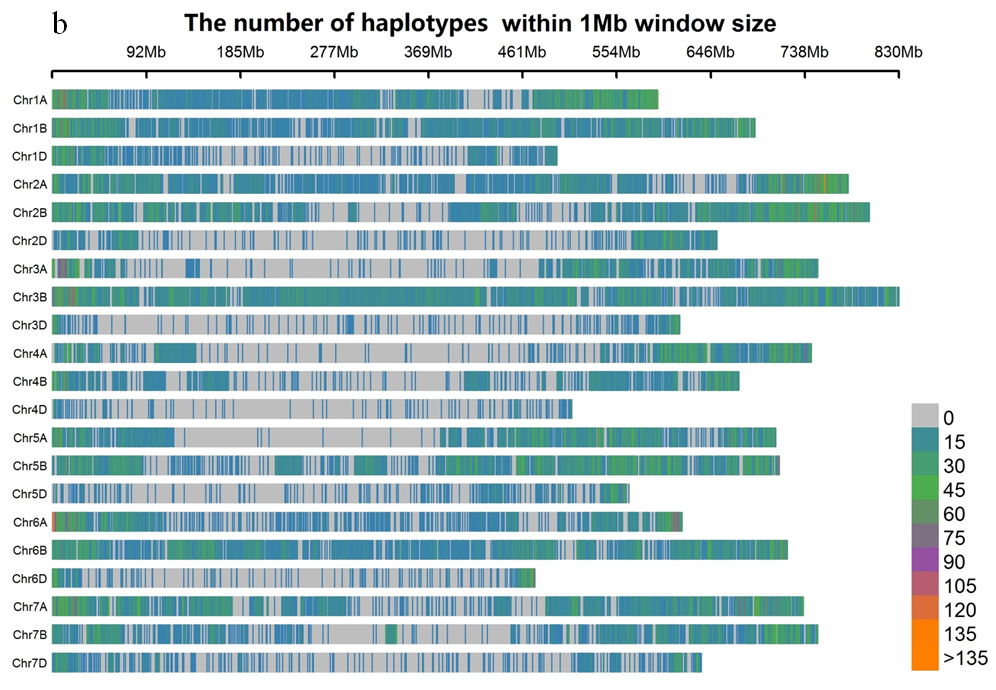

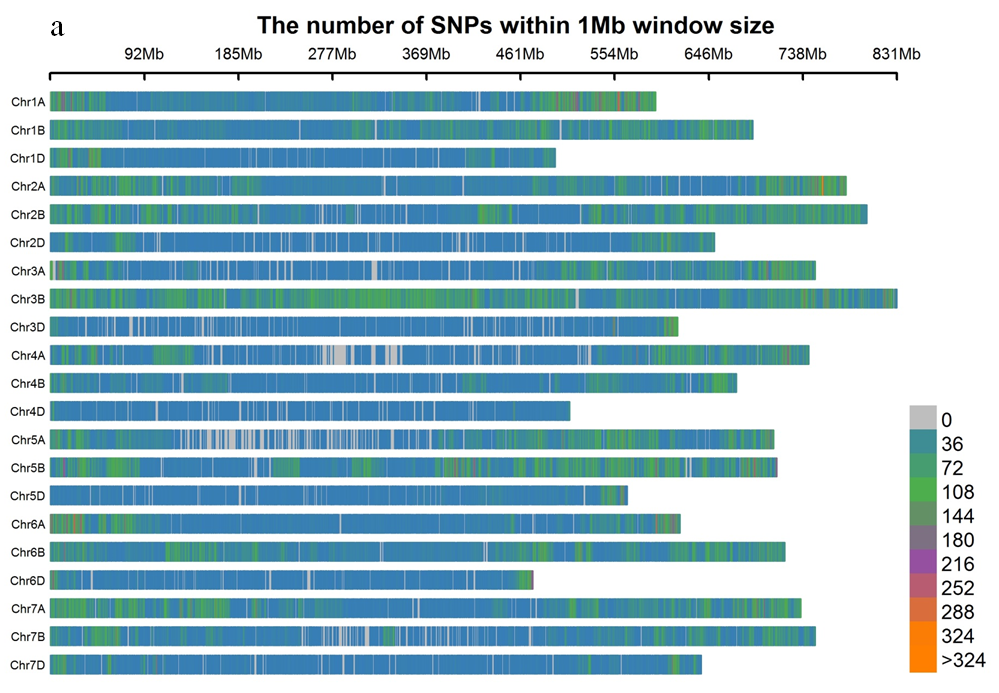


Fig. S2 Coverage of SNPs (a) and haplotypes (b) on all 21 bread wheat chromosomes

Supplement: Supplementary file 6 — Figure S2. Coverage of SNPs (a) and haplotypes (b) on all 21 bread wheat chromosomes. (DOCX 1094 kb) [file 12870_2019_1781_MOESM6_ESM.docx]
