# Supplementary material for: Genetic architecture of grain yield in bread wheat based on genome-wide association studies
Source: BMC Plant Biol. 2019 Apr 29;19:168. doi: 10.1186/s12870-019-1781-3 (PMC6489268; doi:10.1186/s12870-019-1781-3)
Supplement: Supplementary file 7 — Table S5. Composition and lengths of blocks and haplotypes. (DOCX 14 kb) [file 12870_2019_1781_MOESM7_ESM.docx]

Table S5 Composition and lengths of blocks and haplotypes

| Chr | Number of  blocks | Number of  haplotypes | Markers/  haplotype | Average length  (kb) | Min length  (kb) | Max length  (kb) |
| --- | --- | --- | --- | --- | --- | --- |
| 1A | 2137 | 7838 | 9.1 | 86.76 | 0.001 | 200.00 |
| 1B | 2232 | 8179 | 7.8 | 87.14 | 0.001 | 199.99 |
| 1D | 697 | 2147 | 7.3 | 52.71 | 0.002 | 199.99 |
| 2A | 2343 | 8330 | 7.9 | 86.45 | 0.001 | 200.00 |
| 2B | 2638 | 9963 | 8.4 | 85.13 | 0.001 | 200.00 |
| 2D | 716 | 2426 | 6.4 | 59.31 | 0.002 | 199.99 |
| 3A | 1390 | 5358 | 9.0 | 83.57 | 0.002 | 199.99 |
| 3B | 3582 | 13,750 | 10.8 | 112.65 | 0.002 | 200.00 |
| 3D | 356 | 1070 | 7.0 | 47.41 | 0.003 | 200.00 |
| 4A | 1331 | 4815 | 8.7 | 80.98 | 0.002 | 199.99 |
| 4B | 1150 | 3746 | 7.2 | 90.22 | 0.002 | 200.00 |
| 4D | 154 | 447 | 4.3 | 24.79 | 0.003 | 199.57 |
| 5A | 1652 | 6212 | 9.2 | 93.34 | 0.002 | 199.99 |
| 5B | 2394 | 9410 | 11.0 | 97.94 | 0.009 | 200.00 |
| 5D | 431 | 1373 | 5.7 | 39.51 | 0.002 | 199.96 |
| 6A | 1412 | 5373 | 8.9 | 77.36 | 0.002 | 199.99 |
| 6B | 2278 | 8543 | 8.1 | 96.58 | 0.002 | 200.00 |
| 6D | 414 | 1303 | 5.5 | 35.43 | 0.003 | 199.99 |
| 7A | 2309 | 8965 | 9.1 | 92.57 | 0.003 | 200.00 |
| 7B | 1516 | 5494 | 7.4 | 85.45 | 0.001 | 199.99 |
| 7D | 616 | 1813 | 6.6 | 53.67 | 0.001 | 199.99 |
| Total genome A | 12,574 | 46,891 | 8.8 | 85.86 | 0.002 | 199.99 |
| Total genome B | 15,790 | 59,085 | 8.7 | 93.59 | 0.003 | 199.99 |
| Total genome D | 3384 | 10,579 | 6.1 | 44.69 | 0.002 | 199.93 |
| Total | 31,748 | 116,555 | 7.9 | 74.71 | 0.002 | 199.98 |
