# Supplementary material for: Genetic architecture of grain yield in bread wheat based on genome-wide association studies
Source: BMC Plant Biol. 2019 Apr 29;19:168. doi: 10.1186/s12870-019-1781-3 (PMC6489268; doi:10.1186/s12870-019-1781-3)
Supplement: Supplementary file 9 — Figure S3. Manhattan plots for grain yield and related traits in each environment and BLUE value in the diverse panel based on SNP-GWAS. a, grain yield; b, spike number per square meter; c, kernel number per spike; d, thousand-kernel weight; e, kernel length; f, kernel width; g, spike length; h, spike dry weight; i, heading date; j, plant height; k, uppermost internode length; l, flag leaf length; m, flag leaf width; 1, 2012–2013 Anyang; 2, 2012–2013 Suixi; 3, 2013–2014 Anyang; 4, 2013–2014 Suixi; 5, 2014–2015 Anyang; 6, 2014–2015 Shijiazhuang. (DOCX 11525 kb) [file 12870_2019_1781_MOESM9_ESM.docx]

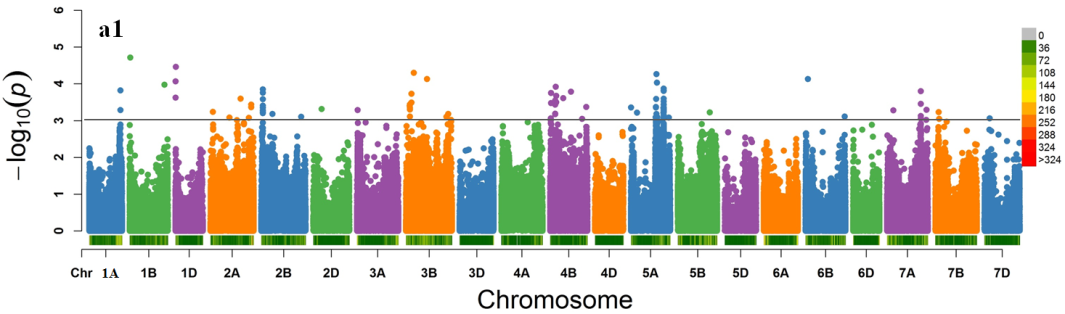

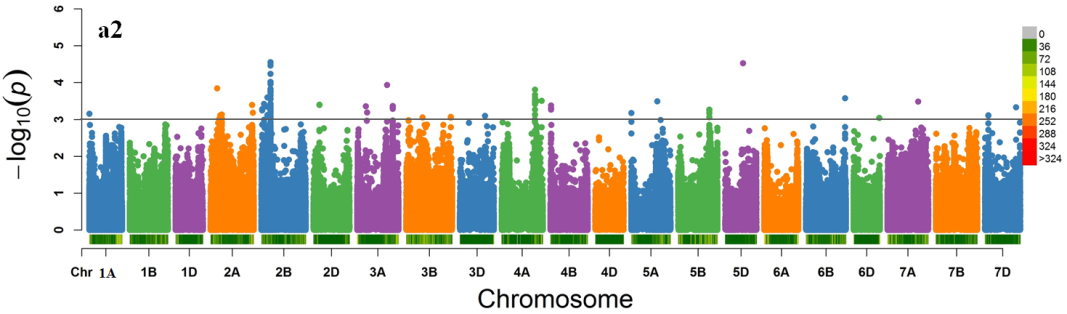

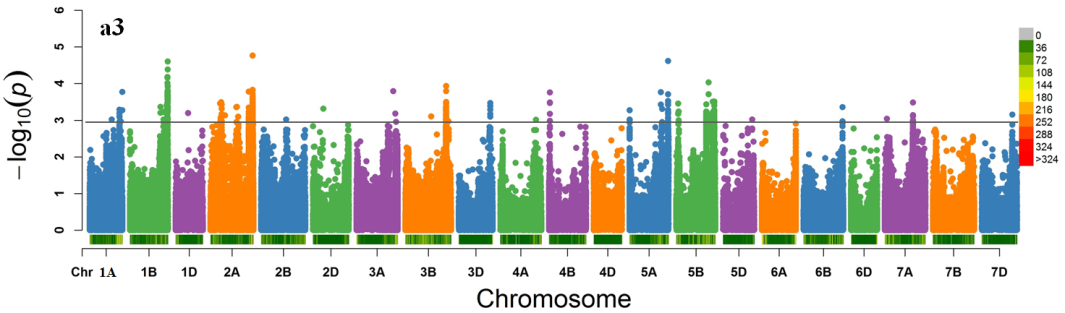

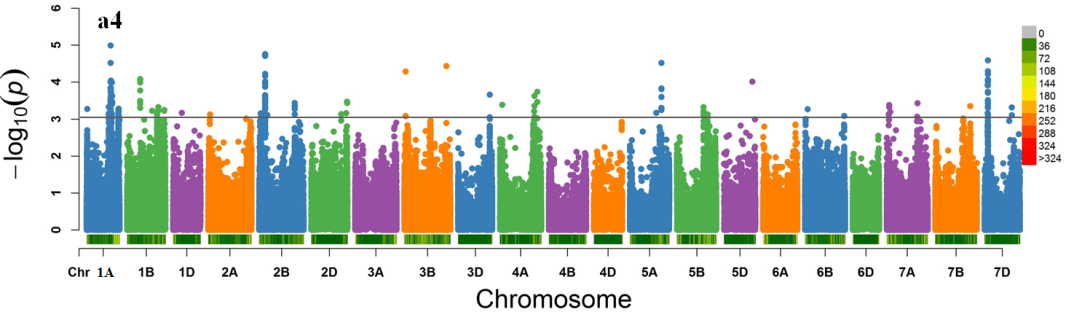

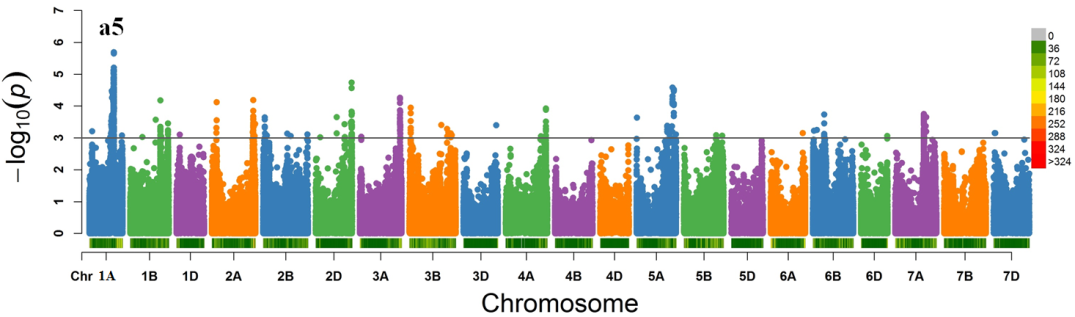

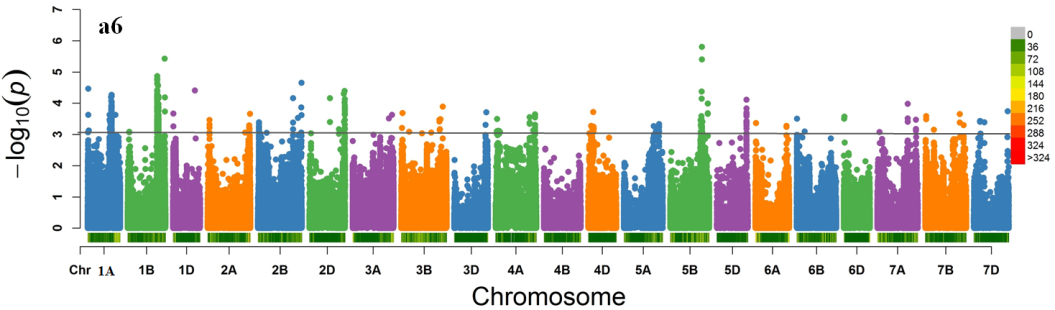

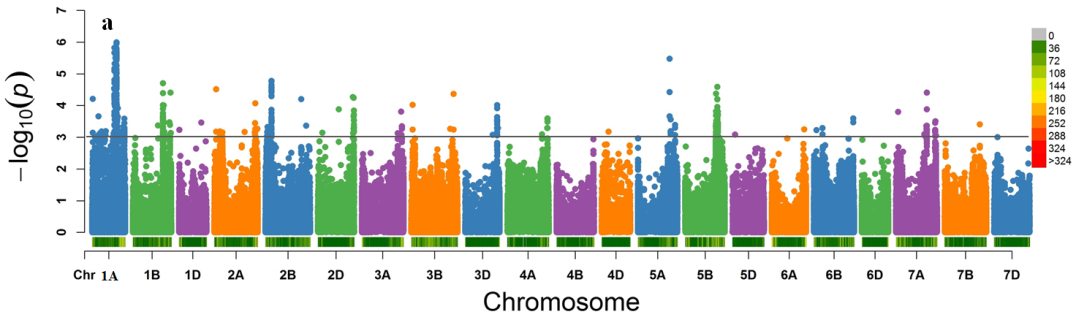


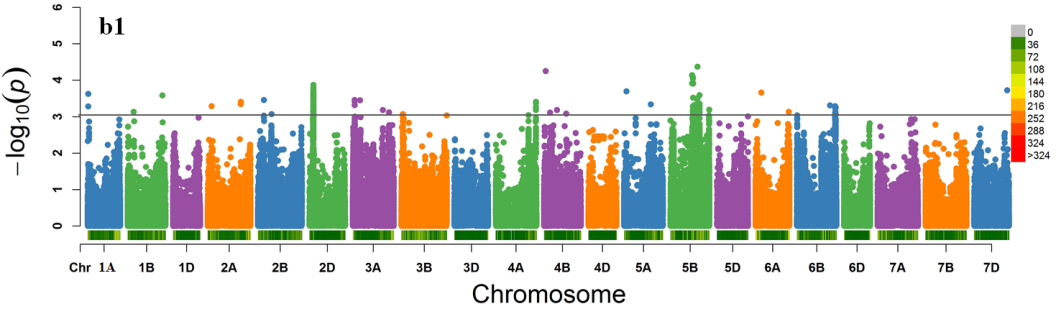

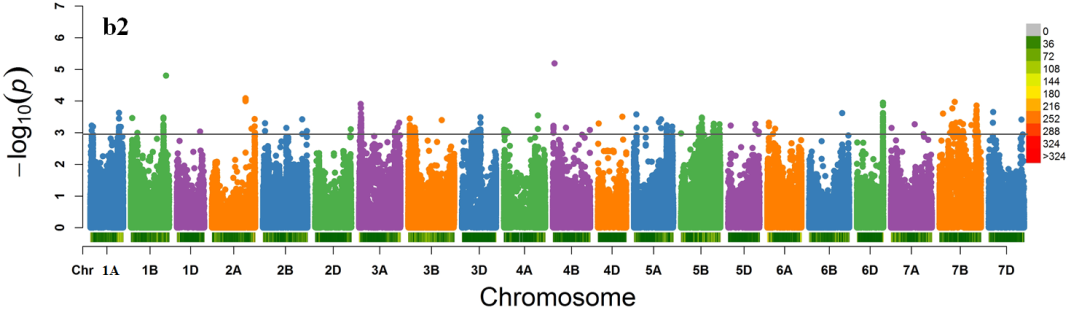

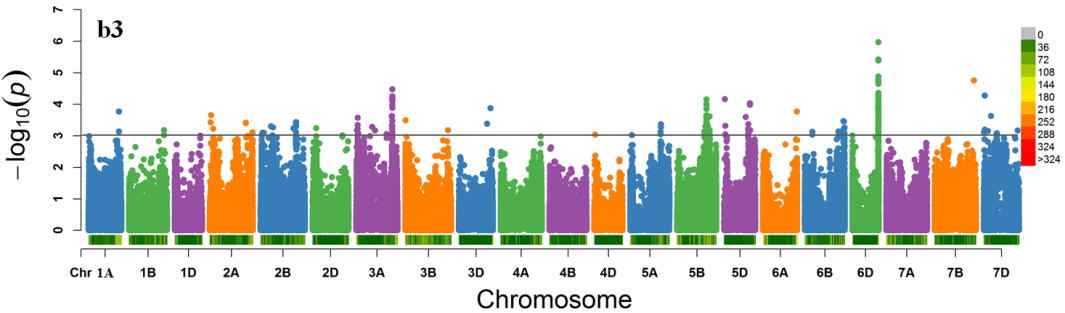

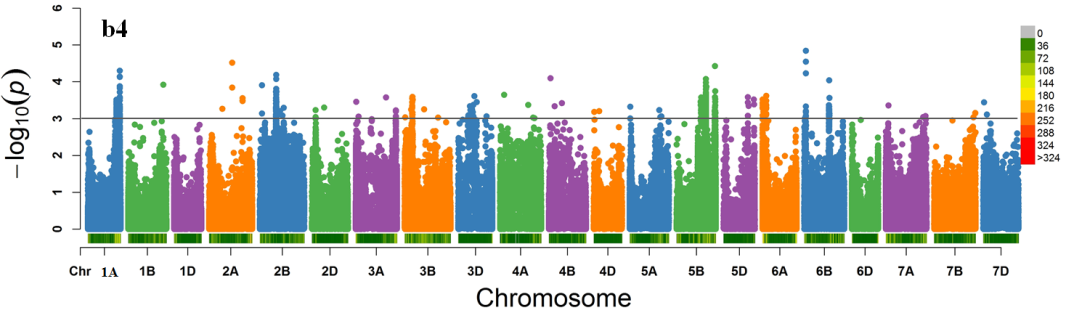

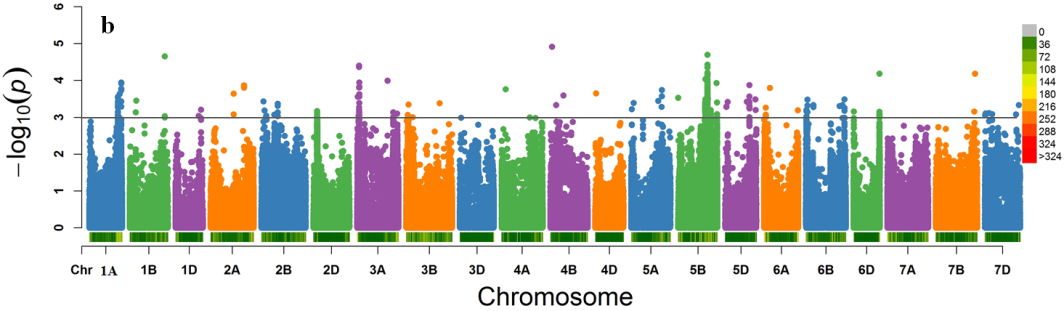


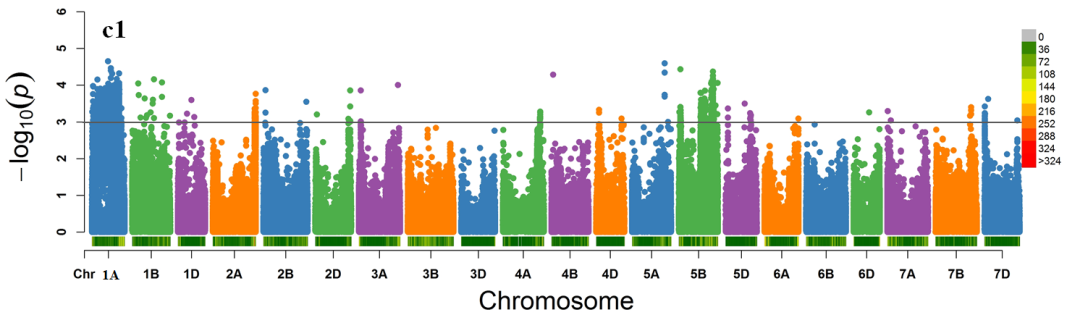

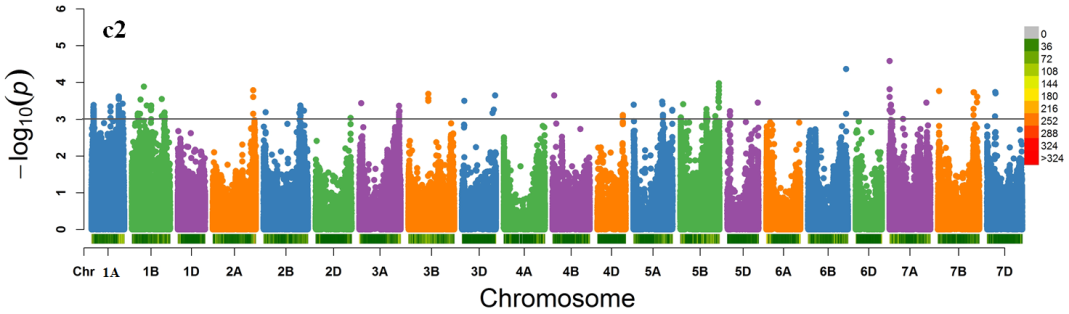

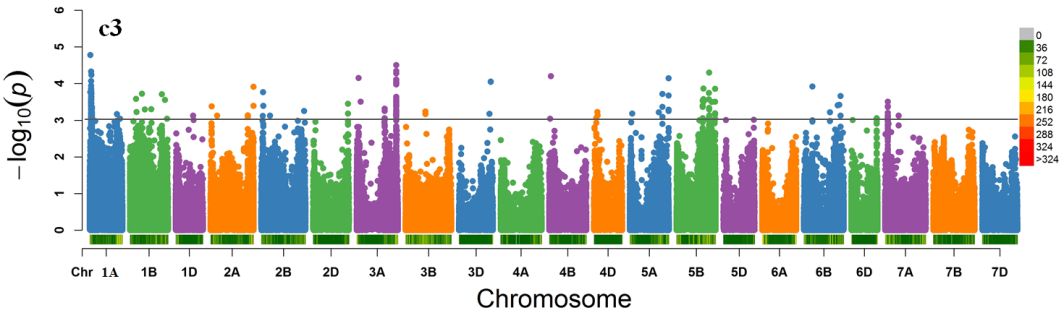

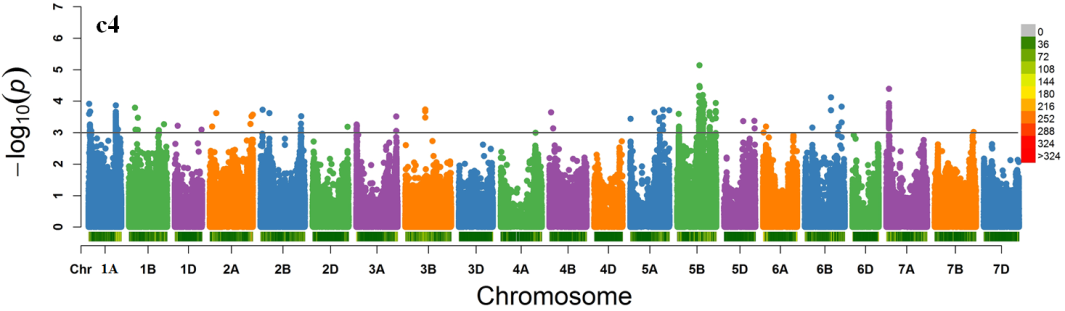

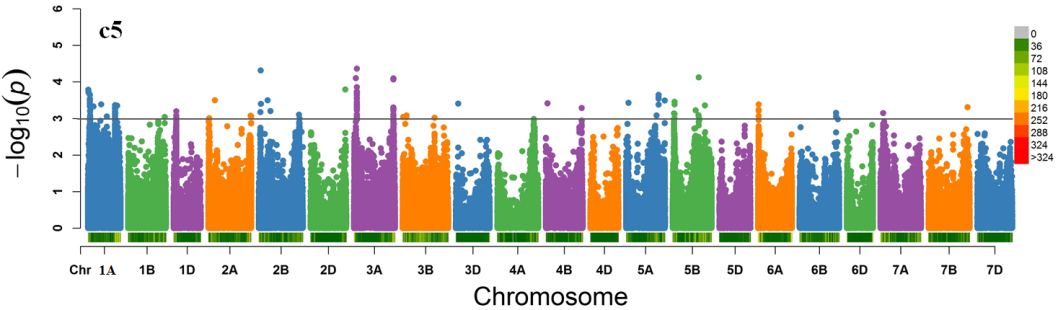

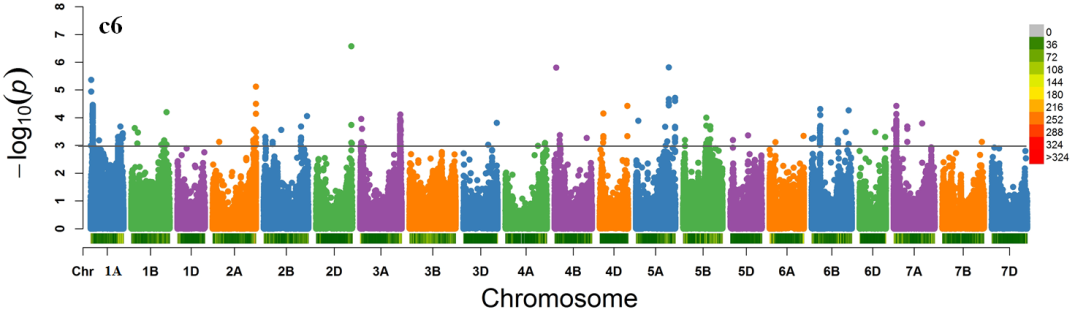

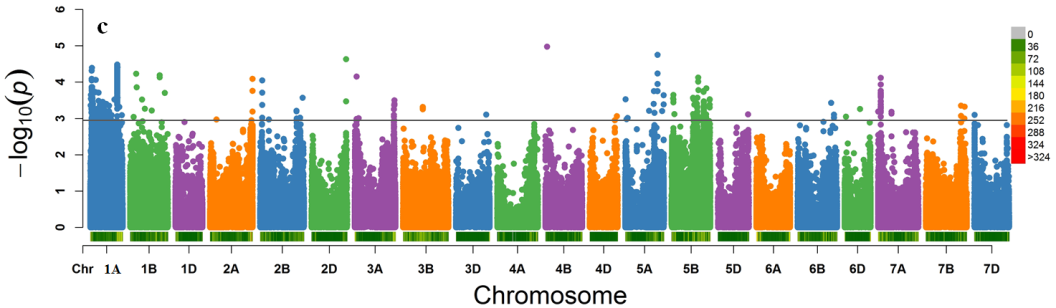


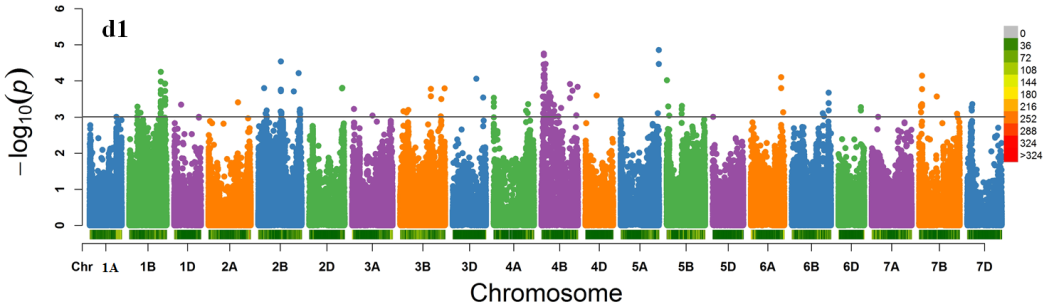

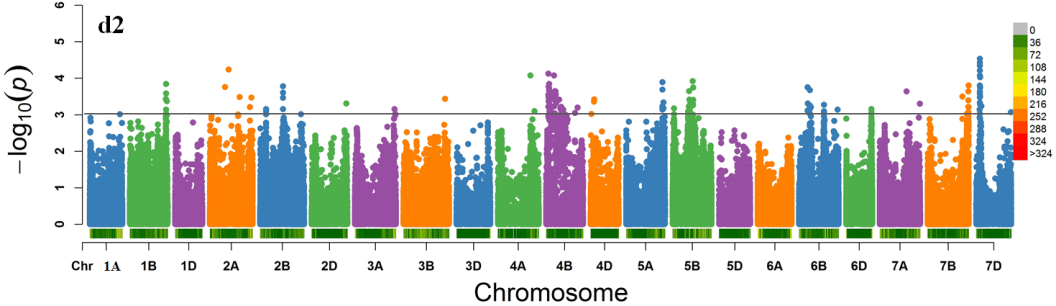

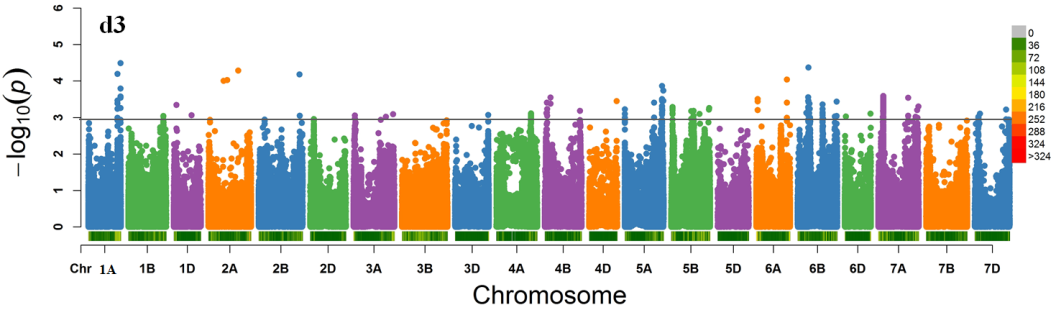

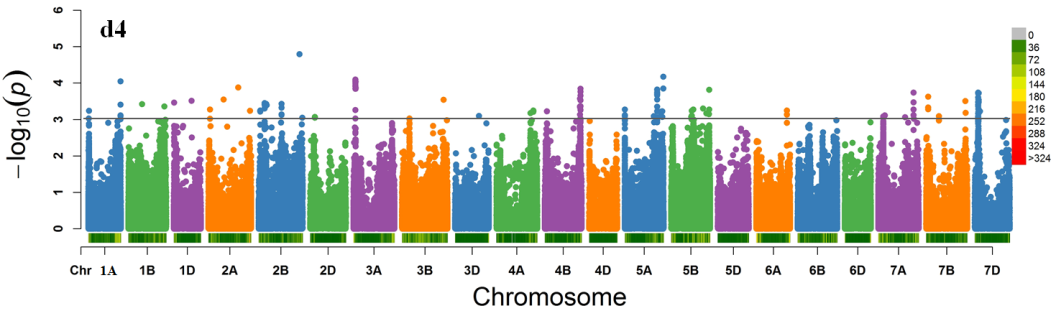

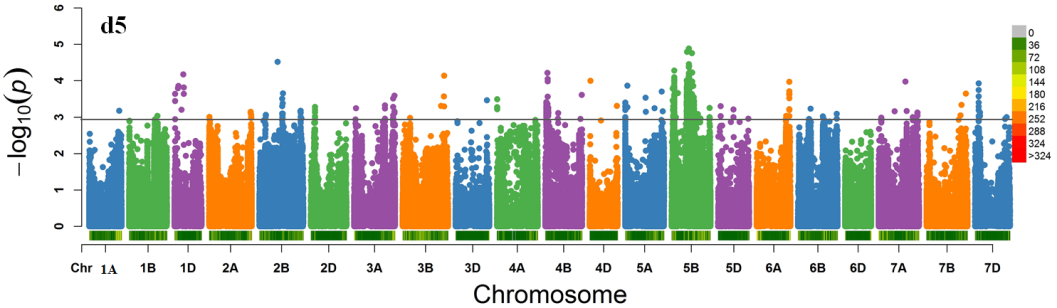

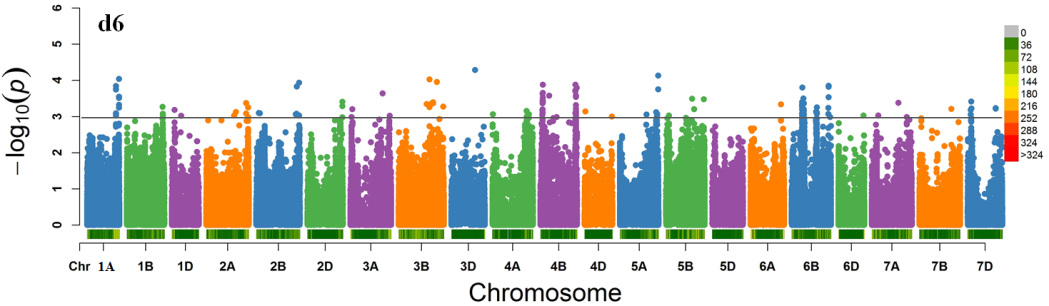

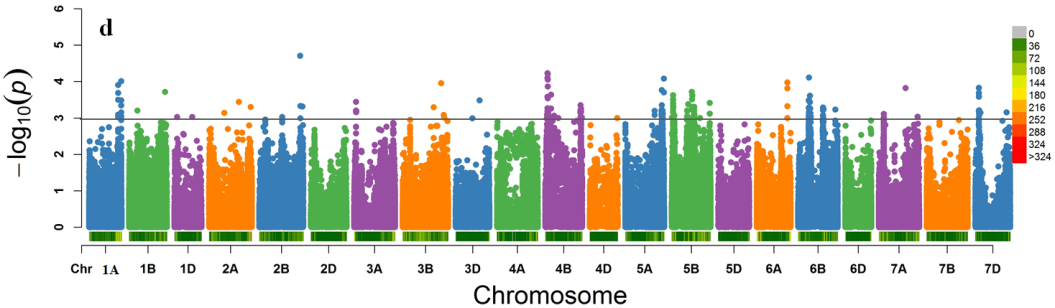


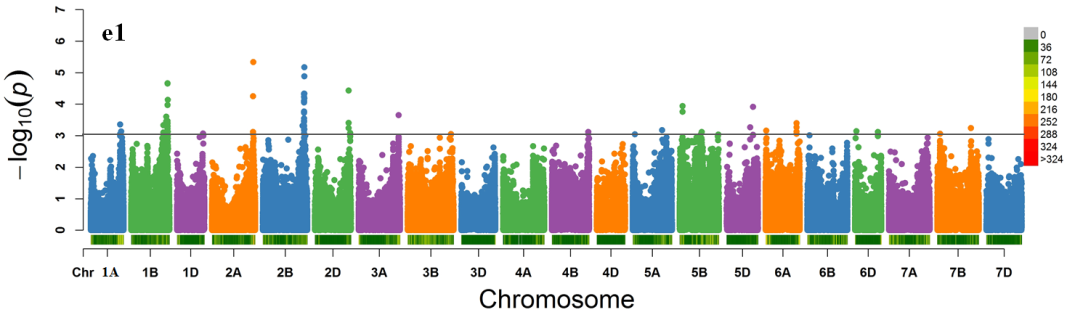

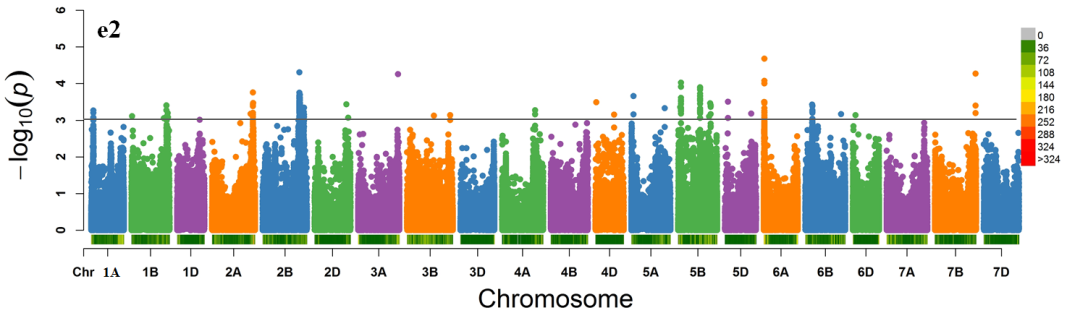

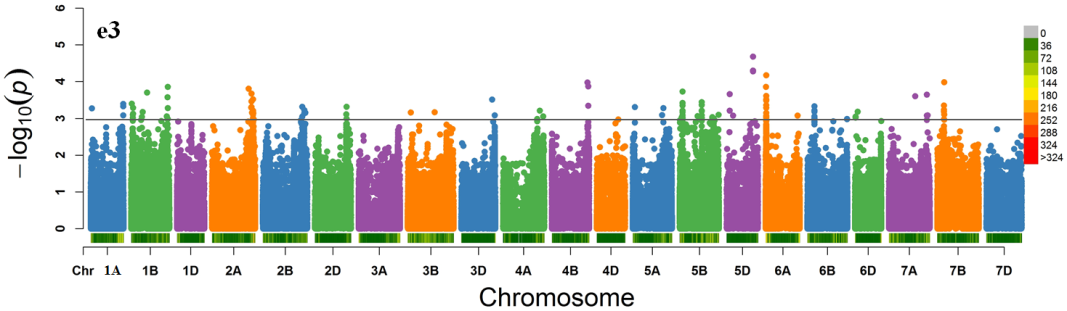

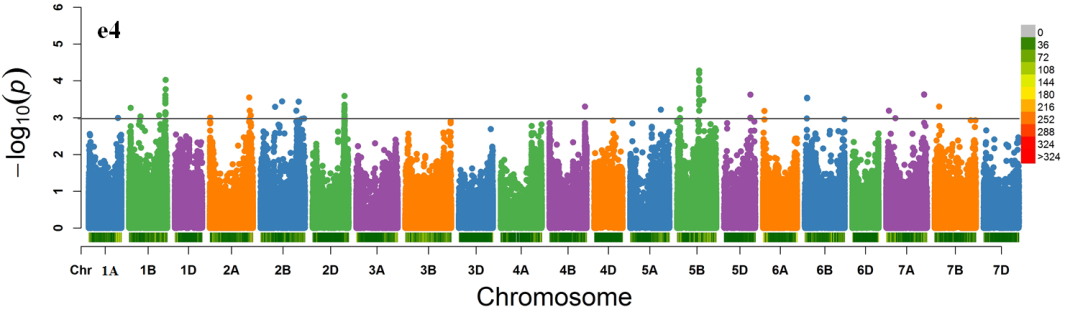

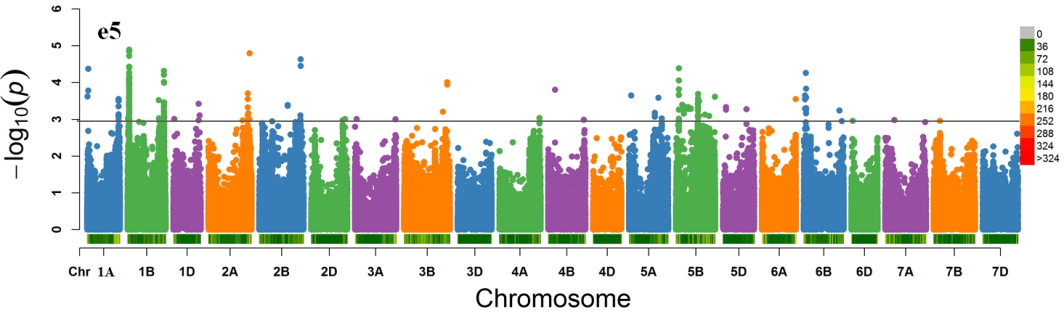

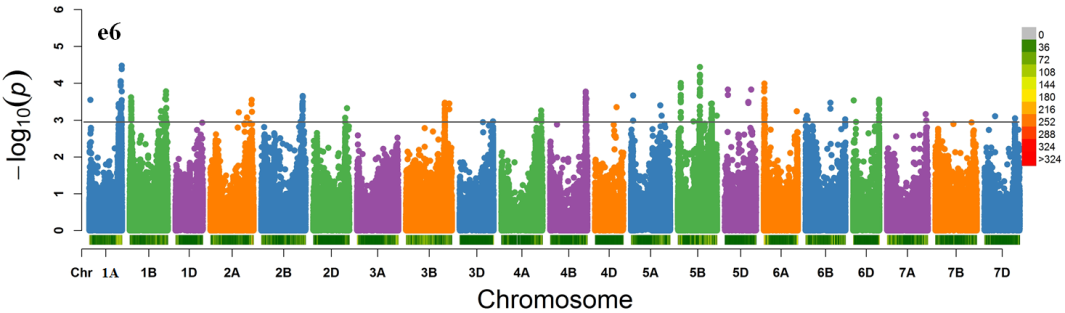

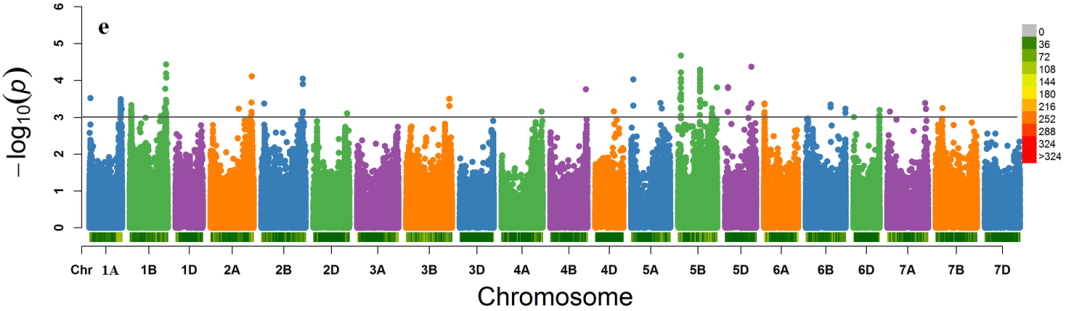


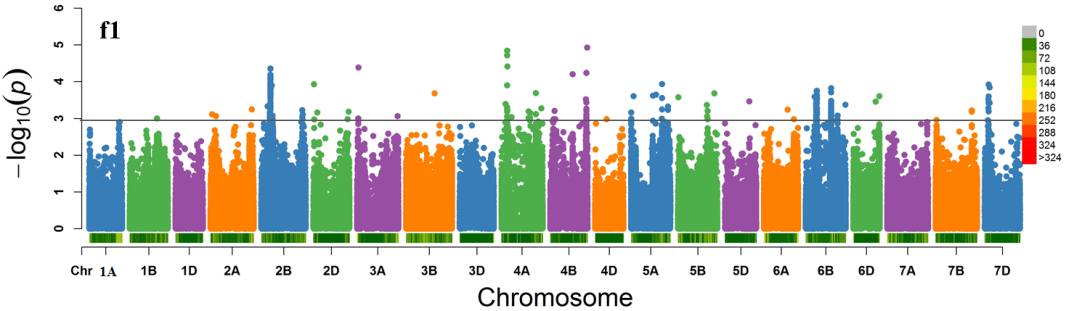

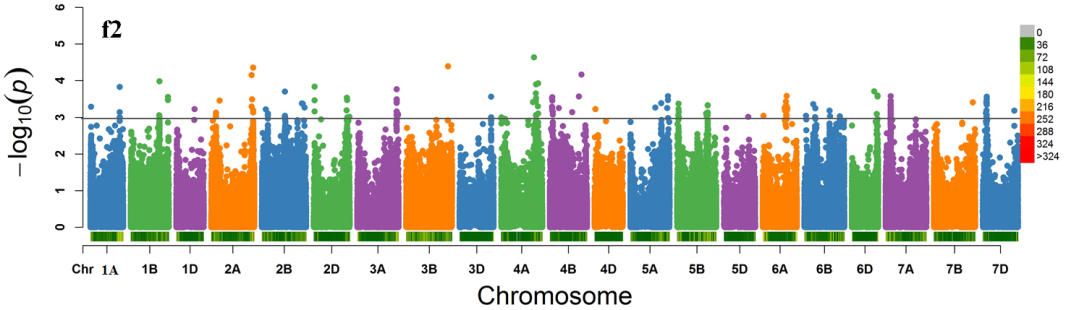

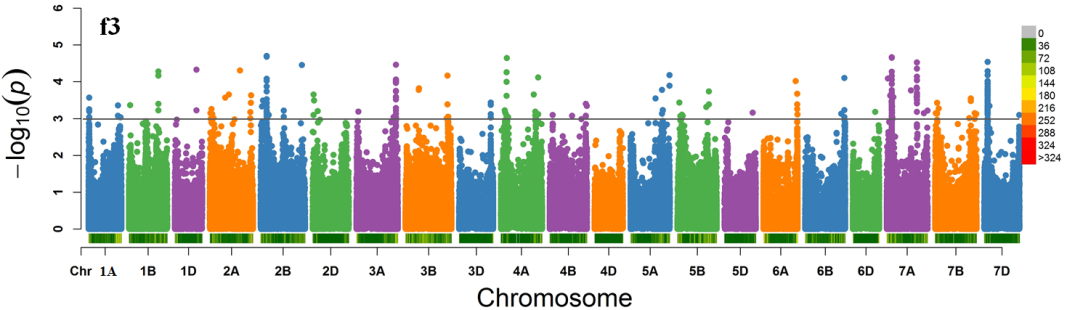

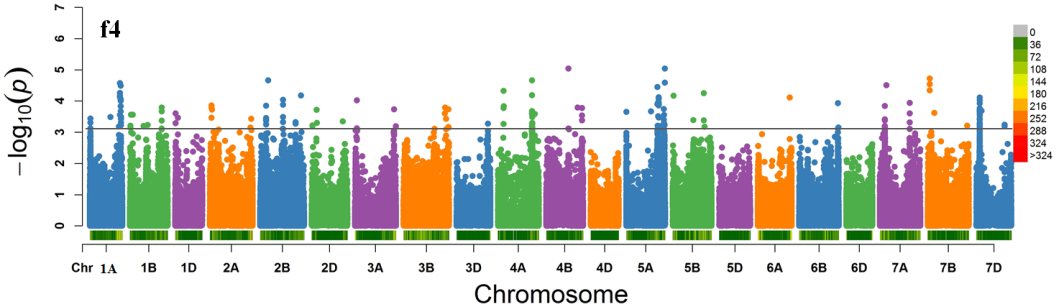

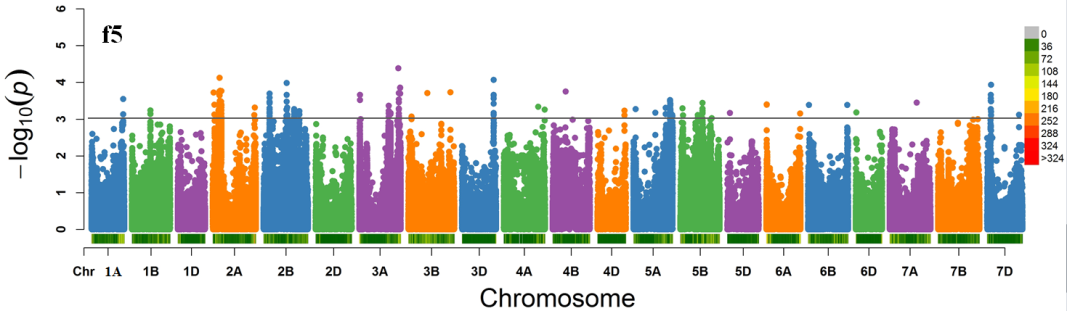

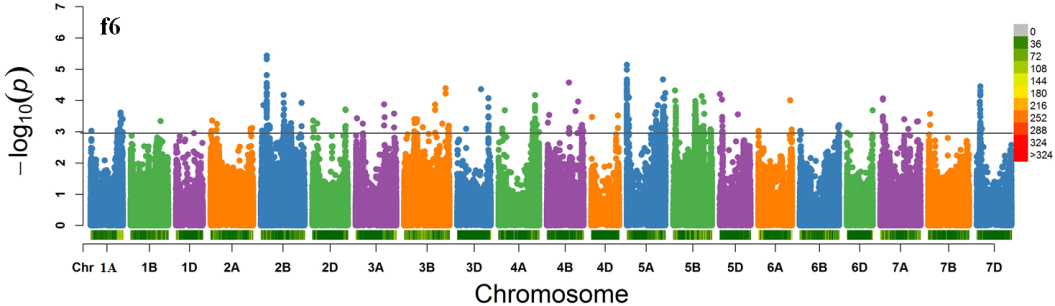

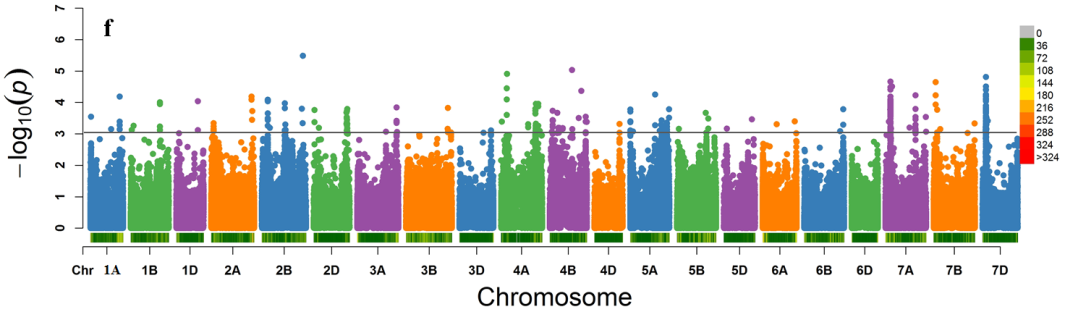


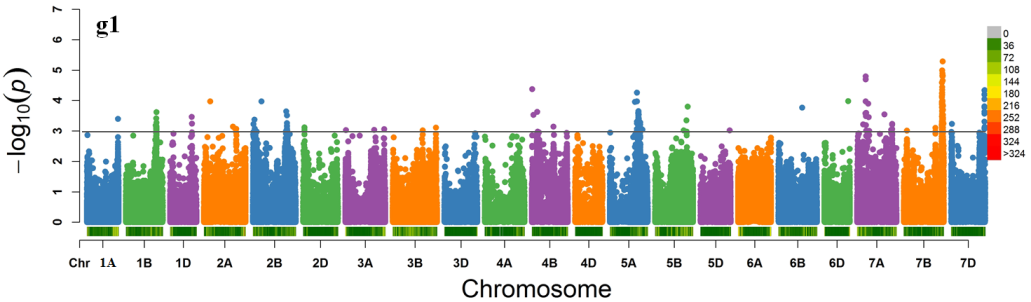

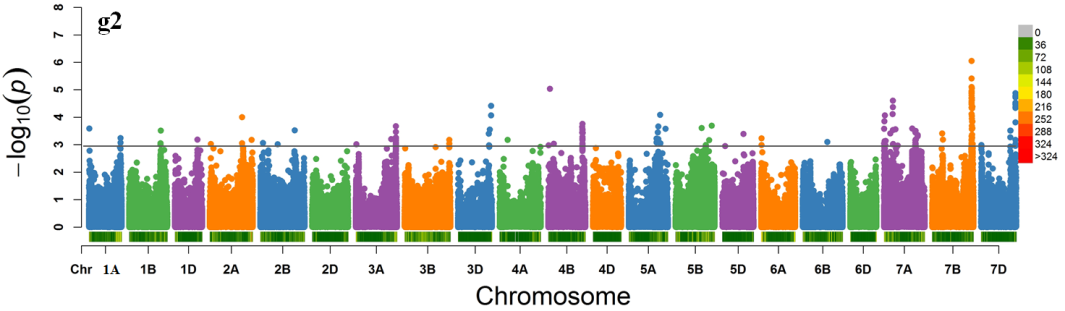

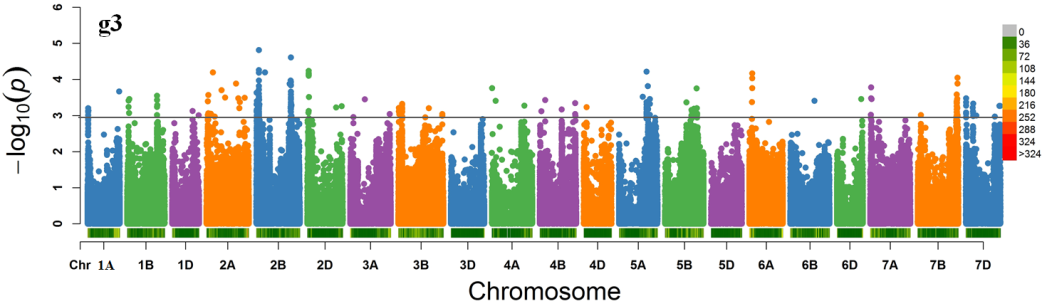

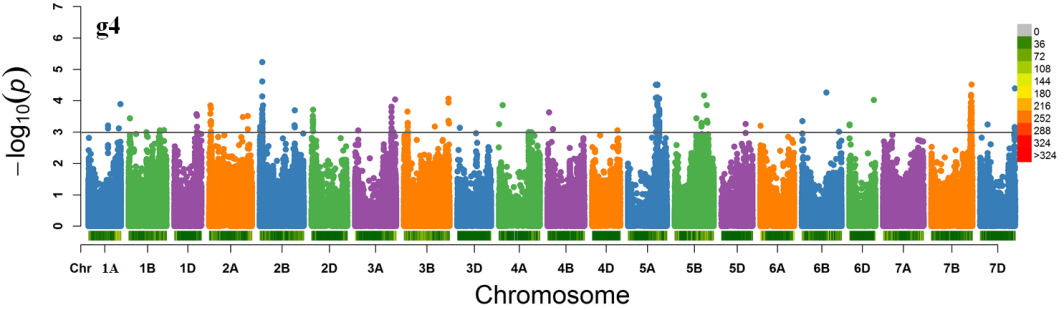

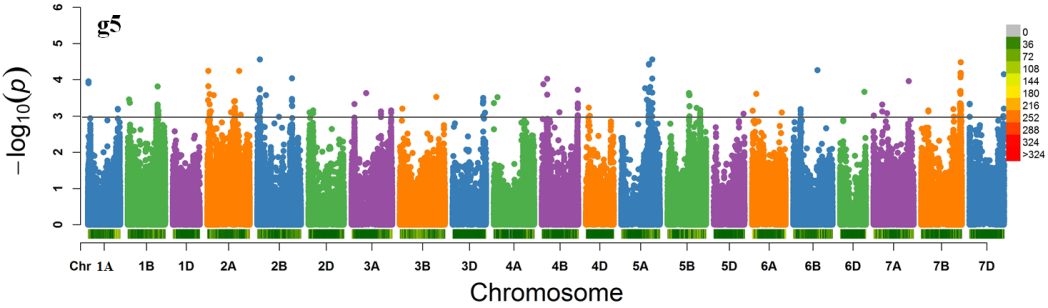

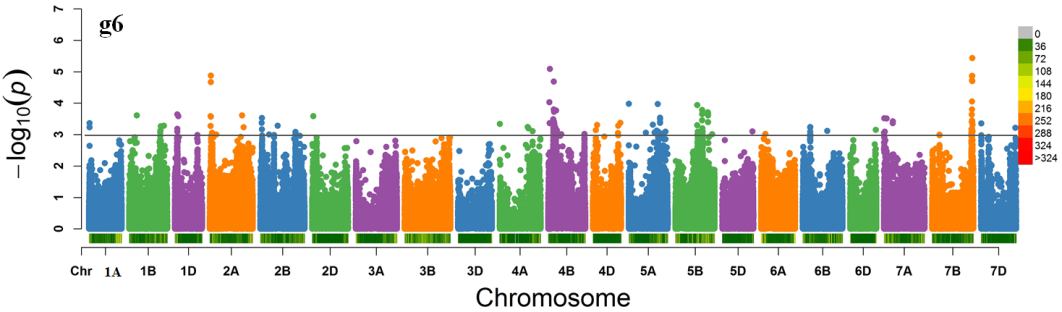

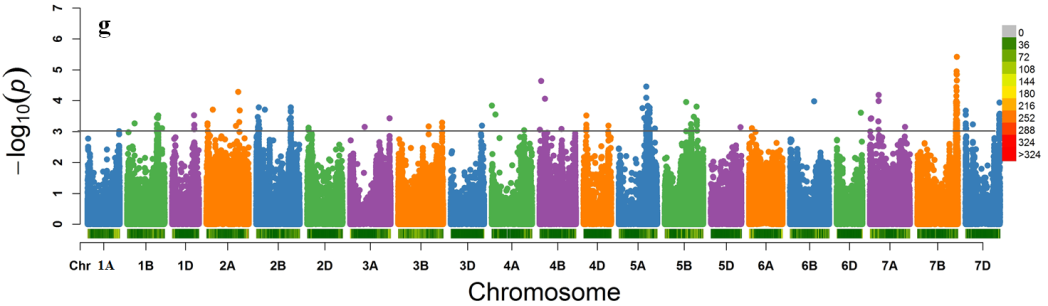


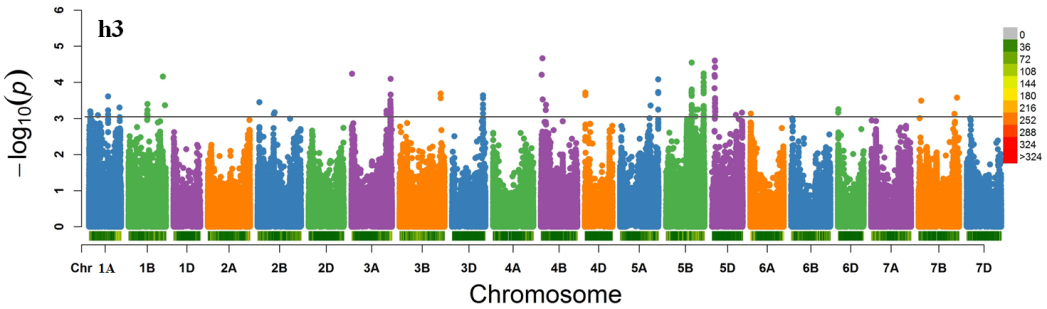

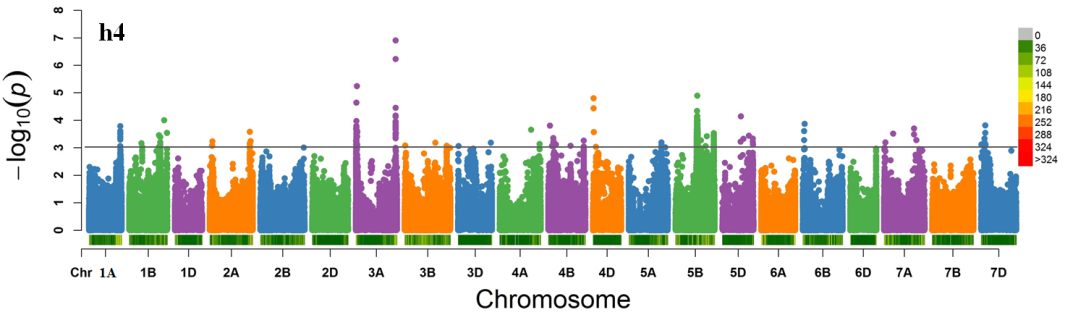

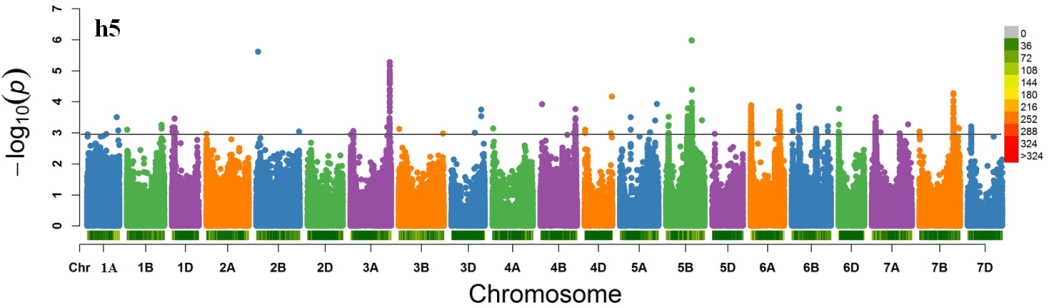

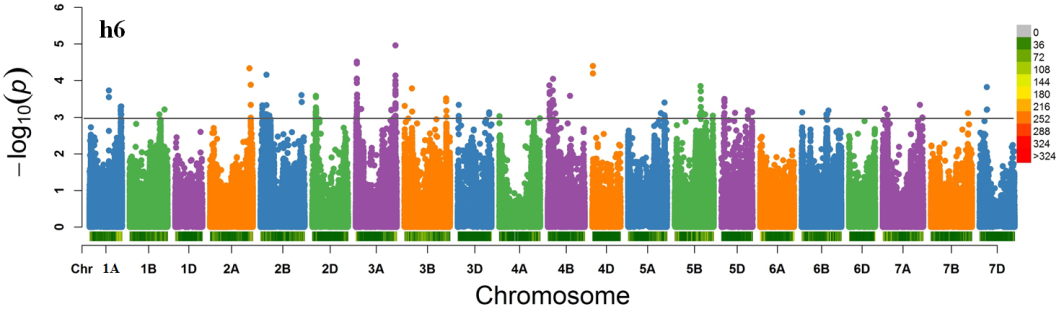

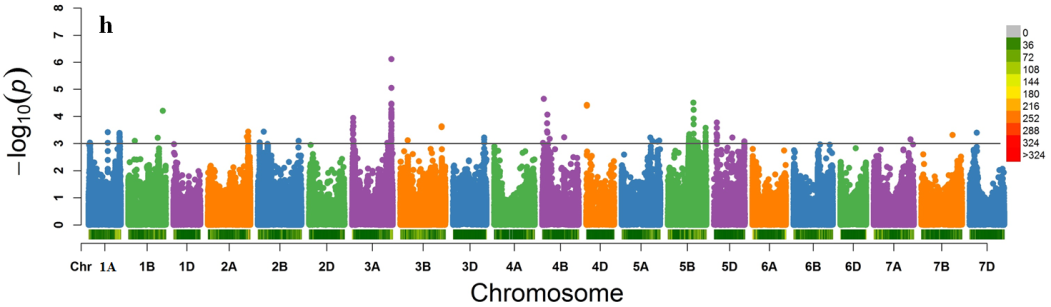


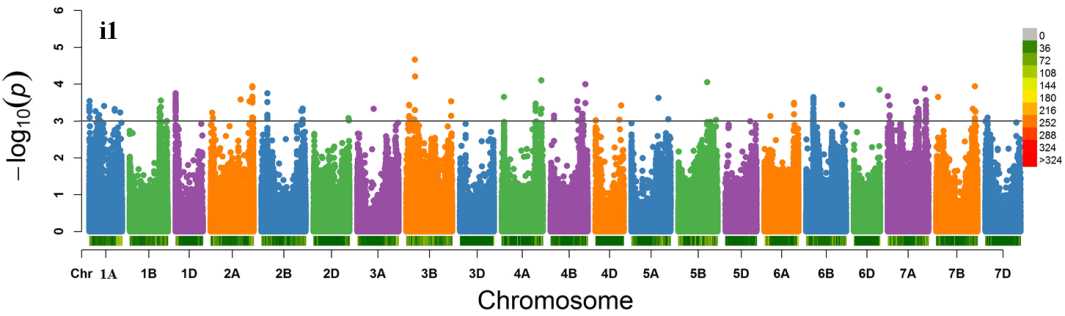

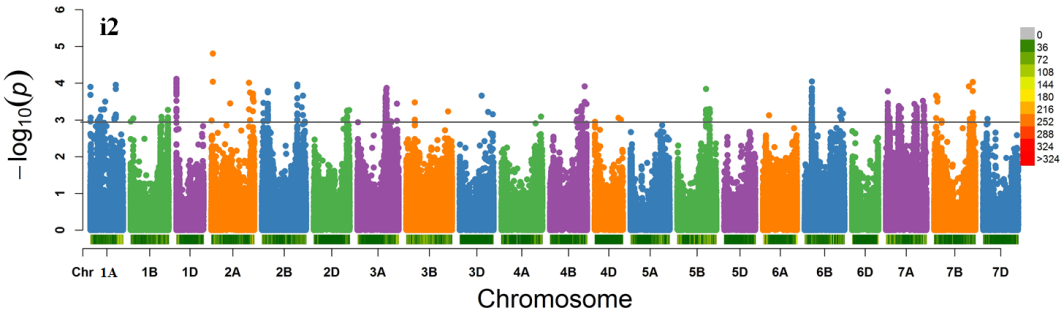

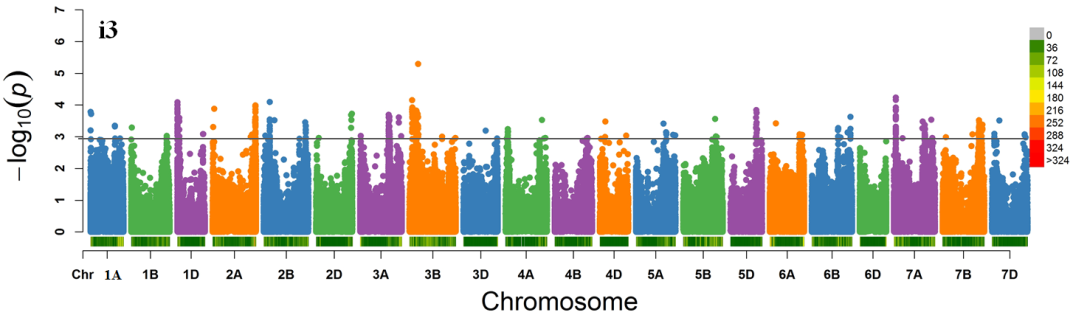

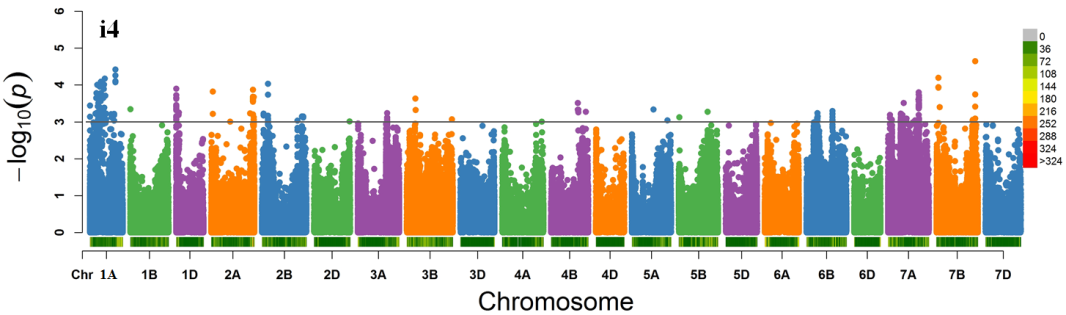

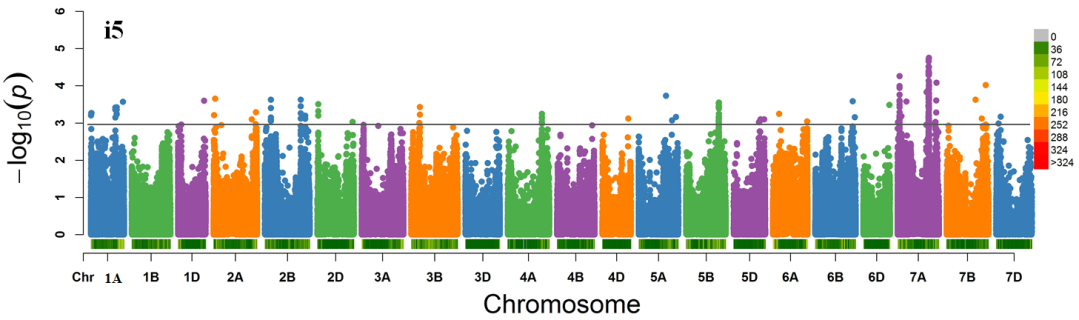

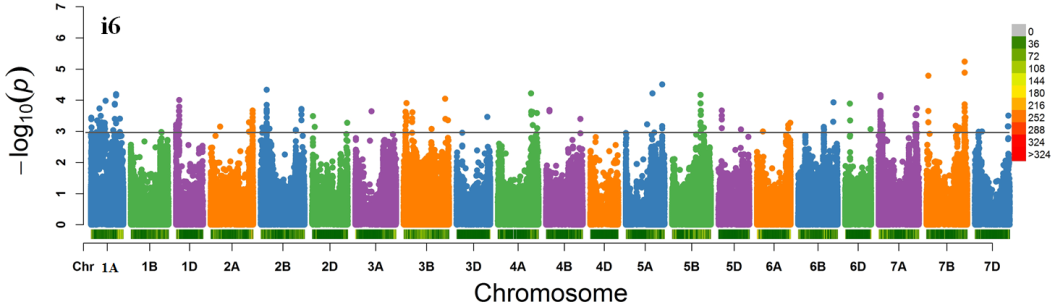

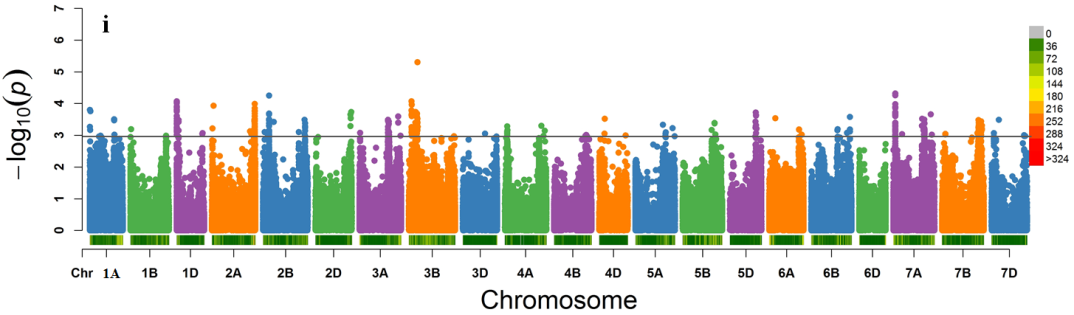


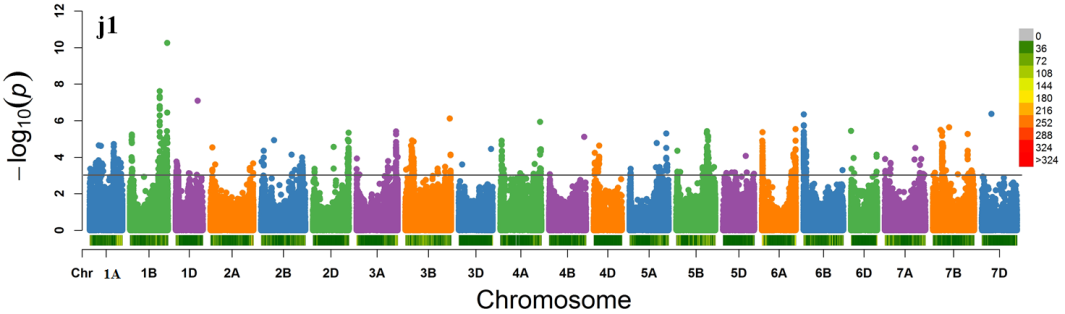

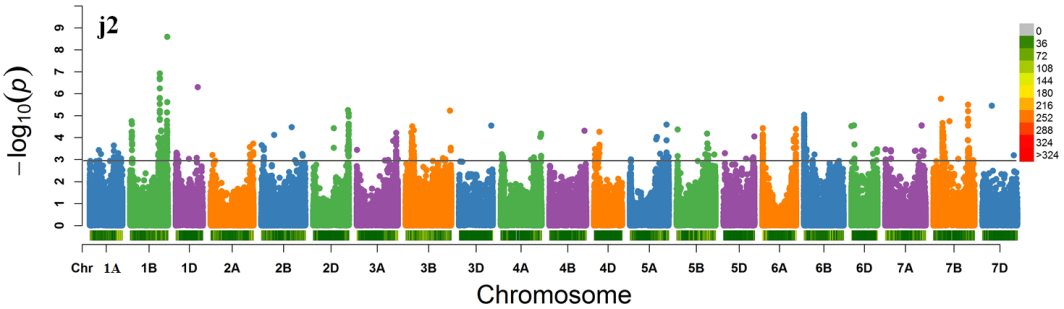

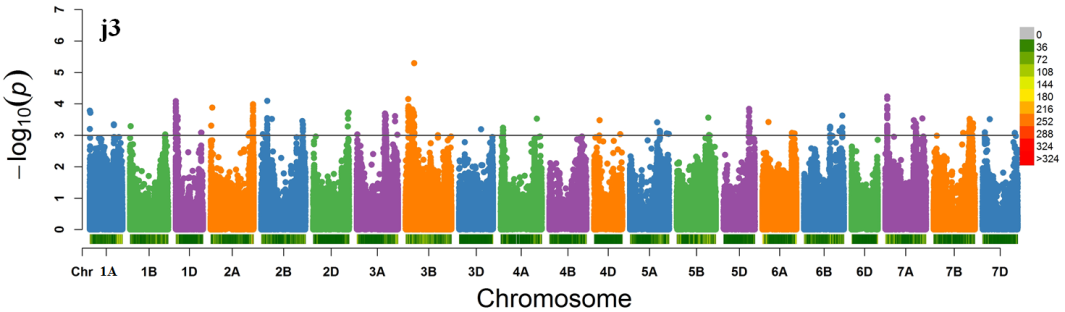

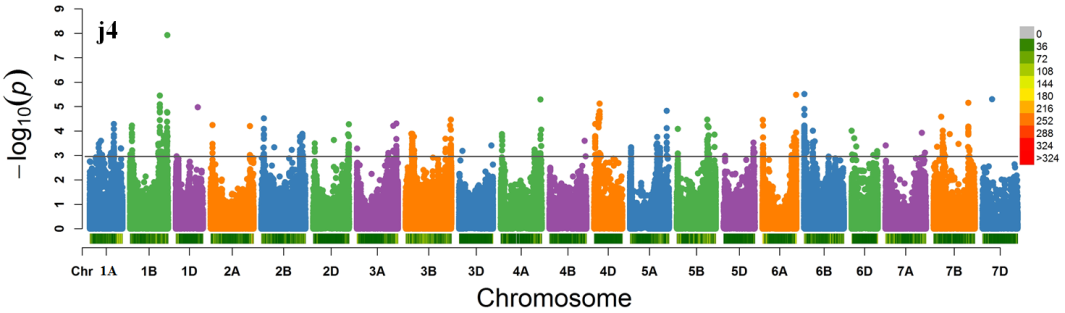

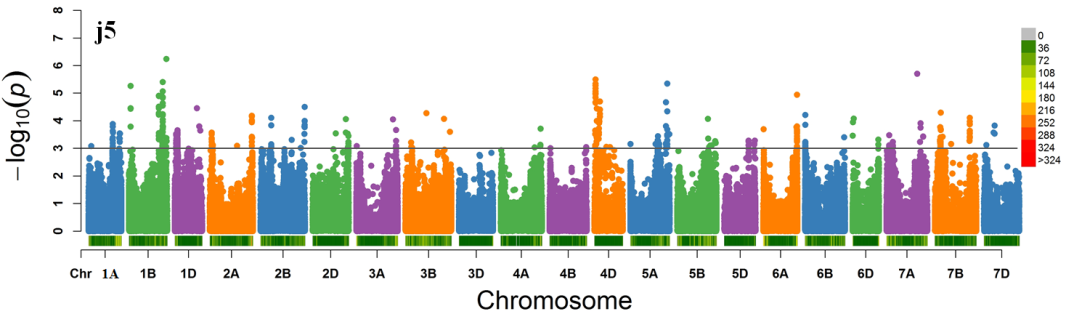

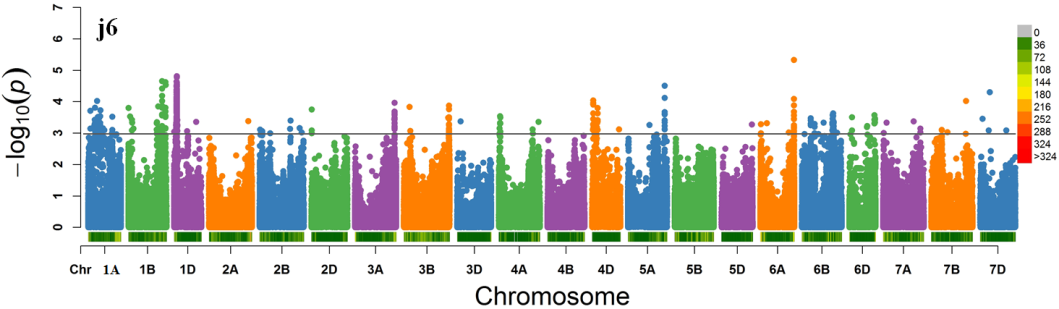

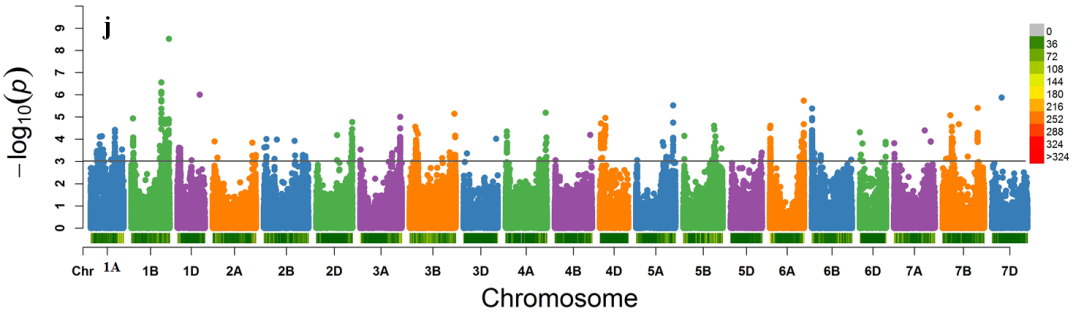

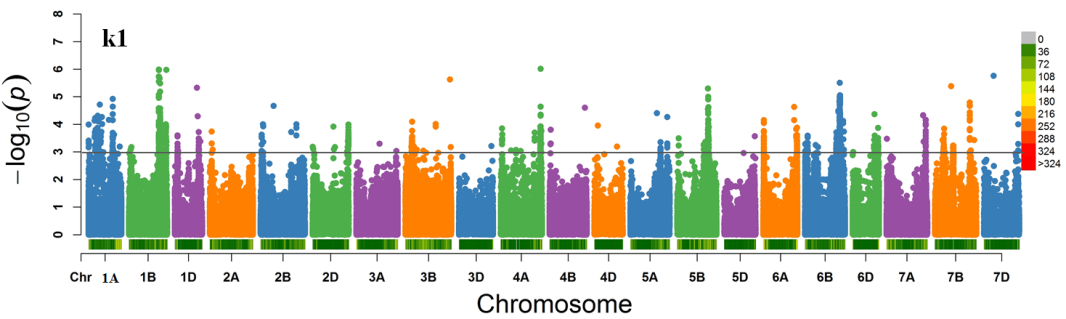

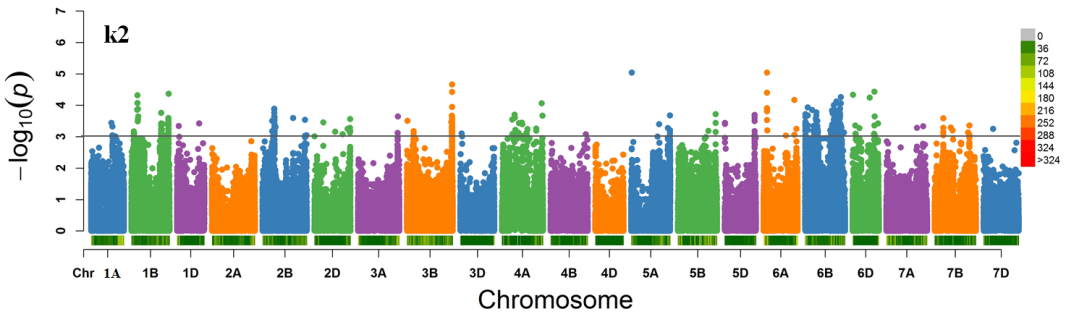

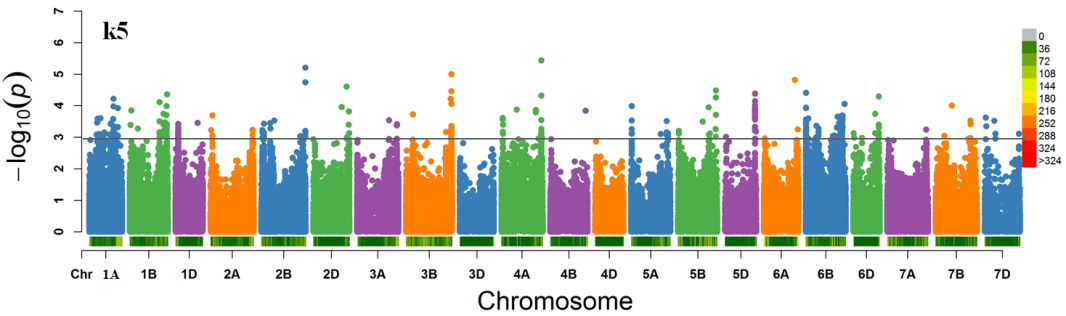

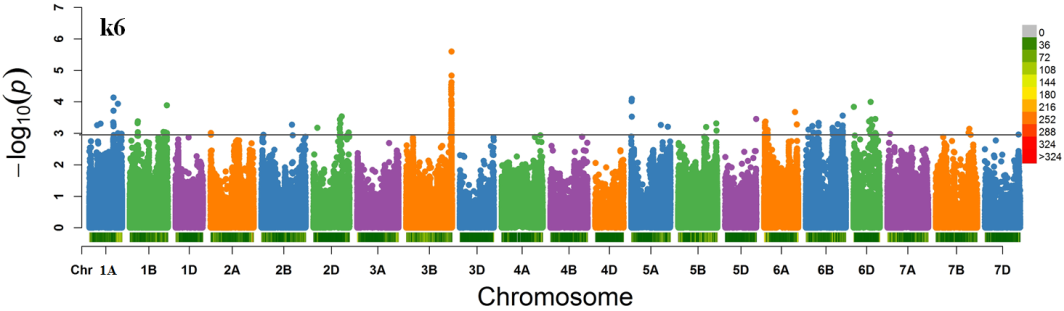

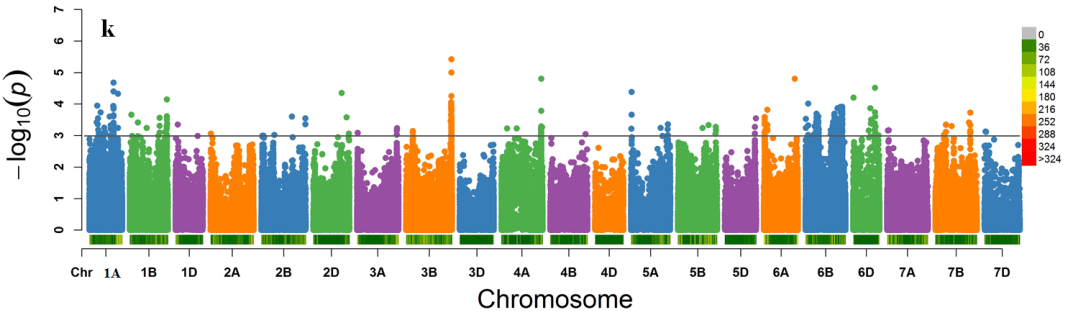


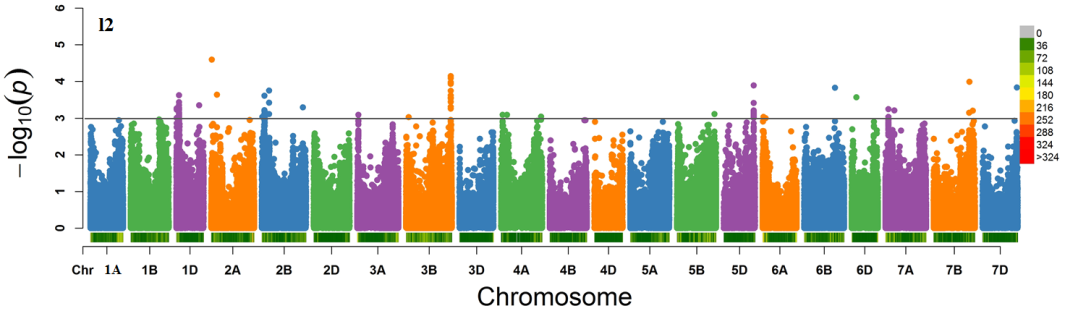

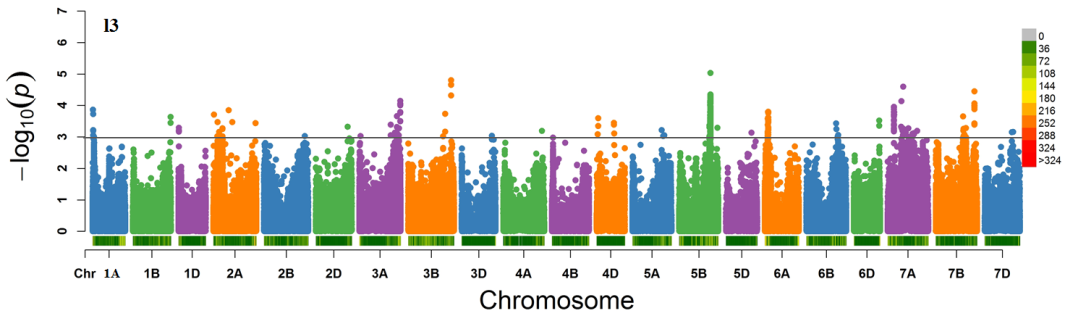

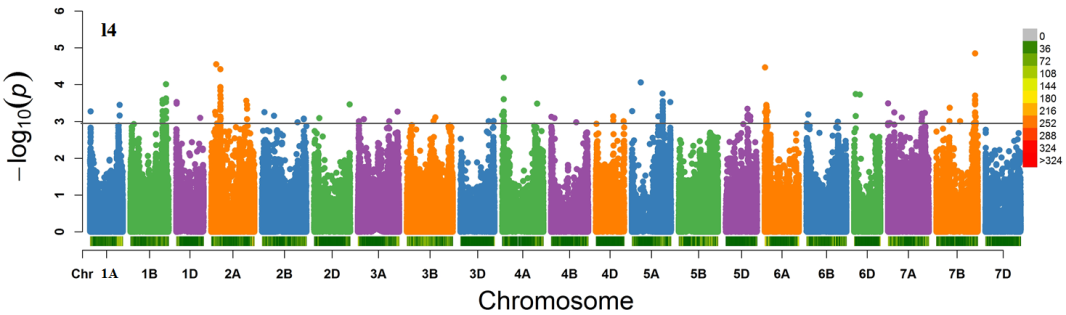

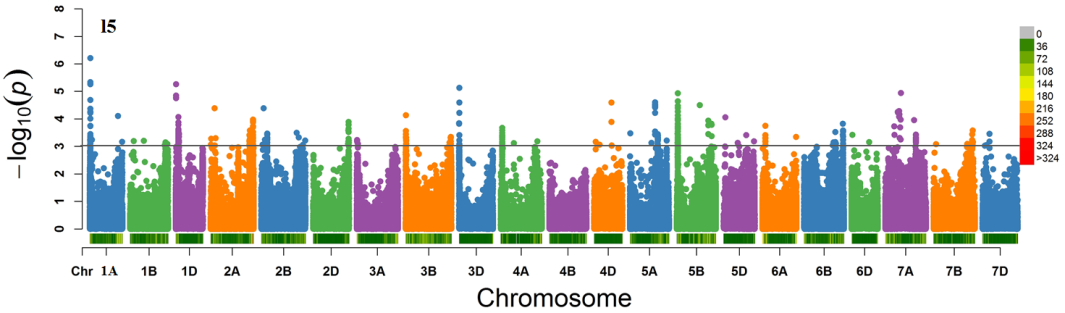

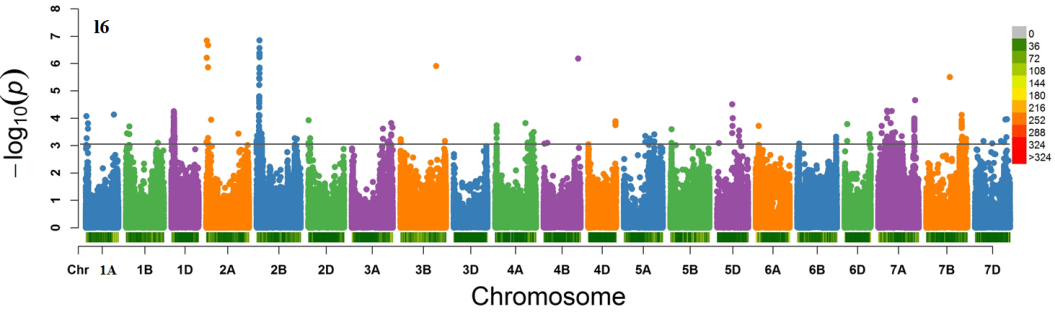

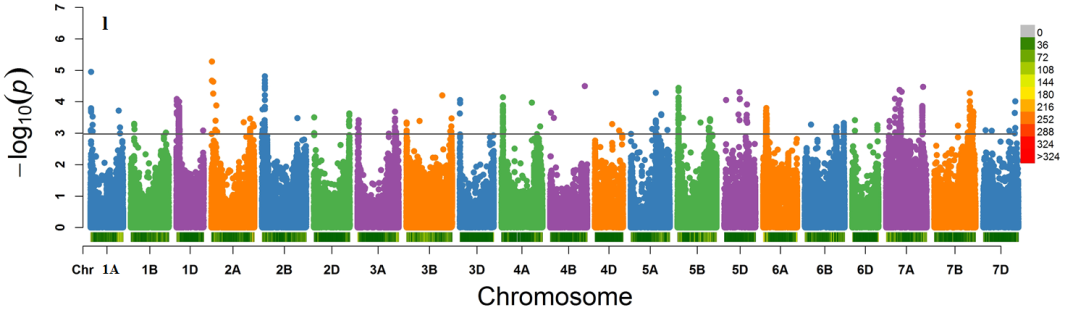


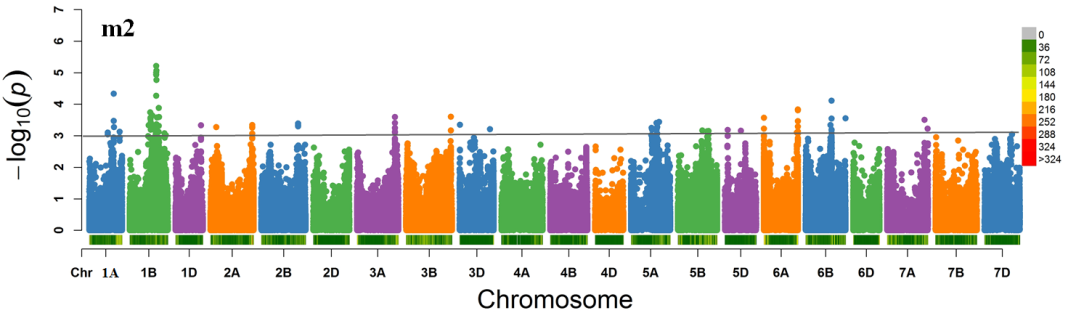

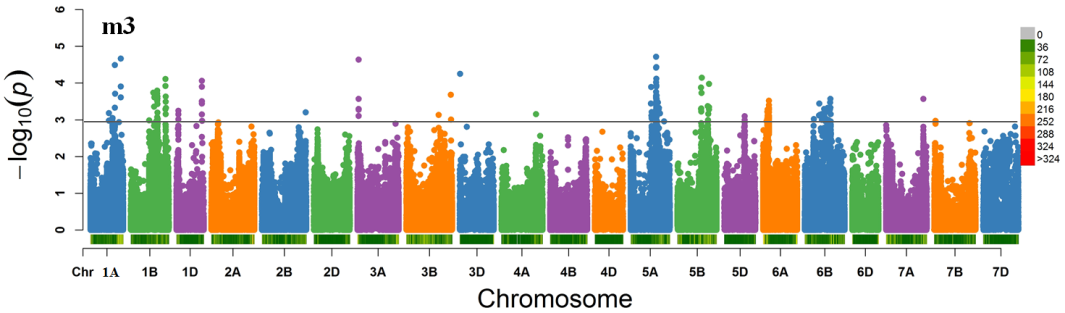

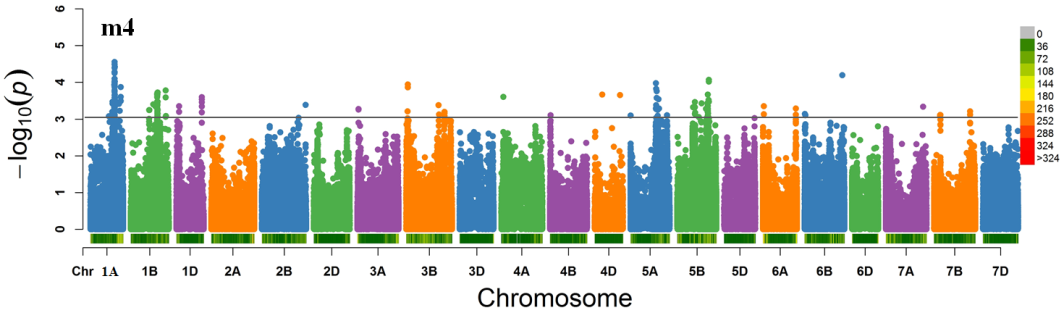

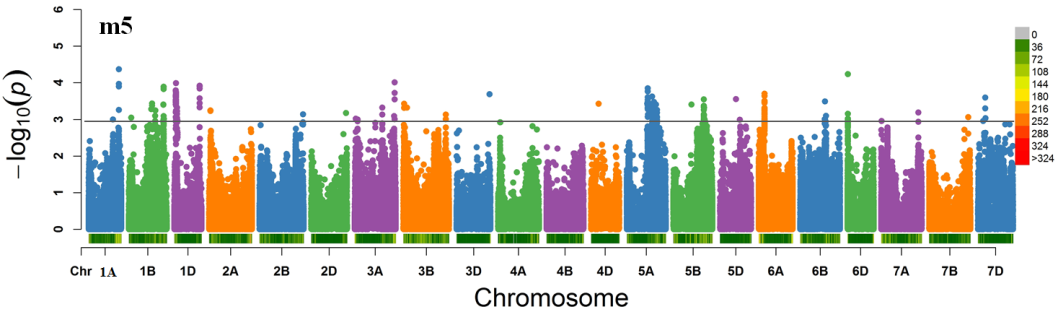

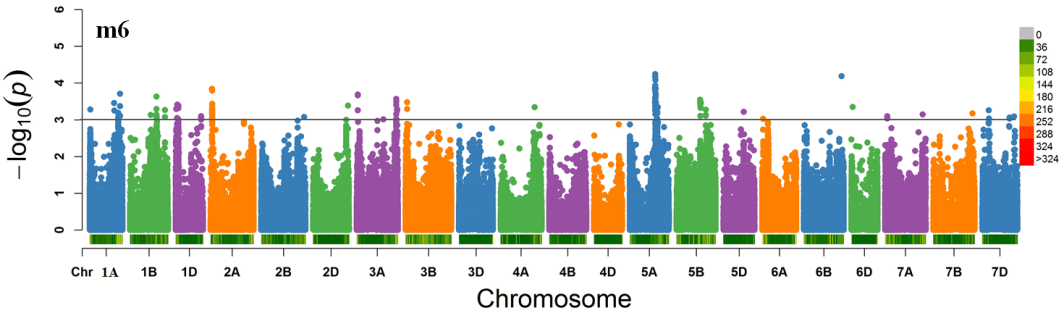

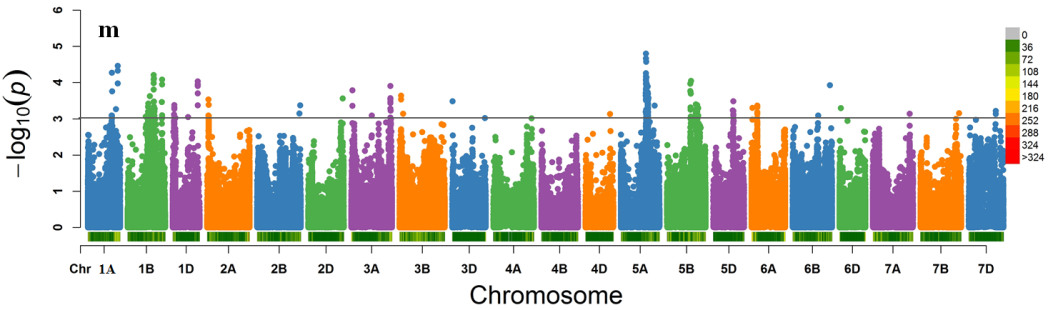


Fig. S3 Manhattan plots for grain yield and related traits in each environment and BLUE value in the diverse panel based on SNP-GWAS. a, grain yield; b, spike number per square meter; c, kernel number per spike; d, thousand-kernel weight; e, kernel length; f, kernel width; g, spike length; h, spike dry weight; i, heading date; j, plant height; k, uppermost internode length; l, flag leaf length; m, flag leaf width; 1, 2012–2013 Anyang; 2, 2012–2013 Suixi; 3, 2013–2014 Anyang; 4, 2013–2014 Suixi; 5, 2014–2015 Anyang; 6, 2014–2015 Shijiazhuang
