# Supplementary material for: Genetic architecture of grain yield in bread wheat based on genome-wide association studies
Source: BMC Plant Biol. 2019 Apr 29;19:168. doi: 10.1186/s12870-019-1781-3 (PMC6489268; doi:10.1186/s12870-019-1781-3)
Supplement: Supplementary file 10 — Figure S4. Manhattan plots for grain yield and related traits in each environment and BLUE value in the diverse panel based on Haplotype-GWAS. See footnote to Fig. S3 for traits and experimental sites. (DOCX 13093 kb) [file 12870_2019_1781_MOESM10_ESM.docx]

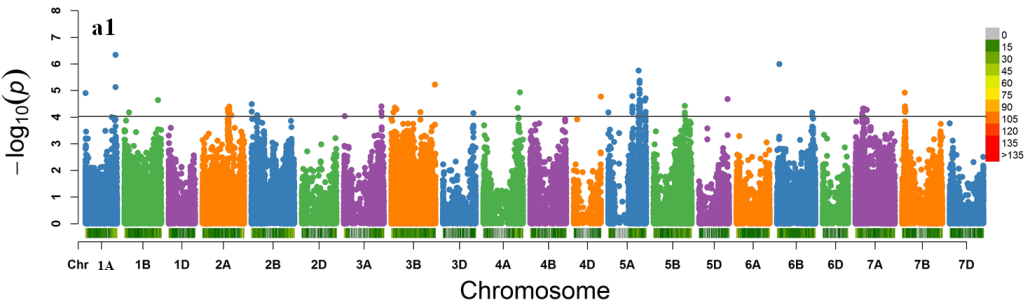

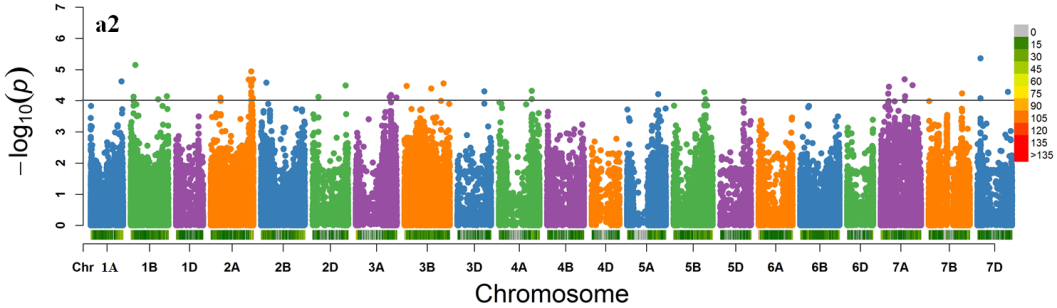

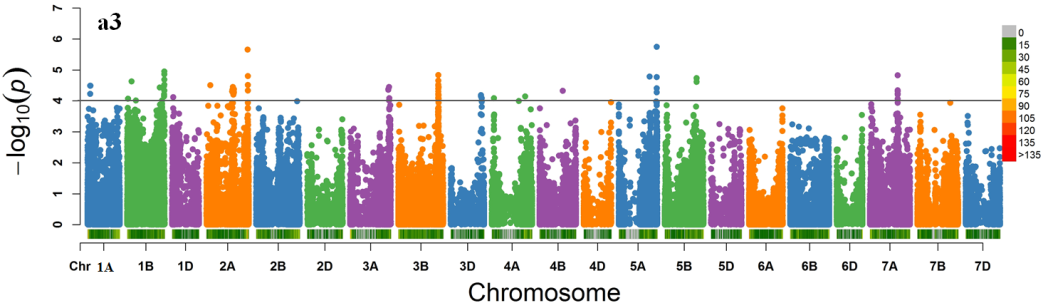

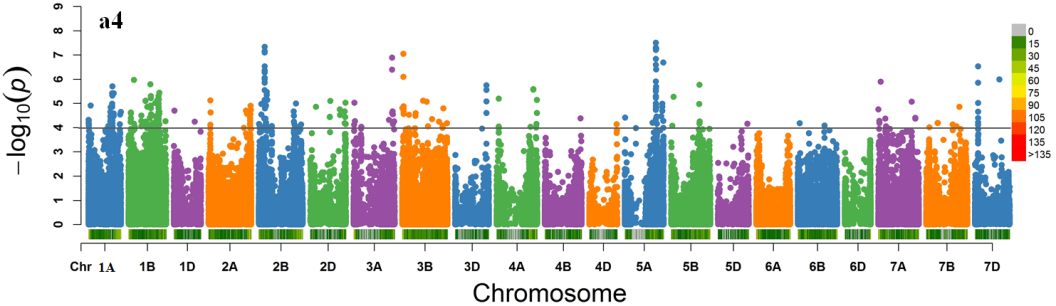

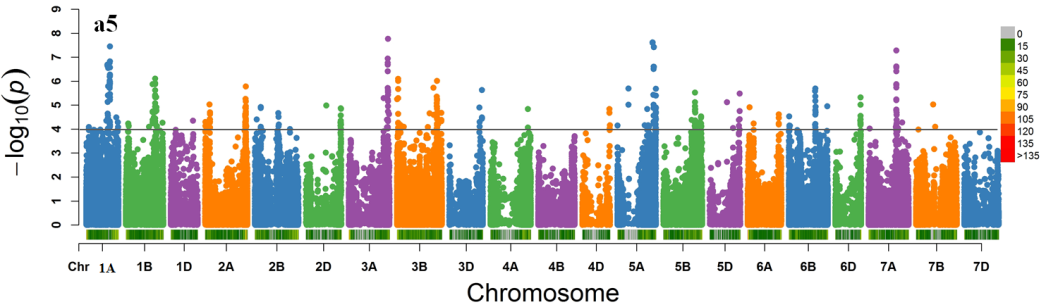

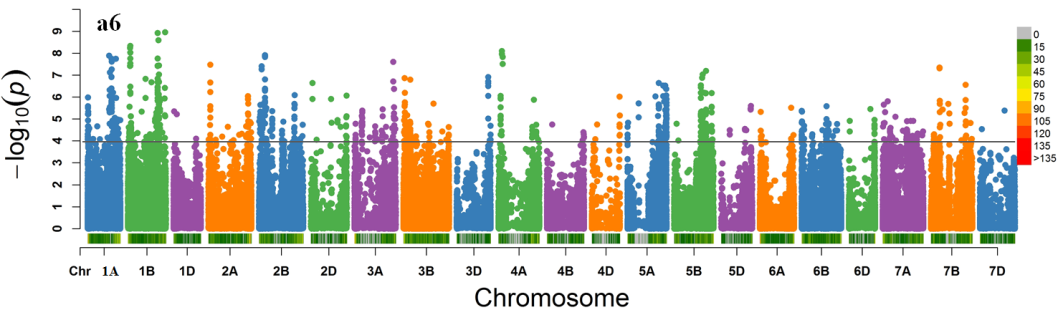

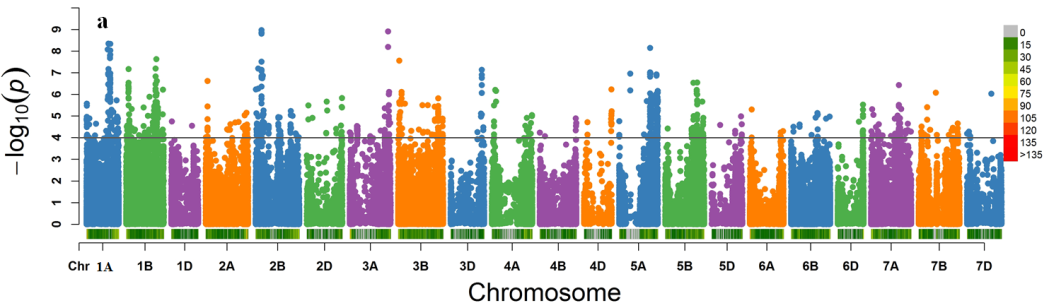


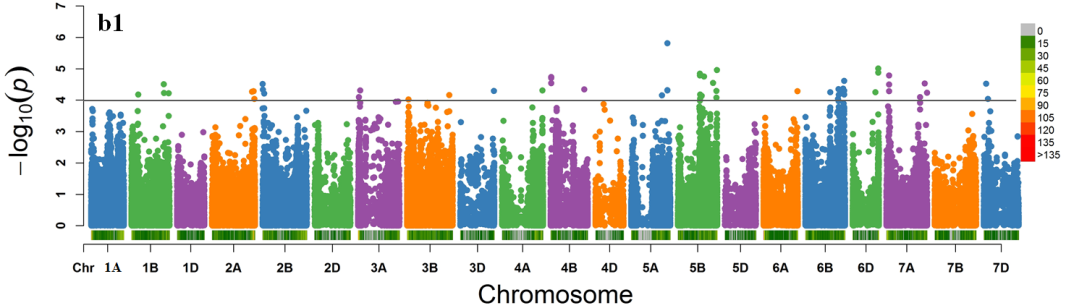

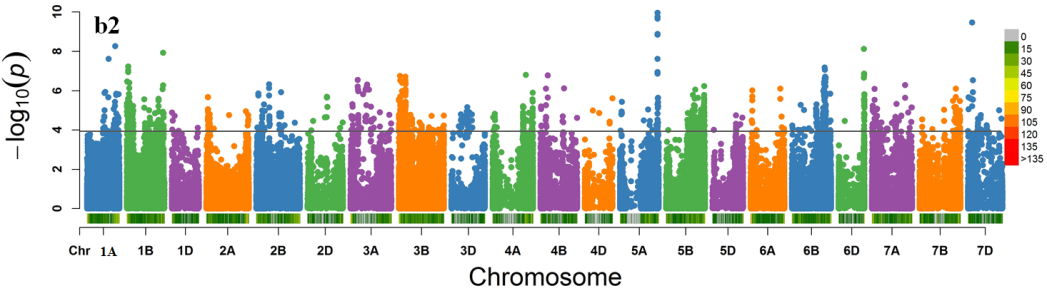

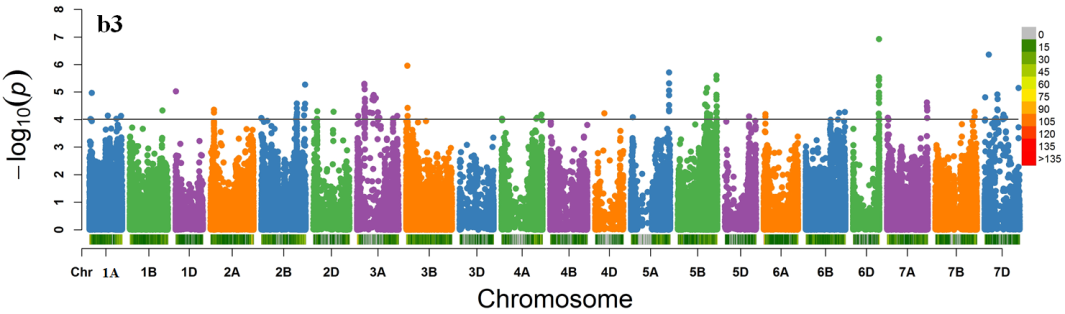

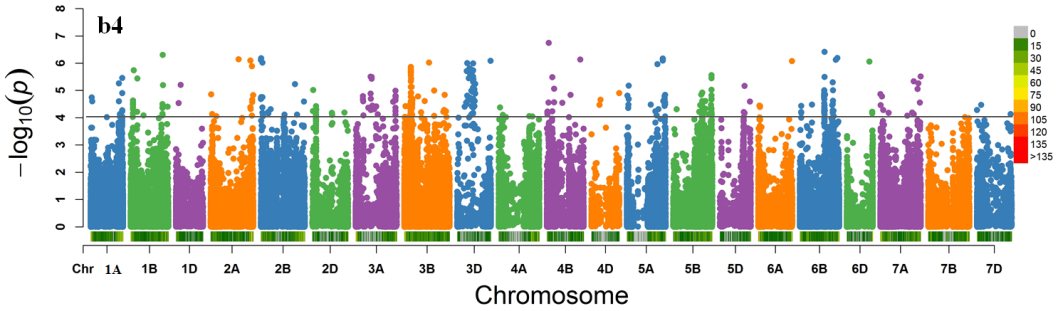

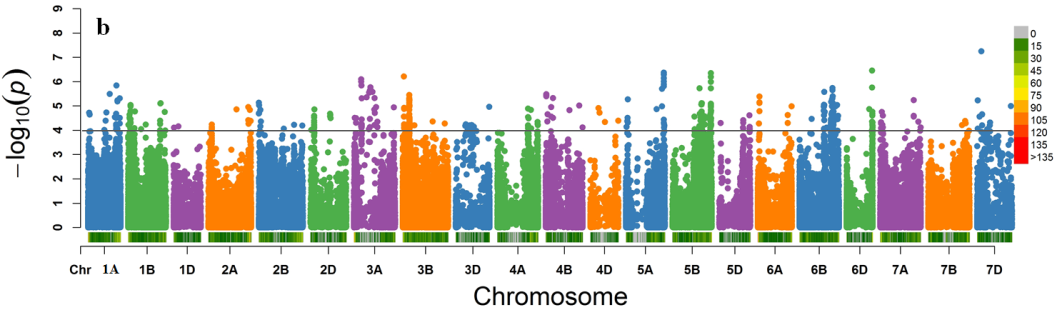


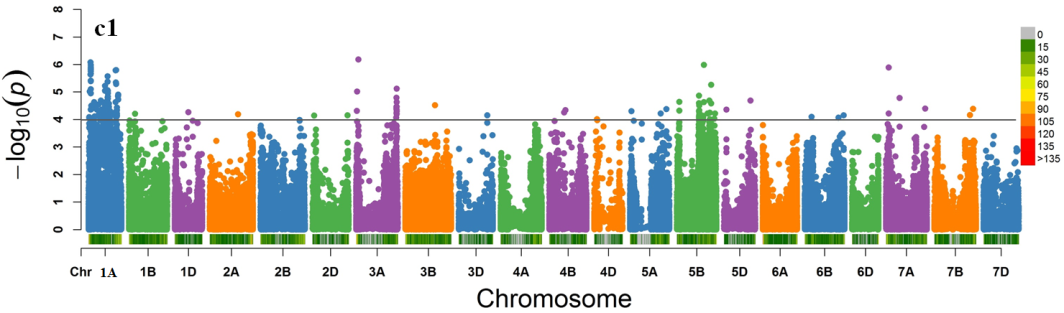

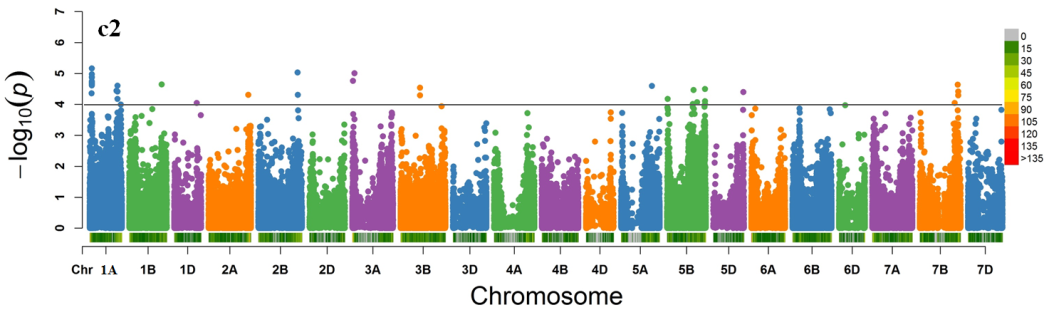

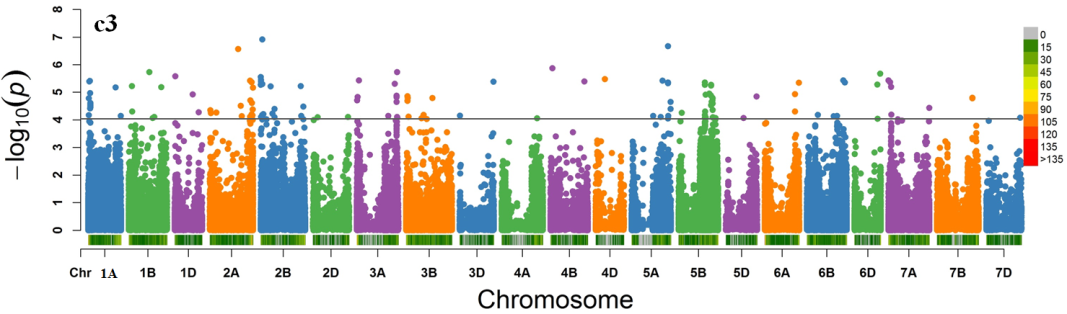

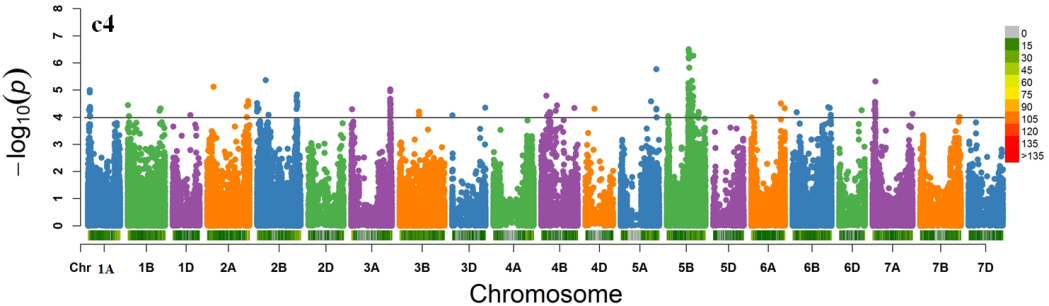

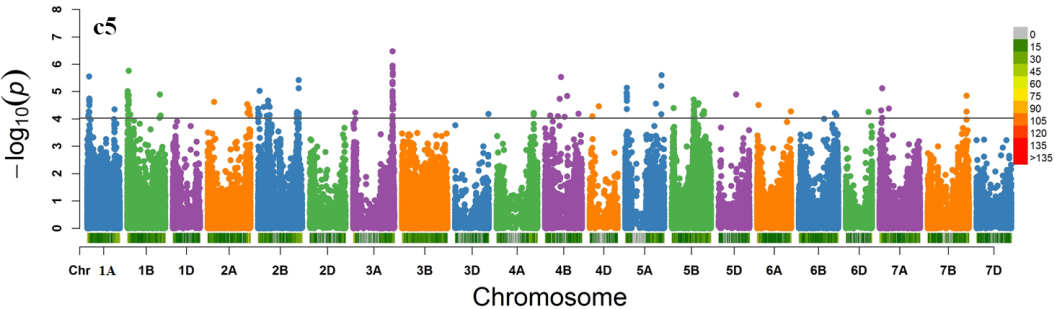

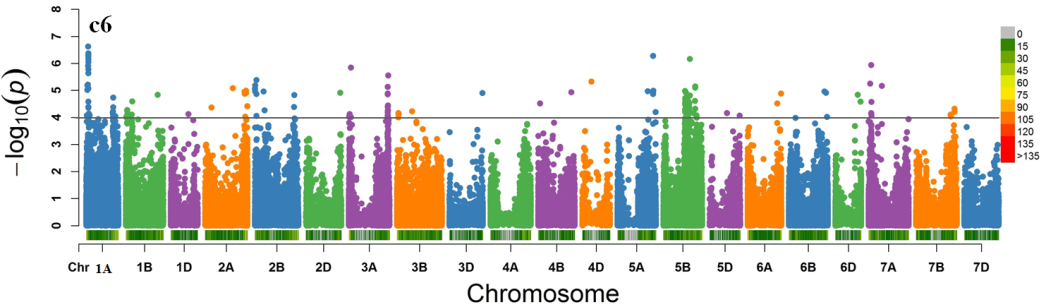

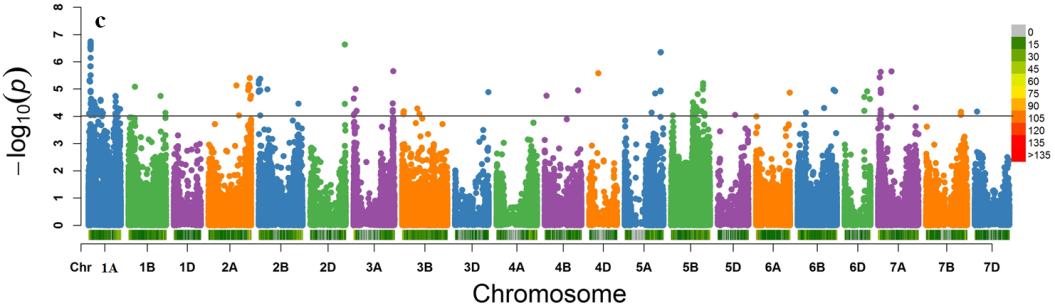


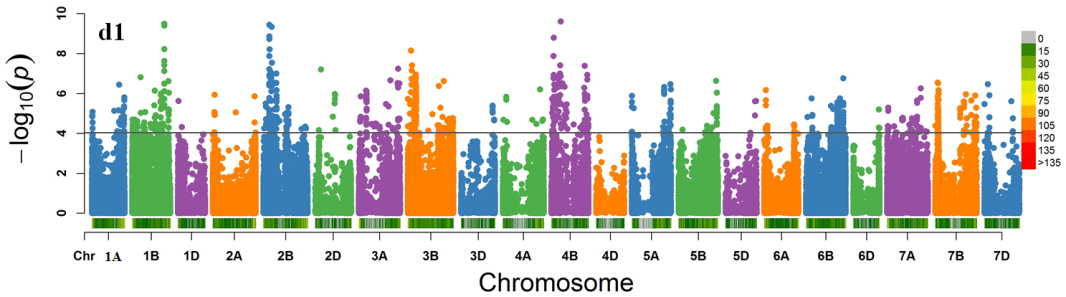

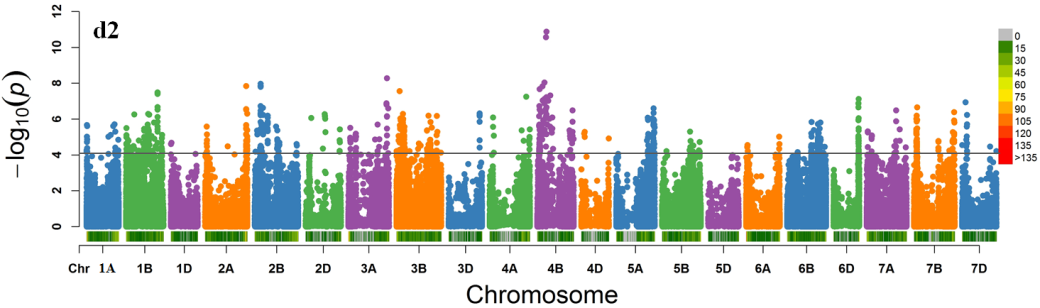

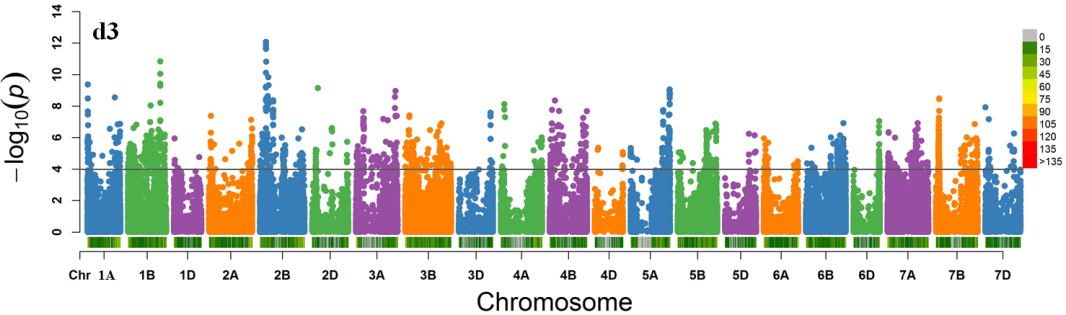

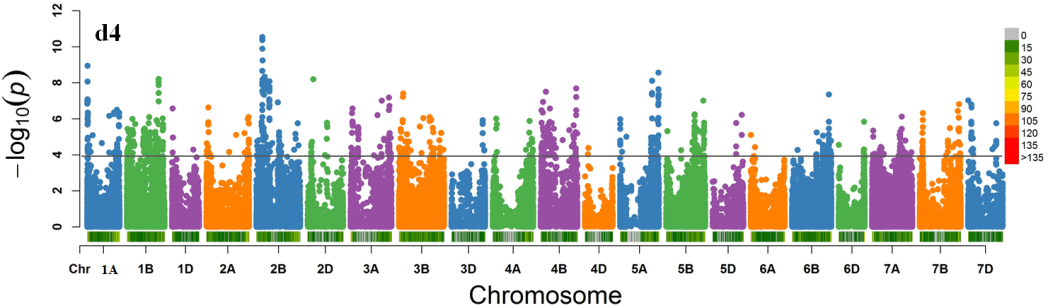

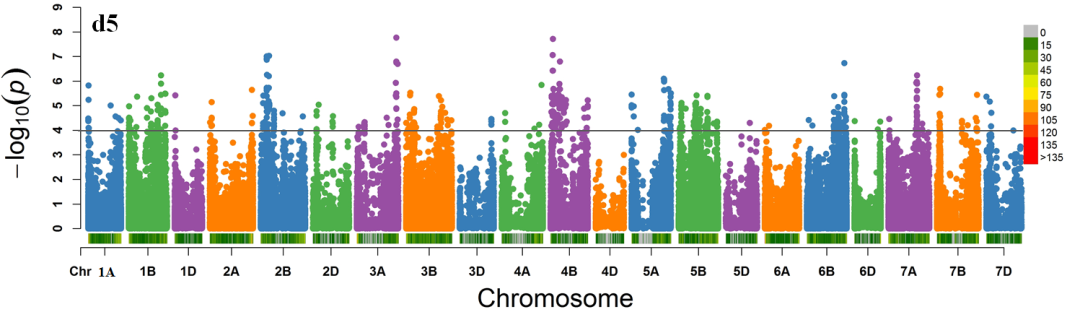

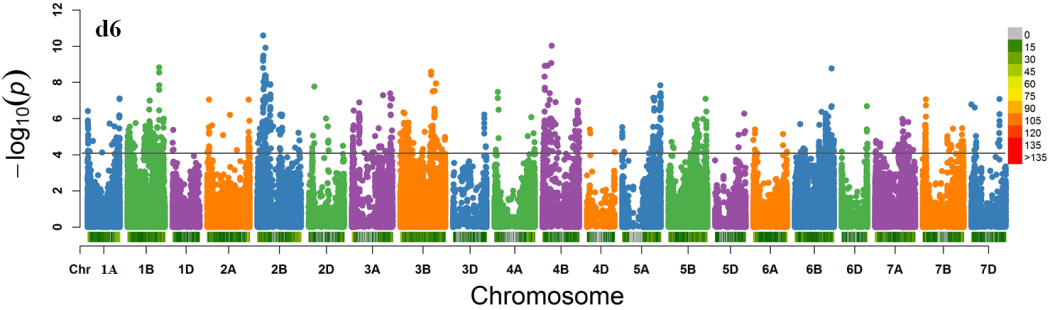

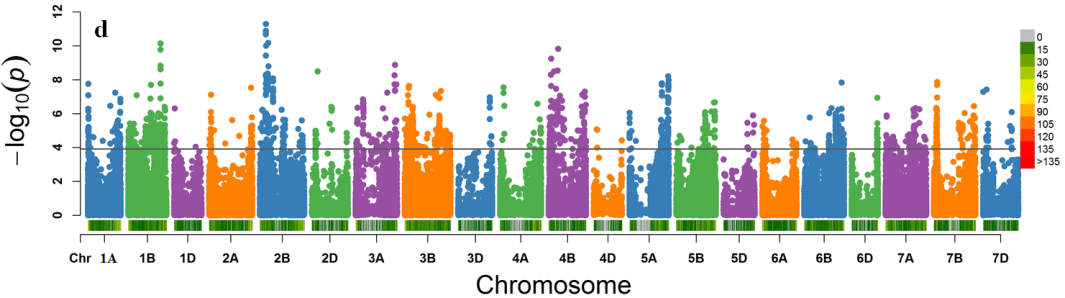


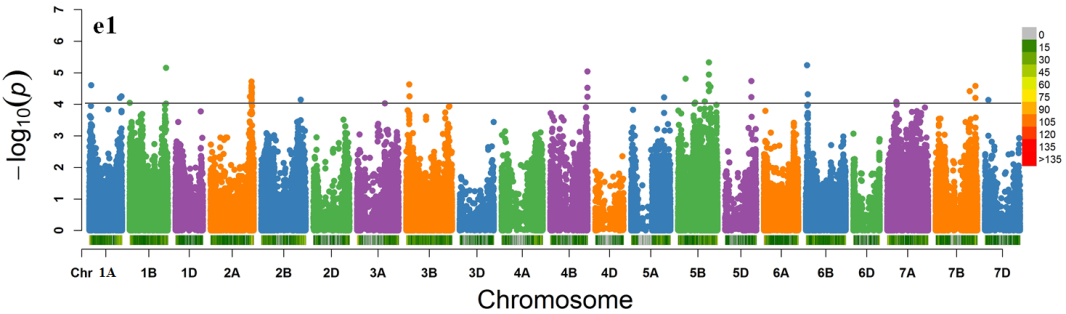

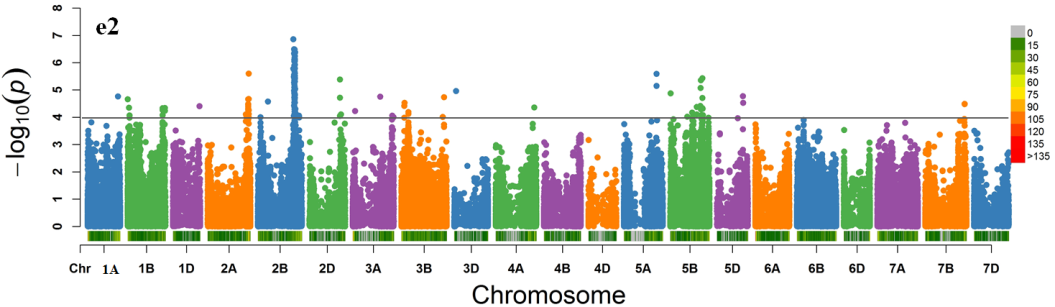

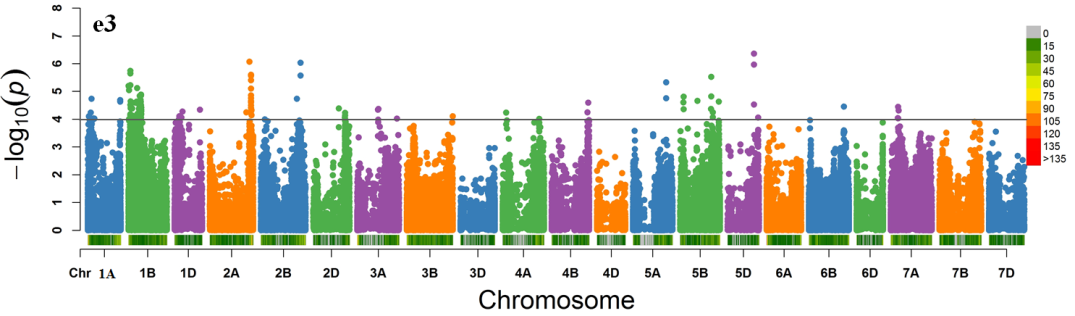

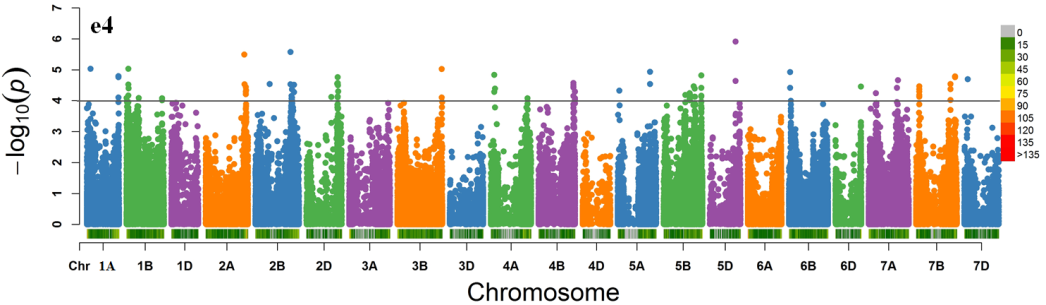

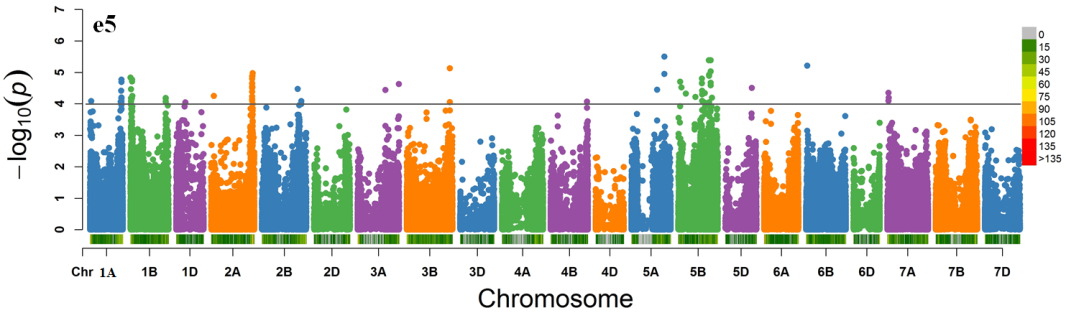

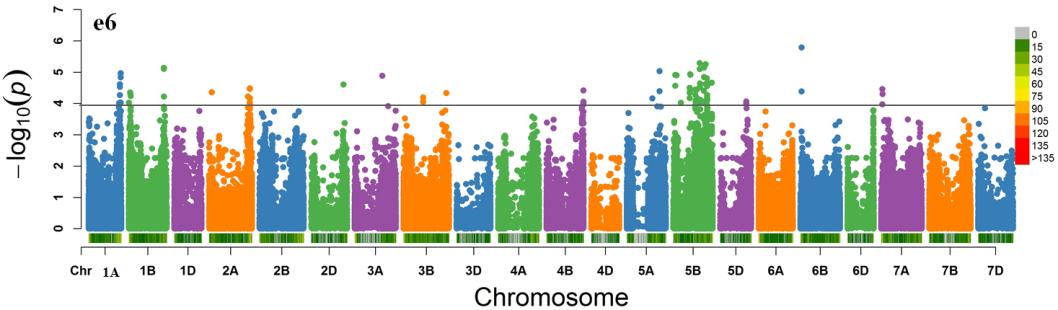

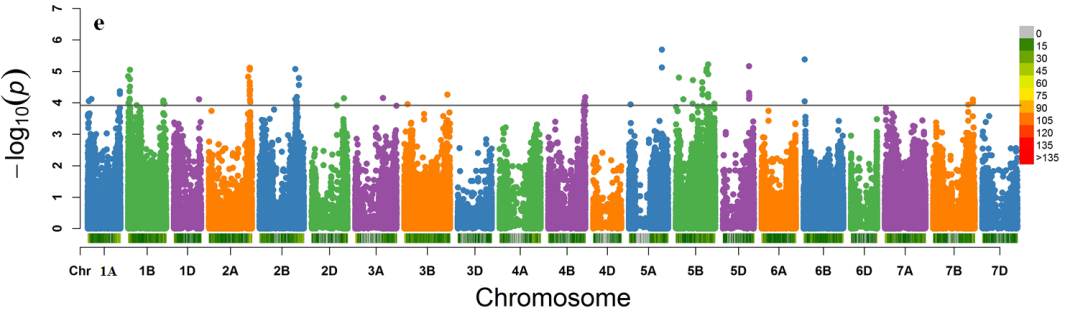


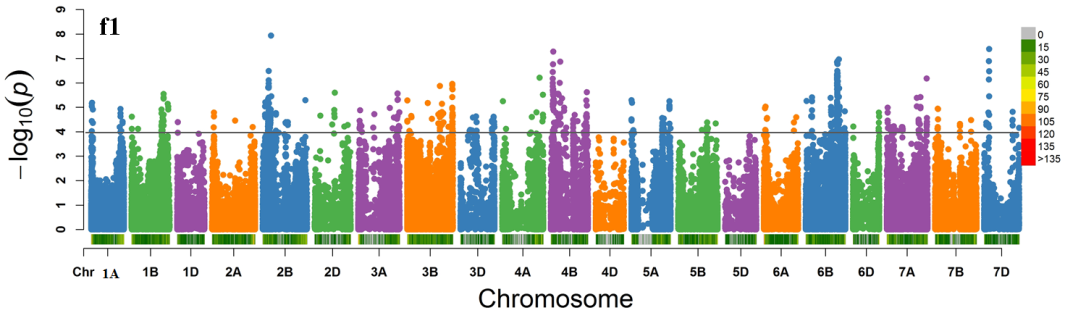

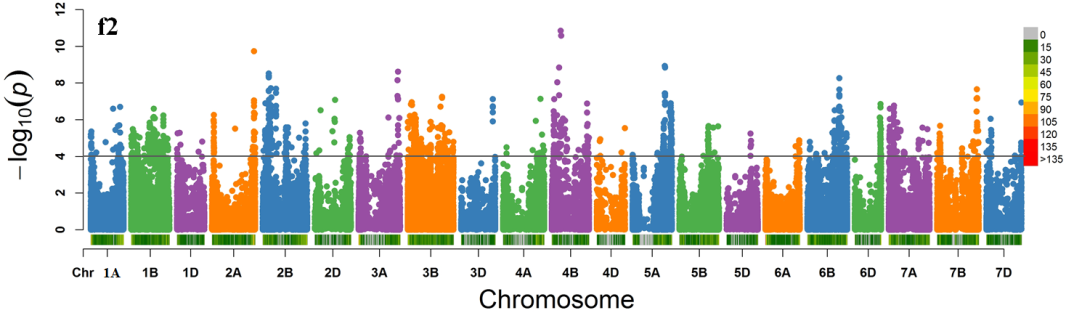

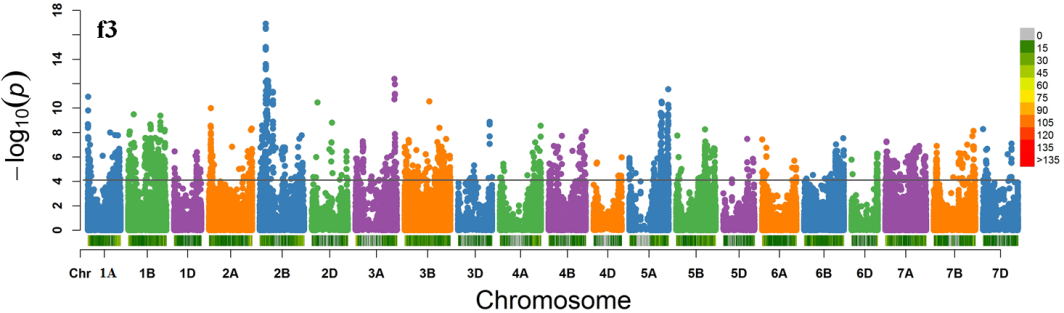

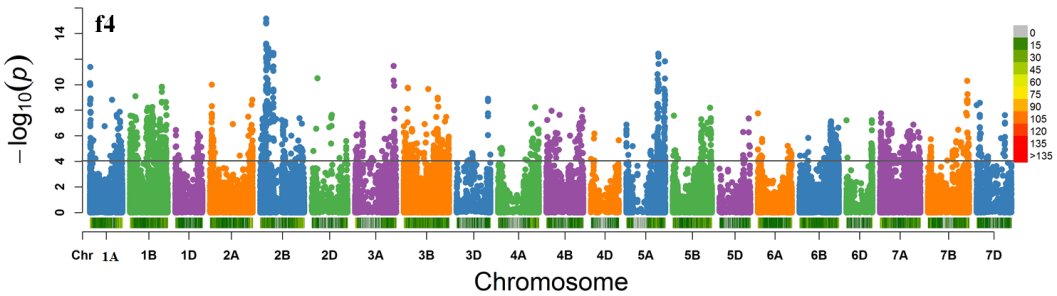

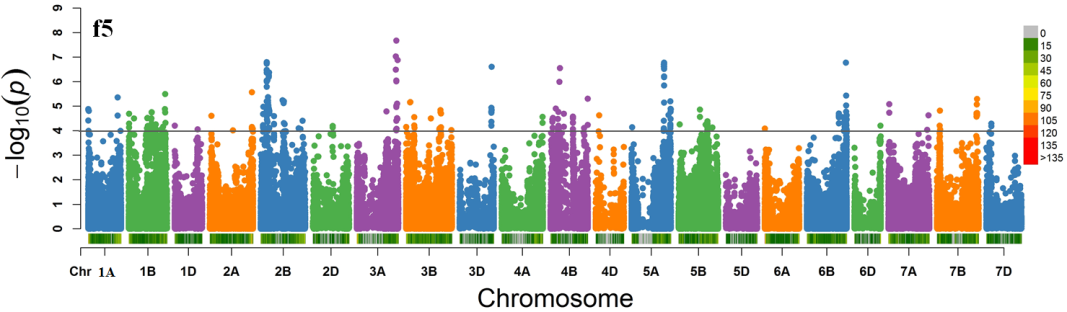

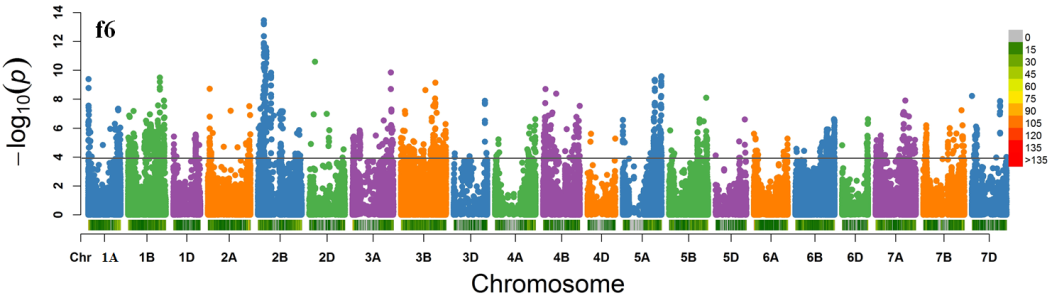

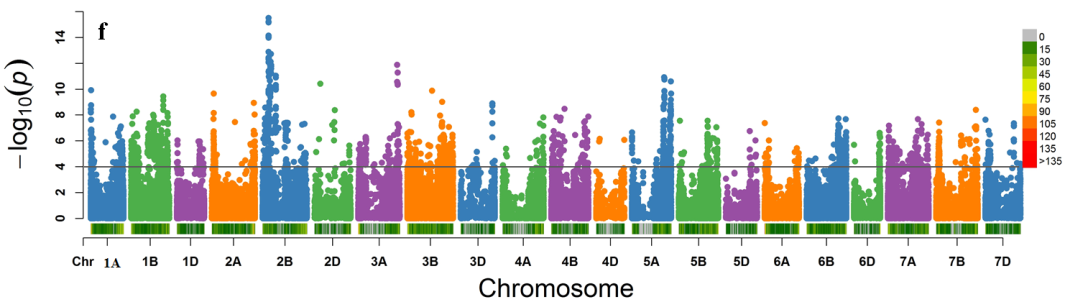


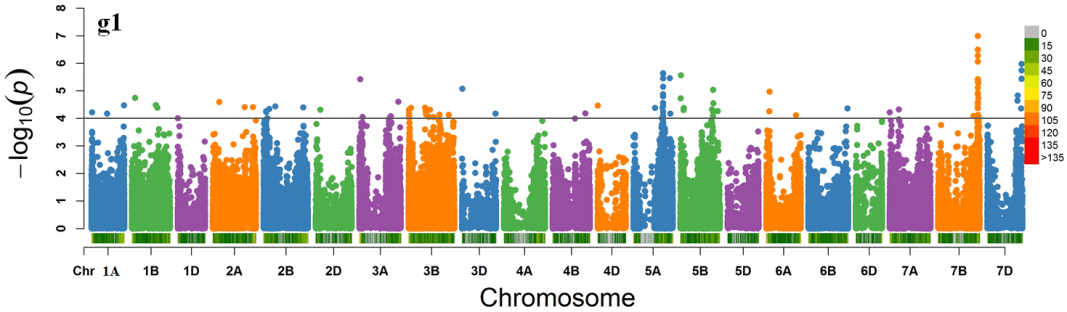

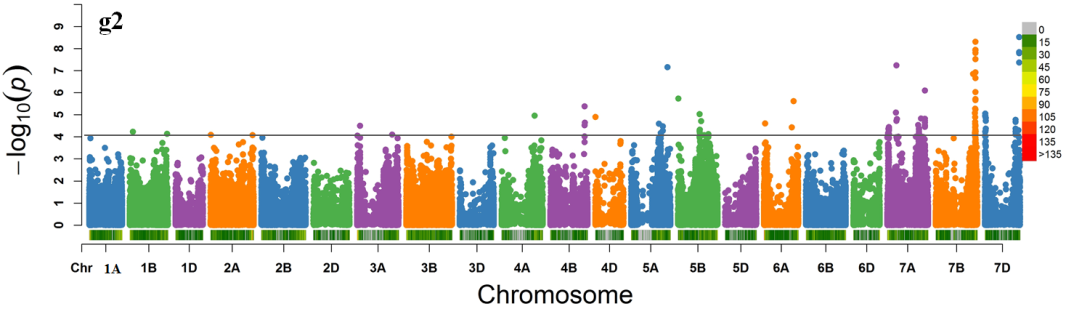

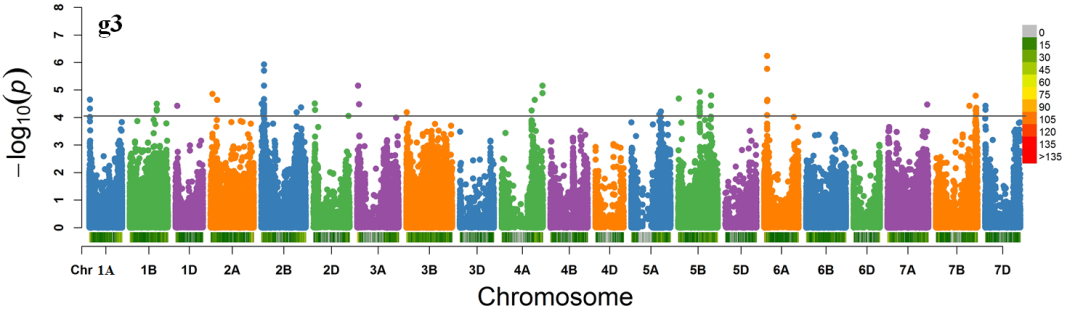

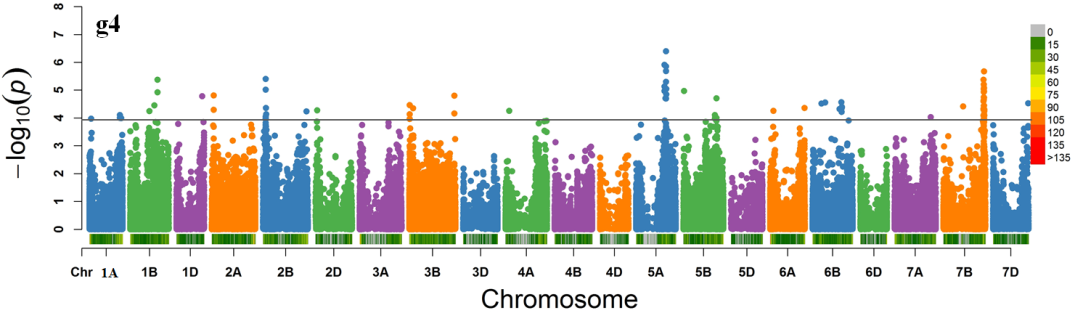

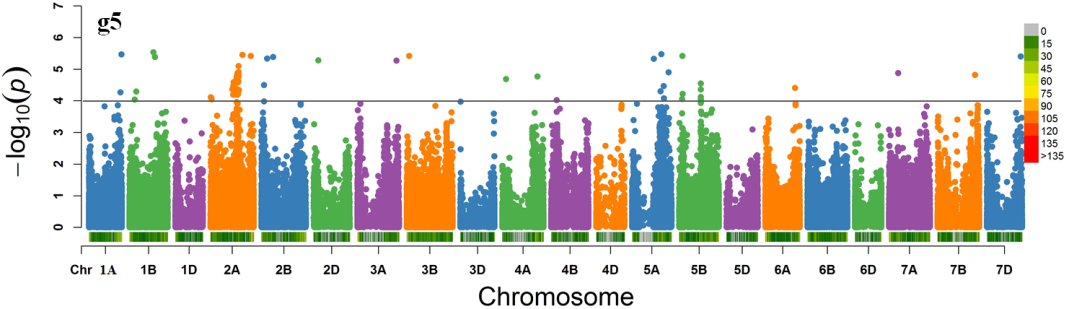

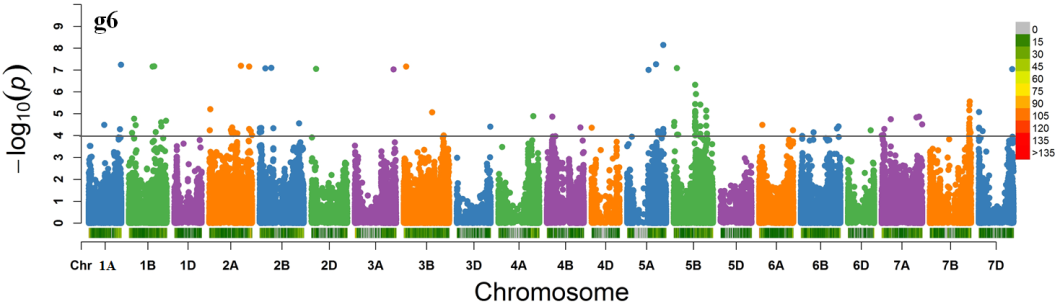

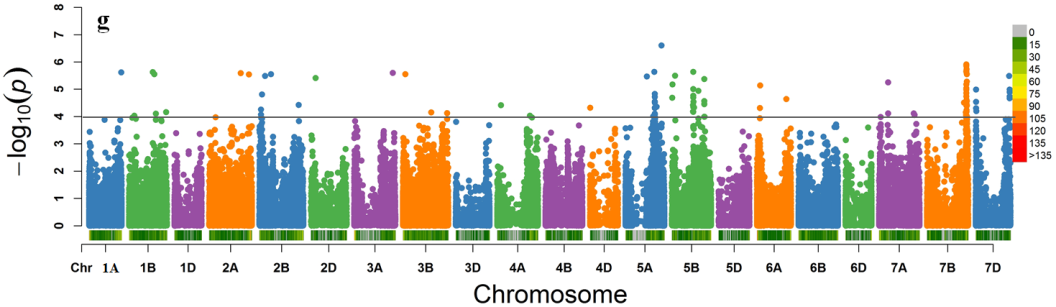


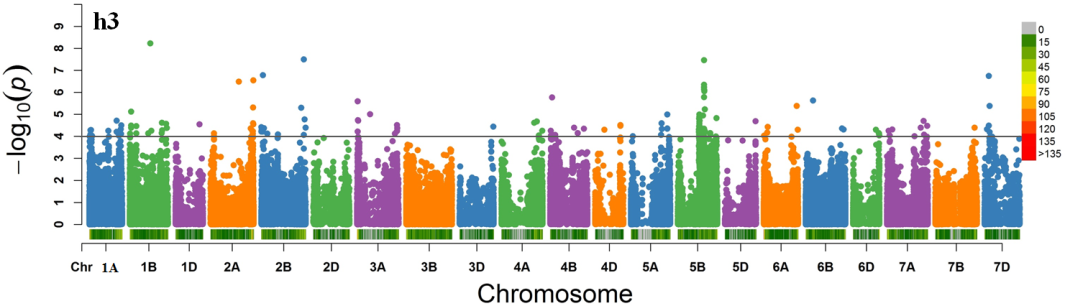

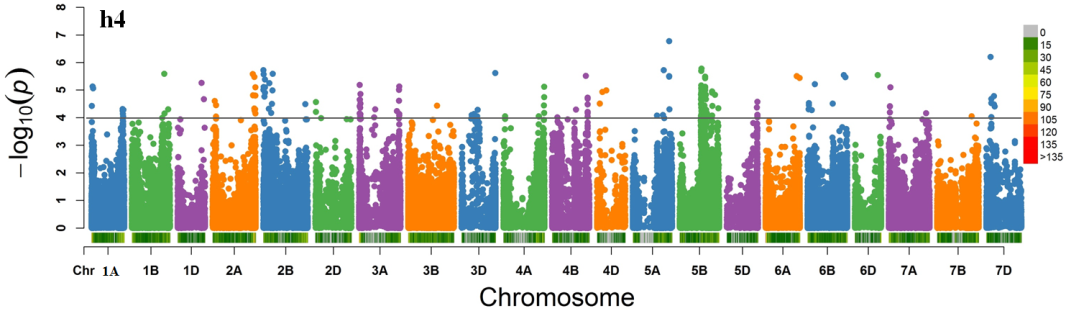

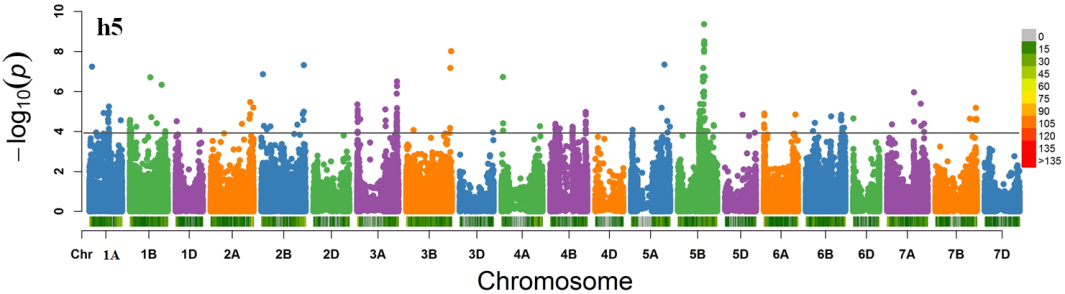

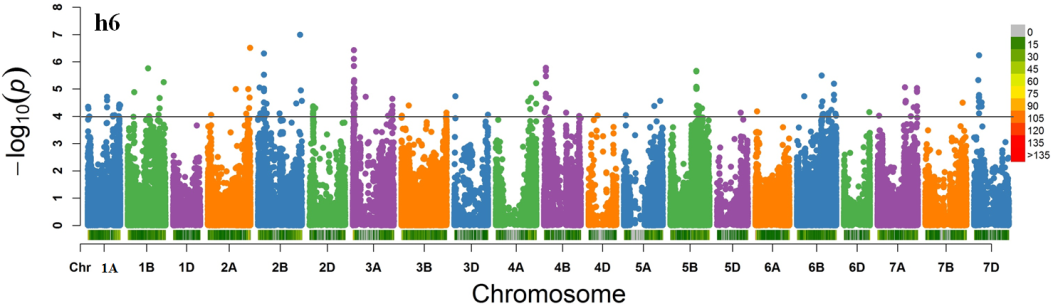

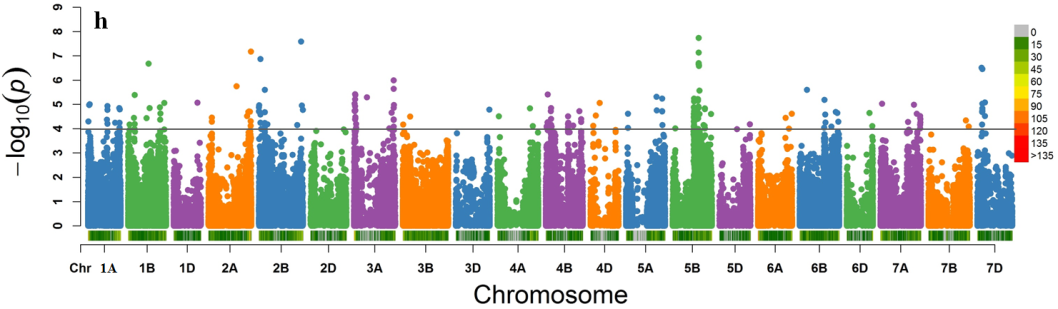


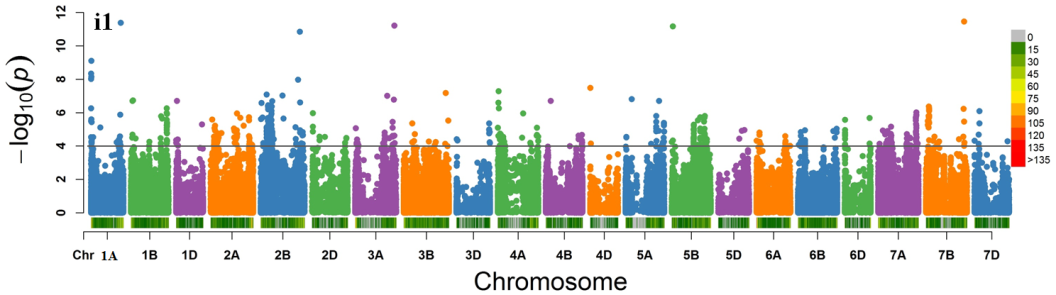

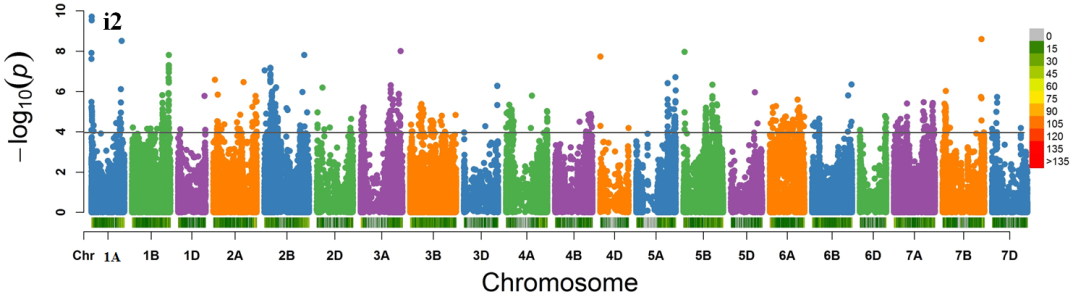

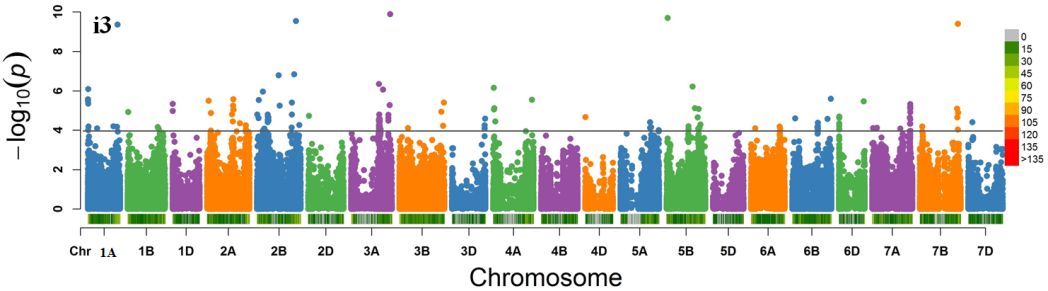

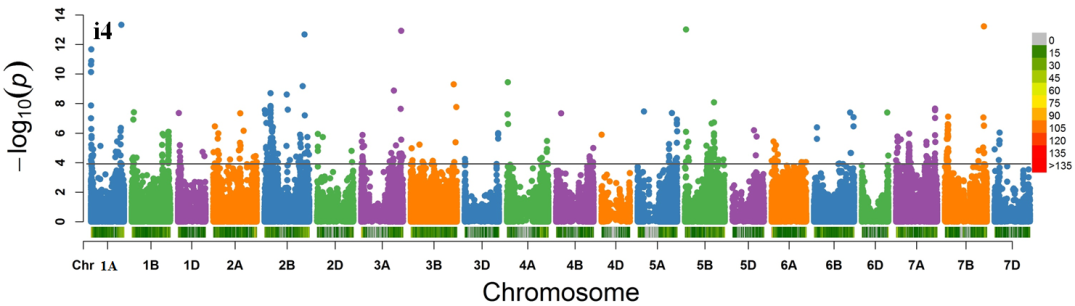

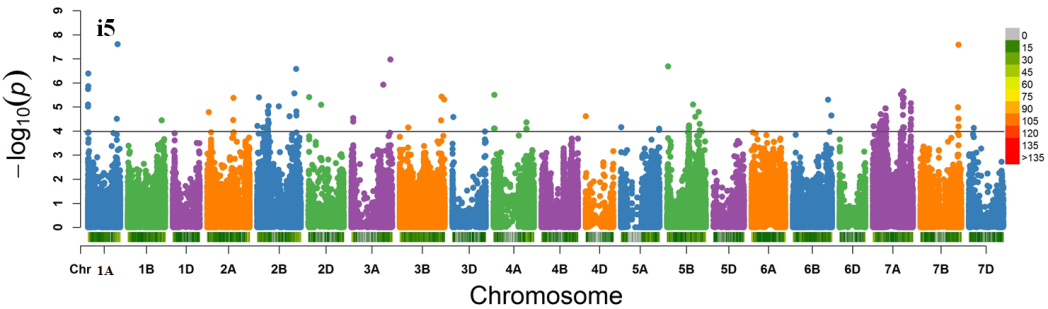

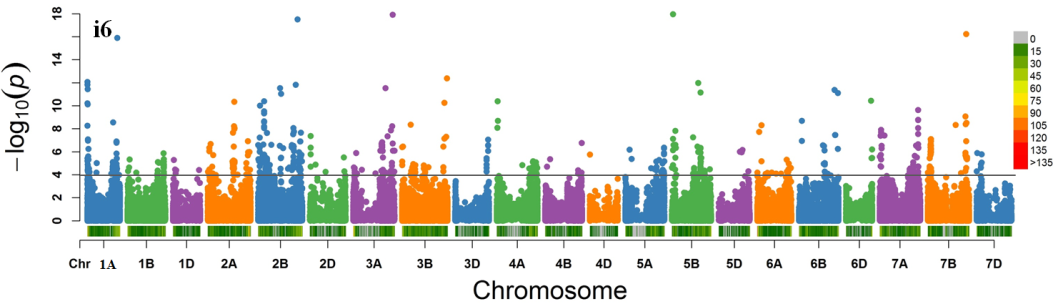

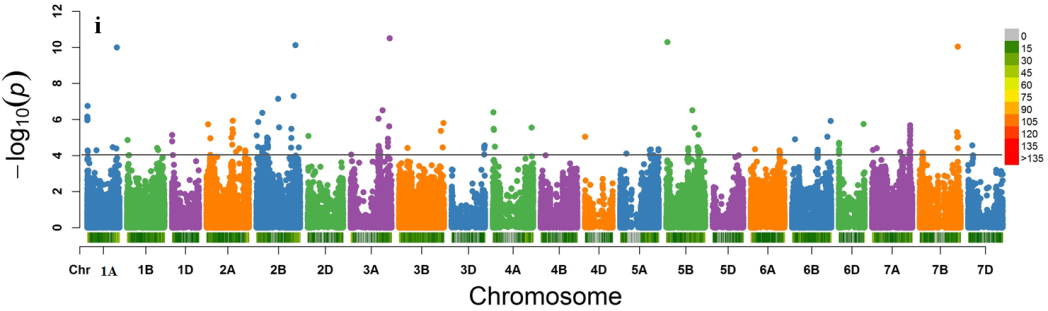


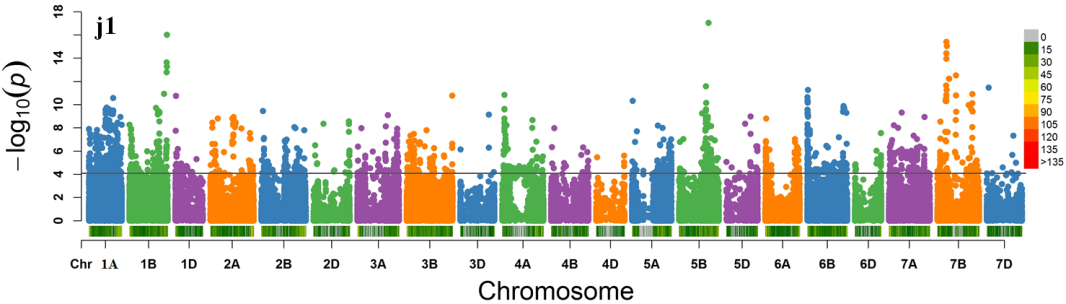

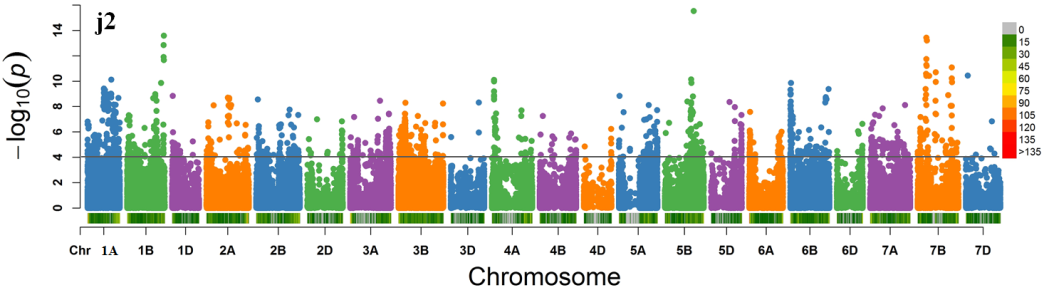

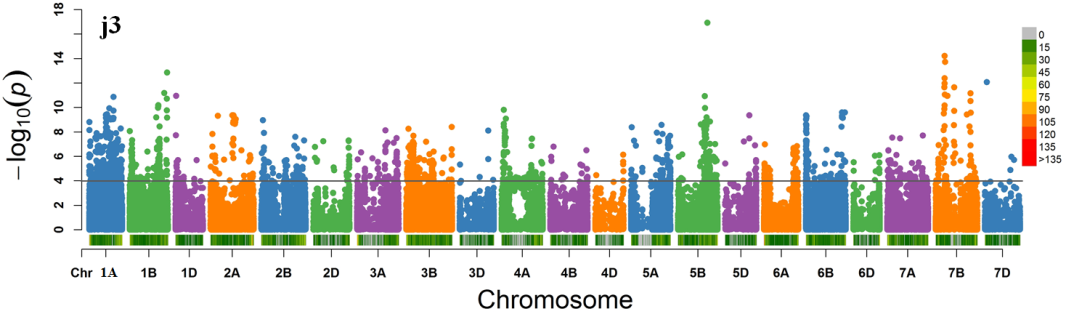

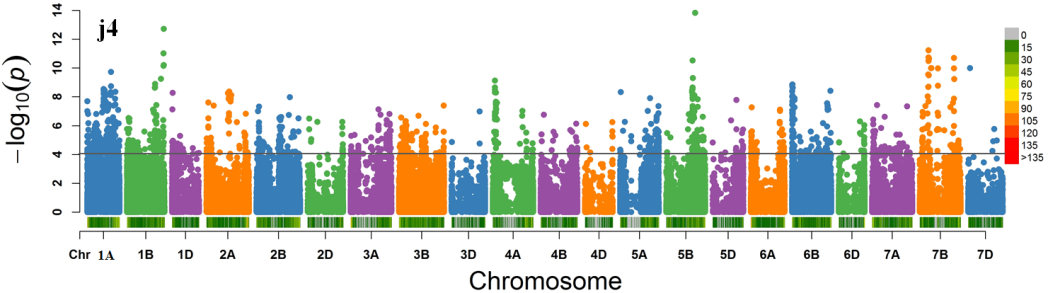

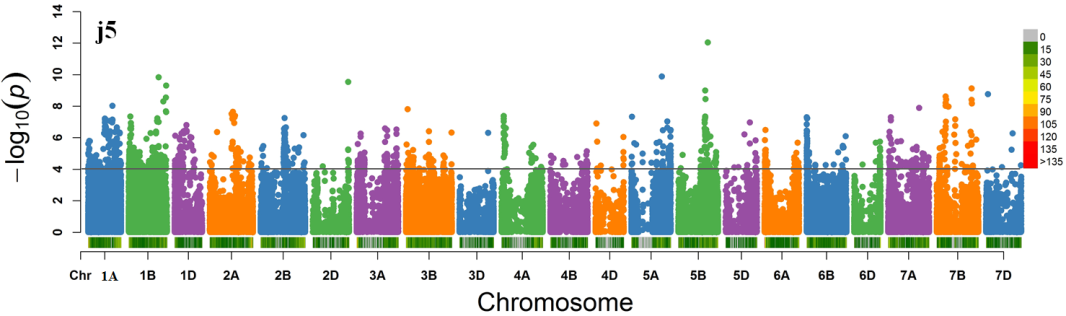

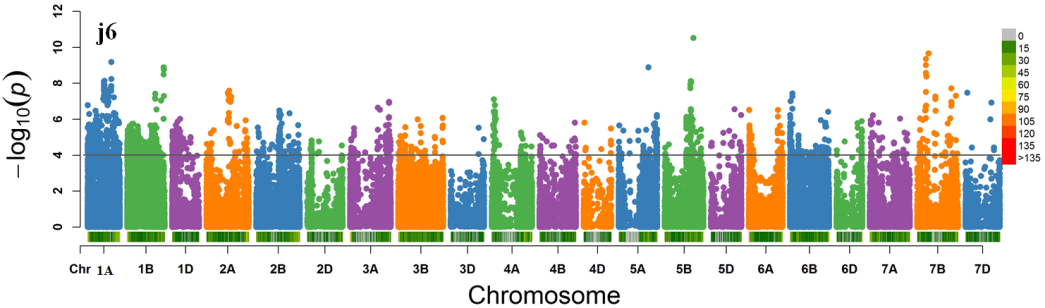

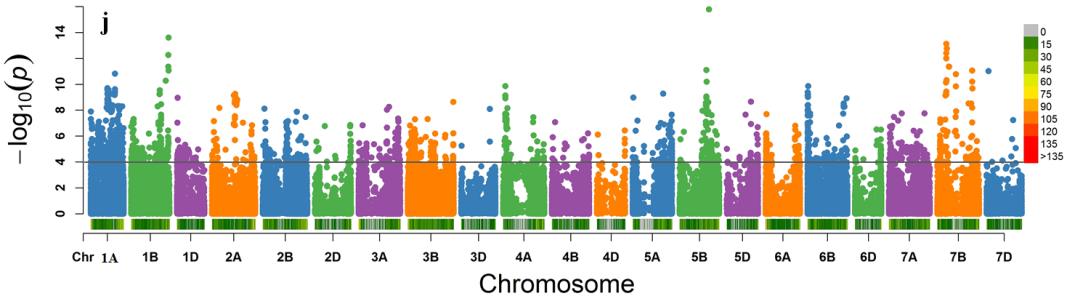


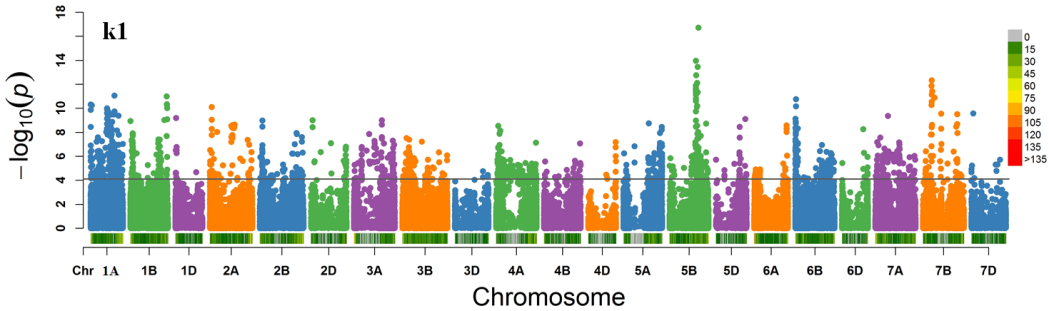

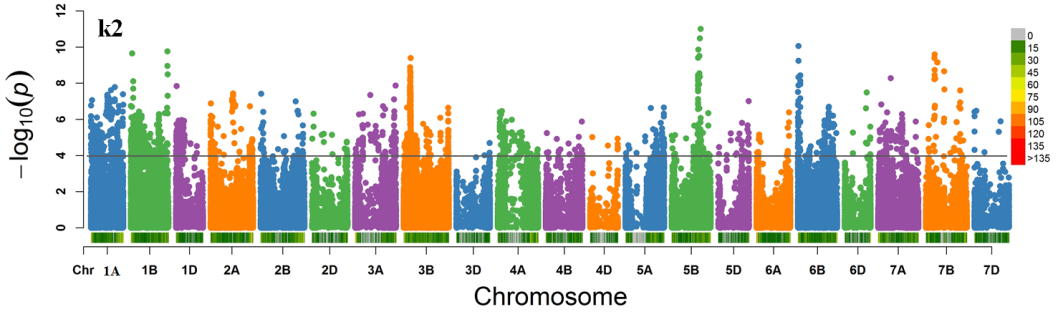

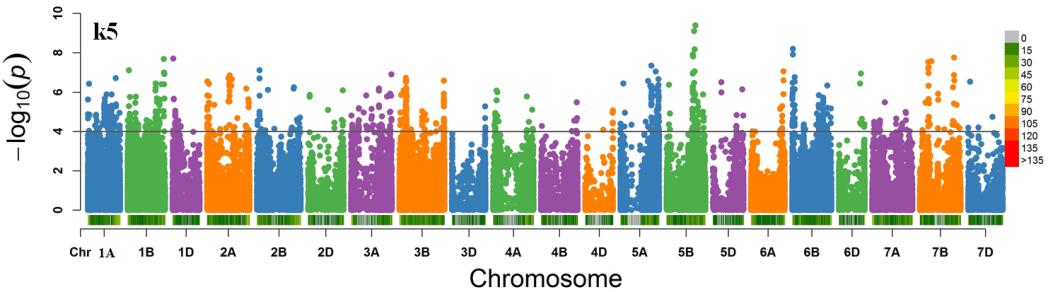

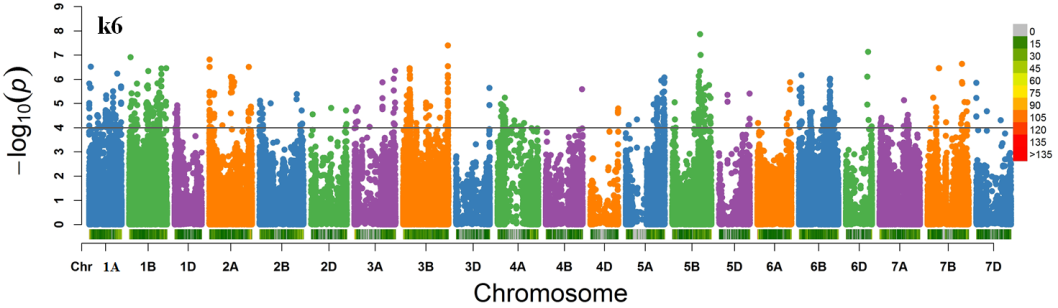

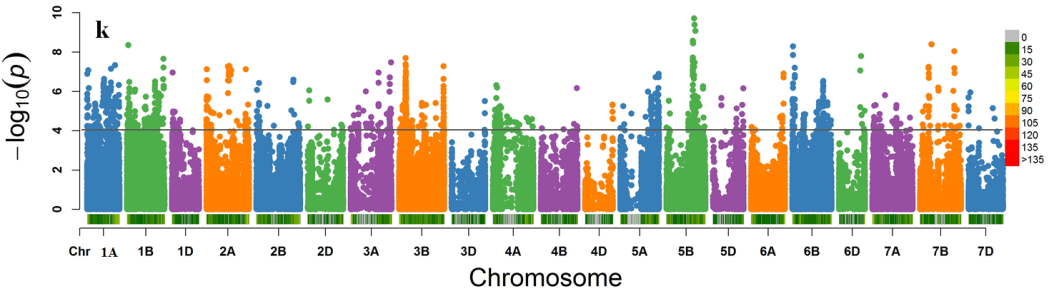


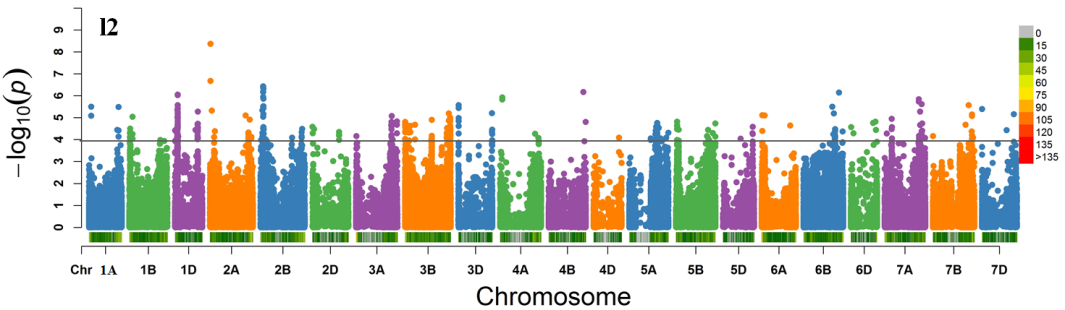

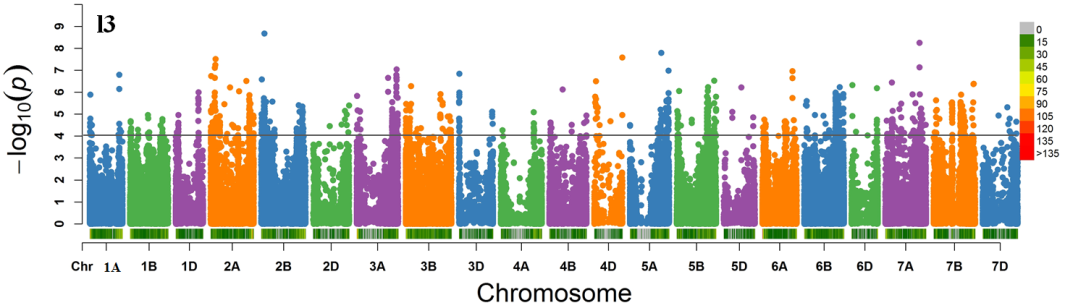

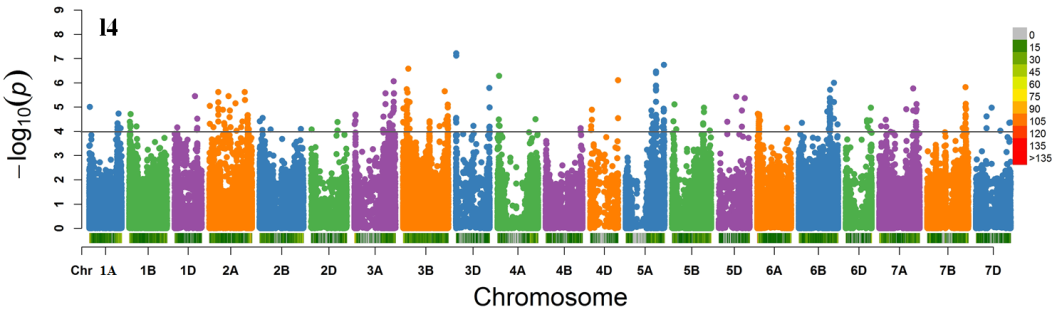

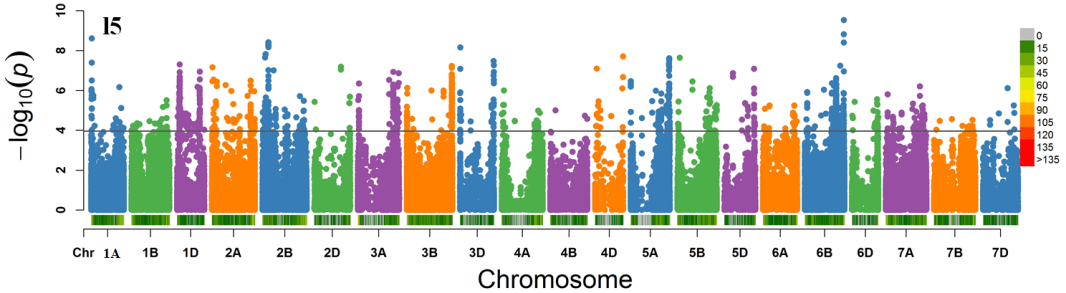

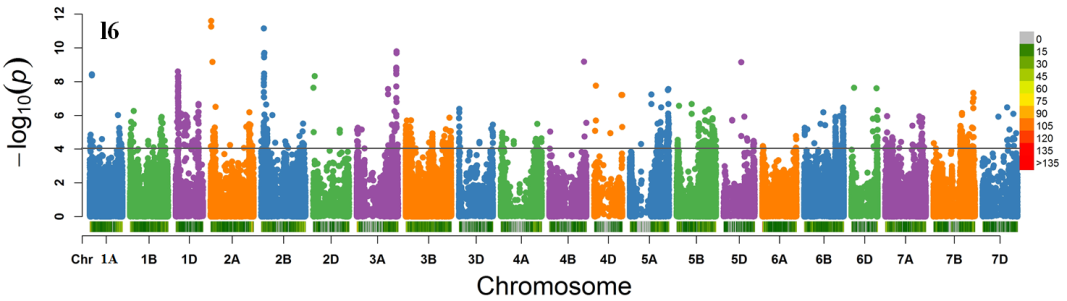

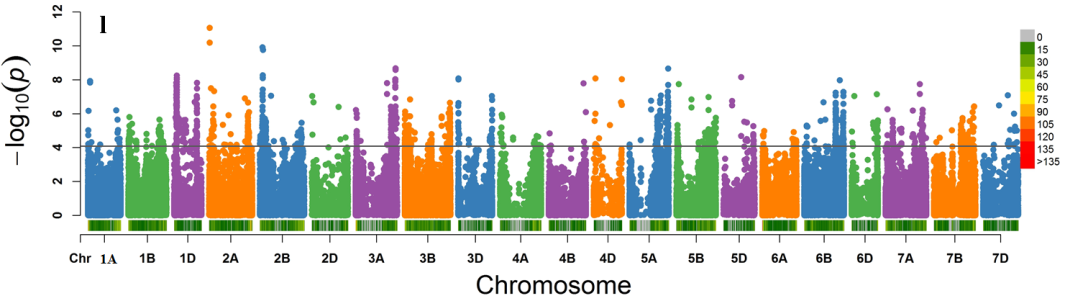


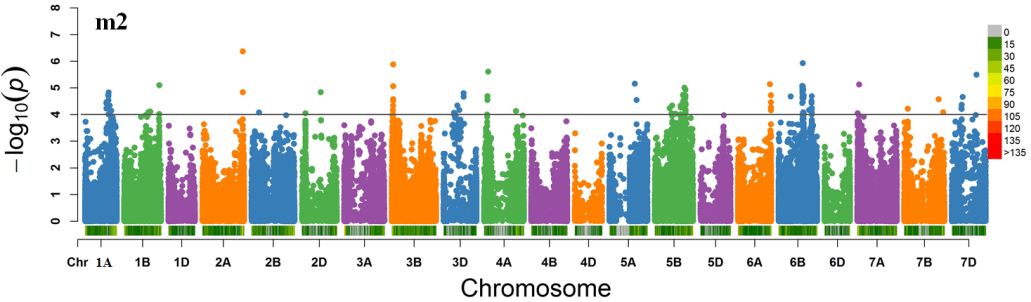

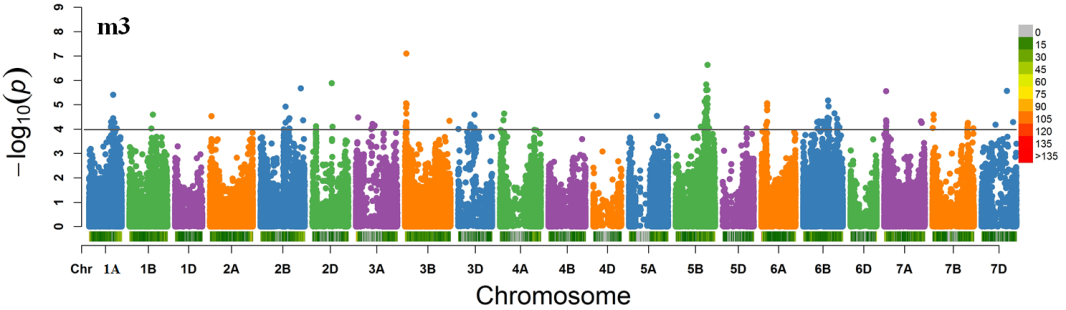

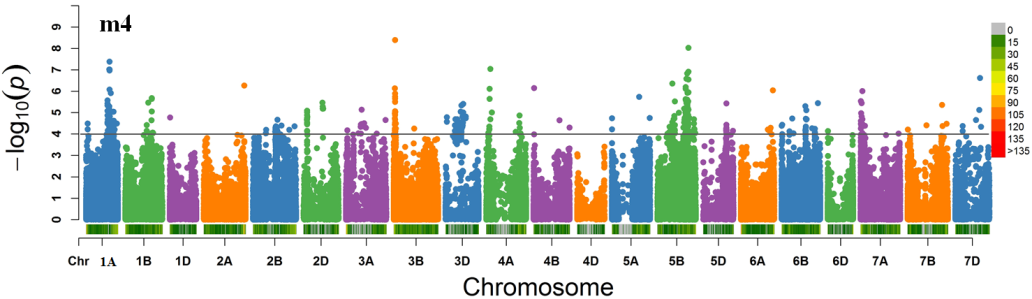

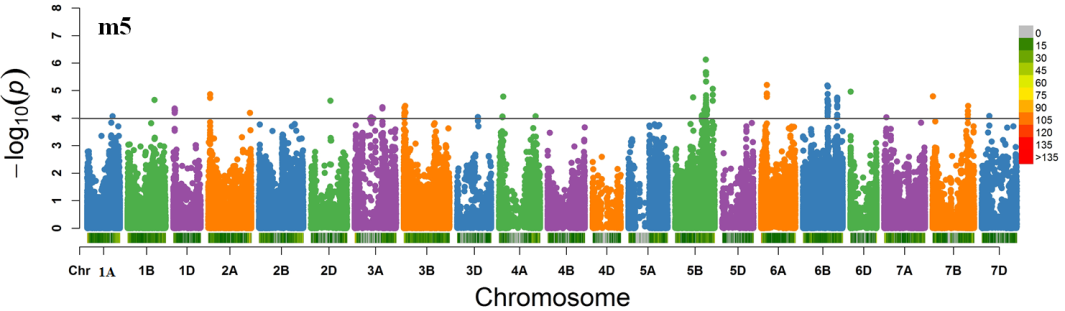

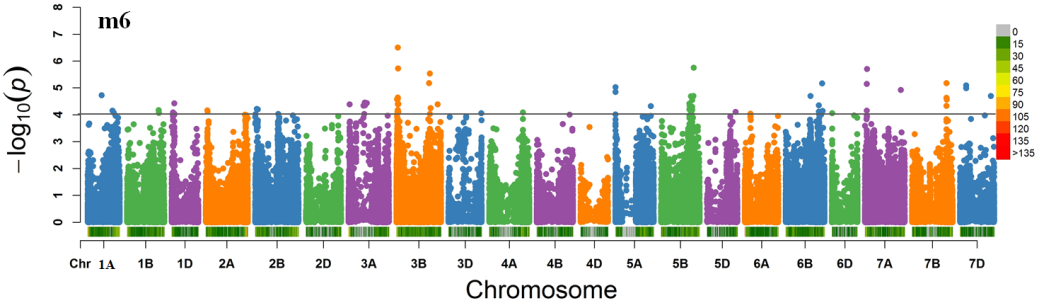

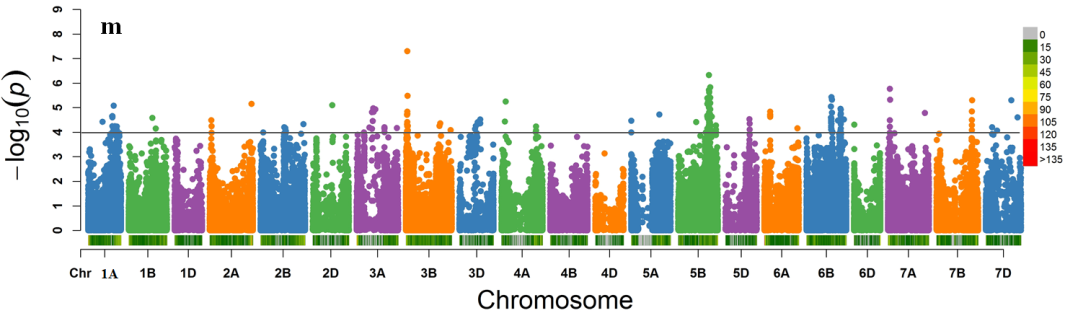


Fig. S4 Manhattan plots for grain yield and related traits in each environment and BLUE value in the diverse panel based on Haplotype-GWAS. See footnote to Fig. S3 for traits and experimental sites.
